# CEMIP, acting as a scaffold protein for bridging GRAF1 and MIB1, promotes colorectal cancer metastasis via activating CDC42/MAPK pathway

**DOI:** 10.1038/s41419-023-05644-z

**Published:** 2023-02-27

**Authors:** Guojie Xu, Lei Zhao, Qingling Hua, Lanqing Wang, Hongli Liu, Zhenyu Lin, Min Jin, Jing Wang, Pengfei Zhou, Kunyu Yang, Gang Wu, Dandan Yu, Dejun Zhang, Tao Zhang

**Affiliations:** 1 Cancer Center, Union Hospital, Tongji Medical College, Huazhong University of Science and Technology, Wuhan, 430022 P.R. China; 2 Institute of Radiation Oncology, Union Hospital, Tongji Medical College, Huazhong University of Science and Technology, Wuhan, 430022 P.R. China; 3 Wuhan YZY Medical Science & Technology Co., Ltd, Wuhan, 430075 P.R. China

**Keywords:** Metastasis, Cancer therapy

## Abstract

Metastasis is the leading cause of treatment failure and tumor-related death in colorectal cancer (CRC). Our previous studies report that CEMIP functionally promotes CRC metastasis and is closely related to poor outcomes. However, the molecular network of CEMIP promoting CRC metastasis is still not fully understood. In the current study, we identify CEMIP interacting with GRAF1, and the combination of high-CEMIP and low-GRAF1 predicts poor survival of patients. Mechanistically, we elucidate that CEMIP interacts with the SH3 domain of GRAF1 through the 295–819aa domain, and negatively regulates the stability of GRAF1. Moreover, we identify MIB1 to be an E3 ubiquitin ligase for GRAF1. Importantly, we uncover that CEMIP acts as a scaffold protein in bridging MIB1 and GRAF1, which is critical to GRAF1 degradation and CEMIP-mediated CRC metastasis. Furthermore, we found that CEMIP activates CDC42/MAPK pathway-regulated EMT by enhancing the degradation of GRAF1, which is indispensable to CEMIP-mediated migration and invasion of CRC cells. Subsequently, we prove that CDC42 inhibitor suppresses CEMIP-mediated CRC metastasis in vitro and in vivo. Collectively, our results reveal that CEMIP promotes CRC metastasis through GRAF1/CDC42/MAPK pathway-regulated EMT, and suggest that CDC42 inhibitor could be a novel therapeutic strategy for CEMIP-mediated CRC metastasis.

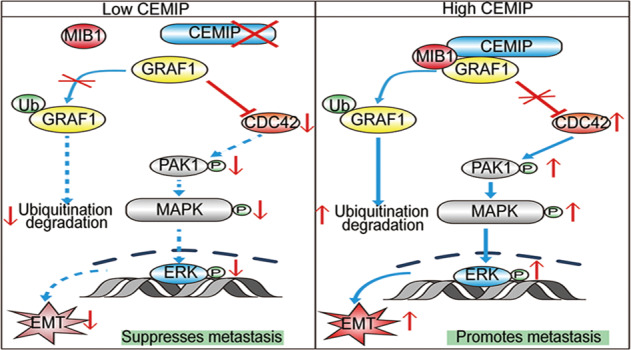

## Introduction

Colorectal cancer (CRC) is the third most common cancer and second most common cause of cancer-related deaths worldwide [1]. Although molecular targeted therapy (*e.g*., bevacizumab, cetuximab) improves the survival of metastatic CRC patients, and PD-1 or CTLA4 inhibitors benefit metastatic CRC patients with d-MMR/MSI-H, metastasis is the leading cause of CRC-related death [2–7]. Unfortunately, the mechanisms of CRC metastasis remain to be elucidated, which impedes the prevention and treatment of CRC.

Recently, it was reported that CEMIP (cell migration-inducing and hyaluronan-binding protein) was associated with migration, invasion and drug resistance in various tumors [8–14], particularly in colon cancer, breast cancer, stomach cancer. For instance, Evensen et al. reported that CEMIP interacted with Bip in endoplasmic reticulum, which led to Ca^2+^ release and activate PKCα signaling, then accelerated metastasis of breast cancer [8]. Rodrigues et al. demonstrated that tumor exosomal CEMIP protein promoted cancer cell colonization in brain metastasis through up-regulation of CCL/CXCL cytokines [10]. In terms of CRC, our previous studies showed that over-expression of CEMIP predicted poor outcomes of CRC patients and was positively correlated with CRC metastasis [15–17], and CEMIP facilitated the infiltration of immunosuppressive neutrophils and drove immune suppression via the TGFβ-CXCL3/1-CXCR2 axis in liver metastasis [18]. Collectively, CEMIP is potential to serve as a therapeutic target for tumor metastasis. However, the underlying molecular network of CEMIP promoting CRC metastasis is still not fully understood.

GRAF1 (also named ARHGAP26) was reported to be a tumor suppressor that was functionally or genetically inactivated in several tumors, including gastric cancer, ovarian cancer, and metastatic brain cancer [19–22]. GRAF1 was comprised of a BAR domain, a PH domain, a RhoGAP domain, and a SH3 domain which was originally identified binding to focal adhesion kinase [23, 24]. And GRAF1 was known as a GTPase activating protein that mediated the activity of GTP binding proteins Rho A and CDC42 [23–25]. Nevertheless, the regulatory mechanism of biological function and expression level of GRAF1 in tumorigenesis remained elusive.

In this study, we identify CEMIP interacting with GRAF1, and the combination of high-CEMIP and low-GRAF1 predicts poor survival of patients. Mechanistically, we elucidated that the 295–819aa domain of CEMIP interacted with the SH3 domain of GRAF1, and negatively regulates the stability of GRAF1. Moreover, we uncovered that CEMIP acted as a scaffold protein in bridging MIB1 (an E3 ubiquitin ligase) and GRAF1, which was critical to GRAF1 degradation. Furthermore, we found that CEMIP promoted CRC metastasis by enhancing the degradation of GRAF1 and activating CDC42/MAPK pathway-regulated EMT. Subsequently, we proved that CDC42 inhibitor suppressed CEMIP-mediated CRC metastasis in vitro and in vivo. Collectively, our results suggested that CDC42 inhibitor could be a novel therapeutic strategy for CEMIP-mediated CRC metastasis.

## Results

### Identification of GRAF1 as a binding protein for CEMIP

**Fig. 1:**
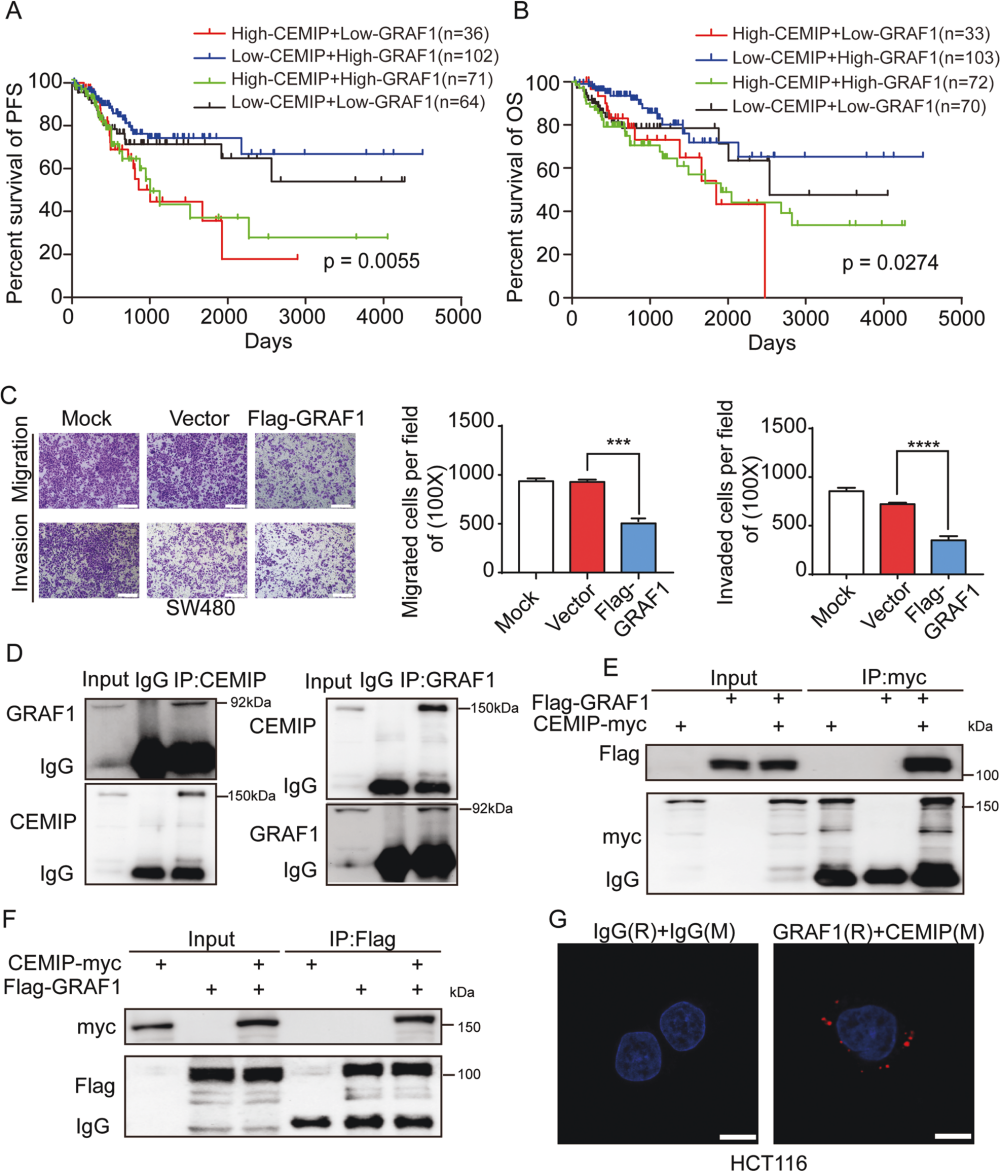
CEMIP has negative correlation with GRAF1 in CRC. **A**, **B** The Progression Free survival (PFS, **A**) and Overall Survival (OS, **B**) of colon cancer patients were generated through TCGA (The Cancer Genome Atlas) database from http://www.sangerbox.com/. **C** Transwell chamber migration and invasion of SW480 cells transfected with Flag-GRAF1(GRAF1-overexpression plasmid with Flag tag attached in N terminal of GRAF1) or Scramble plasmid (negative control). ***P < 0.001; ****P < 0.0001. Scale bar, 50 μm. **D** The interactions between endogenous CEMIP and endogenous GRAF1 were detected by Co-IP in HCT116 cells. **E**, **F** The interactions between exogenous CEMIP and exogenous GRAF1 were detected by Co-IP in HEK293T cells transfected with the indicated constructs. **G** The interaction between endogenous GRAF1 and CEMIP in HCT116 cells was further confirmed by the proximity ligation assays (PLA). Scale bar, 10 μm.

Our previous studies demonstrated that the elevated expression of CEMIP was closely related to poor outcomes and promoted CRC metastasis [15–18]. To elucidate the molecular mechanism network involved in CEMIP-mediated CRC metastasis, we conducted co-immunoprecipitation (Co-IP) and mass-spectrometric (MS) peptide sequencing to identify CEMIP-interacting proteins in cells. In view of the MS score (top 5) and biological function of the potential CEMIP-binding proteins, we were interested in GRAF1 (Supplementary Fig. S1A, B and Supplementary Table S1). GRAF1 had been proved to be a cancer suppressor which inhibited the progression of gastric, ovarian, lung and colorectal cancer [19–22, 26]. Our bioinformatics analyses showed that CRC patients with high-GRAF1 had better overall survival (OS) than patients with low-GRAF1 but not progression-free survival (PFS) (Supplementary Fig. S1C), while CRC patients with high-CEMIP had shorter PFS than patients with low-CEMIP but not OS (Supplementary Fig. S1D). It indicated that GRAF1 or CEMIP alone could not predict both of PFS and OS of patients. However, we found that CRC patients with high-CEMIP + low-GRAF1, high-CEMIP + high-GRAF1, had worse PFS and OS than patients with low-CEMIP + high-GRAF1, low-CEMIP + low-GRAF1 respectively (Fig. 1A, B), suggesting that the combination of CEMIP and GRAF1 could effectively predict the survival of patients. Moreover, our results confirmed that GRAF1 inhibited the migration and invasion of CRC cells in vitro (Fig. 1C and Supplementary Fig. S1E). Therefore, these data uncovered GRAF1 as a promising candidate of CEMIP-interacting protein.

To further validate the interaction between CEMIP and GRAF1, we performed a series of Co-IP experiments. As showed in Fig. 1D, the endogenous interaction between CEMIP and GRAF1 was observed in CRC cells. Furthermore, exogenous CEMIP was able to bind to exogenous GRAF1, and vice versa (Fig. 1E, F). Importantly, the Duolink proximity ligation assays further confirmed a direct linkage between CEMIP and GRAF1 (Fig. 1G). Collectively, these findings strongly suggested that CEMIP physically interacted with GRAF1 directly.

### CEMIP interacts with the SH3 domain of GRAF1 through the 295–819aa domain

To further investigate which domain of CEMIP was required for binding to GRAF1, we constructed six truncated variants of CEMIP with a C-terminal myc tag (CEMIP-myc-∆1: 1–303aa; CEMIP-myc-∆2: 295–591aa; CEMIP-myc-∆3: 572–819aa; CEMIP-myc-∆4: 820–1204aa; CEMIP-myc-∆5: 1205–1361aa; CEMIP-myc-∆2 + 3: 295–819aa), as indicated in Supplementary Fig. S2A. Each of truncated CEMIP variants and GRAF1 plasmids were co-expressed in HEK293T cells respectively. Then the co-immunoprecipitation assay was performed using the whole cell lysates. As illustrated in Supplementary Fig. S2B, C, CEMIP-myc-∆2, CEMIP-myc-∆3 and CEMIP-myc-∆2 + 3 were observed to bind to GRAF1. Therefore, these results demonstrated that the 295–819aa domain of CEMIP was indispensable for binding to GRAF1.

It was reported that, the SH3 domain of GRAF1 was conserved in different species (Supplementary Fig. S3A) and could be interacted with various proteins such as CDC42 and TGF-βR, which was crucial for the downstream signaling [23, 27]. To further determine whether the SH3 domain of GRAF1 was required for binding to CEMIP, we generated the GRAF1 deletion mutant without the SH3 domain (GRAF1-∆SH3), as indicated in Supplementary Fig. S3B [23]. The GRAF1-∆SH3 variant and CEMIP plasmids were co-expressed in HCT116 cells, then the co-immunoprecipitation assay was performed. The results showed that GRAF1-∆SH3 could not bind to CEMIP (Supplementary Fig. S3C–E), which indicated that GRAF1 interacted with CEMIP via the SH3 domain. Together, these results demonstrated that the 295–819aa domain of CEMIP interacts with the SH3 domain of GRAF1.

### CEMIP negatively regulates the stability of GRAF1

**Fig. 2:**
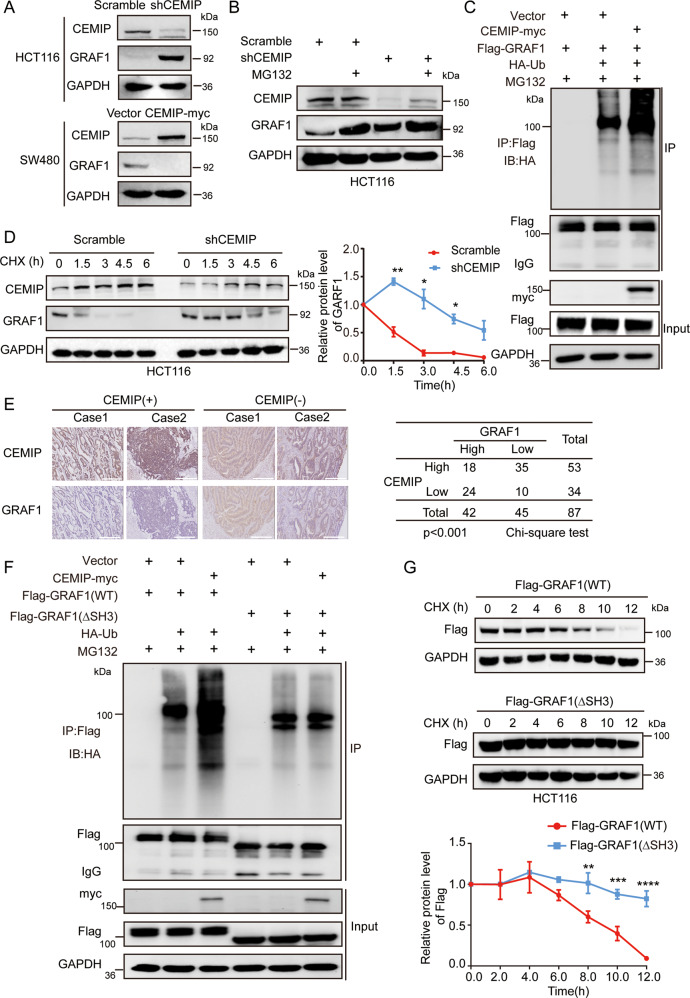
CEMIP negatively regulates the stability of GRAF1. **A** Western blotting analysis of HCT116 cells transfected with shCEMIP (CEMIP-downregulated plasmid containing shRNA of CEMIP) and western blotting analysis of SW480 cells transfected with CEMIP-myc (CEMIP-upregulated plasmid containing CEMIP cDNA with c-myc tag attached in C terminal of CEMIP). **B** Western blotting analysis of CEMIP and GRAF1 in HCT116 cells transfected with the indicated plasmids with or without MG132 (26 S proteasome inhibitor, 10 μM, 6 h). **C** Western blotting analysis of the ubiquitination level of GRAF1 derived from Co-IP in HCT116 cells transfected with the indicated constructs. **D** HCT116 cells transfected with the indicated plasmid were treated with CHX (cycloheximide, 20 μg/mL) for the indicated times, and the expressions of GRAF1 were analyzed by western blotting analysis, *P < 0.05; **P < 0.01. **E** Representative immunohistochemical staining of CEMIP and GRAF1 in human primary colorectal carcinoma. Scale bar, 50 μm. Statistical analysis of immunohistochemical staining of two proteins. **F** Western blotting analysis of the ubiquitination level of GRAF1 and GRAF1 (∆SH3) derived from Co-IP in HCT116 cells transfected with the indicated constructs. **G** HCT116 cells transfected with the indicated plasmids were treated with CHX (20 μg/mL) for the indicated times, and the expressions of GRAF1 and GRAF1 (∆SH3) were analyzed by western blotting analysis. **P < 0.01; ***P < 0.001; ****P < 0.0001.

Having proved a physical interaction between the two molecules, we next evaluated the effect of CEMIP on GRAF1. As shown in Fig. 2A, knock-down of CEMIP dramatically resulted in the accumulation of endogenous GRAF1, while exogenously expressed CEMIP resulted in the decrease of endogenous GRAF1. Interestingly, no obvious variations were observed between the mRNA levels of GRAF1 when CEMIP was down-regulated or up-regulated (Supplementary Fig. S4). Moreover, no matter whether down-regulating CEMIP level in cells, treatment with MG132 (proteasome inhibitor) led to increased GRAF1 protein level (Fig. 2B), which suggested that CEMIP probably regulated the protein stability of GRAF1 through the ubiquitin/proteasome system. In line with the above observation, ubiquitination assays showed that exogenously expressed CEMIP led to the increased ubiquitination level of GRAF1 (Fig. 2C), and knock-down of CEMIP remarkably increased the half-life of GRAF1 (Fig. 2D). Furthermore, we detected the protein levels of CEMIP and GRAF1 in CRC tissues by immunohistochemistry (IHC). The results indicated that there was a negative correlation between CEMIP and GRAF1 (Fig. 2E, and Supplementary Tables S2,S3). Importantly, considering that CEMIP bond to the SH3 domain of GRAF1, we found that CEMIP promoted the ubiquitination level and reduced the half-life of wild GRAF1 but not the mutated GRAF1 without SH3 domain (Fig. 2F, G), indicating that the interaction of CEMIP with GRAF1 was indispensable for the stability of GRAF1. Therefore, these findings demonstrated that CEMIP negatively regulated the stability of GRAF1 by enhancing its ubiquitination and degradation.

### Identification of MIB1 as an E3 ubiquitin ligase for GRAF1

**Fig. 3:**
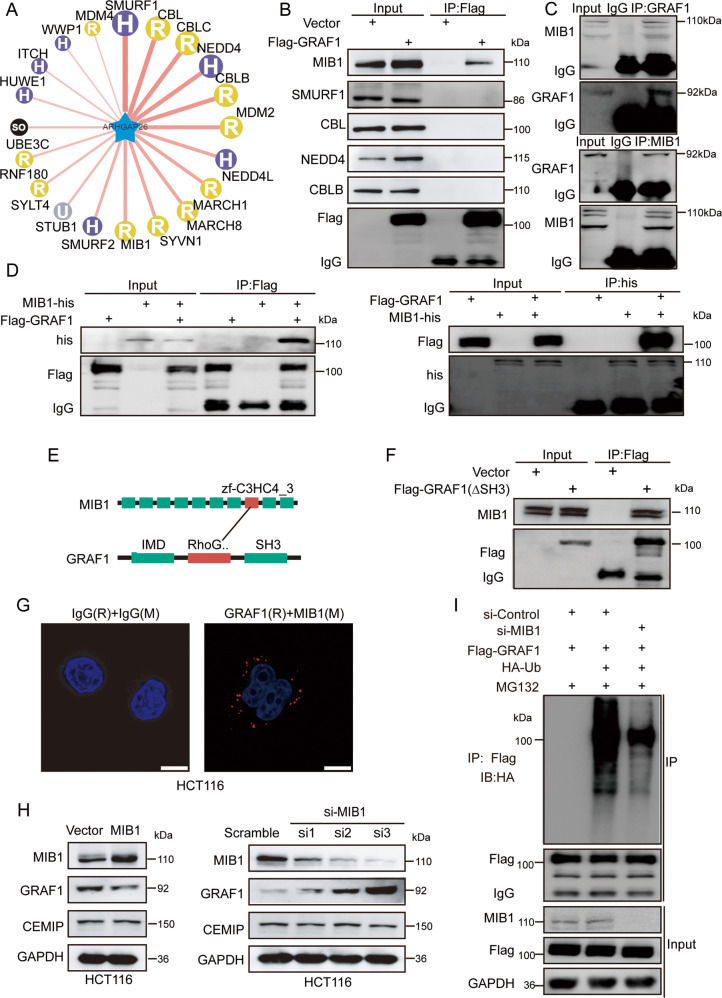
Identification of MIB1 as a novel E3 ubiquitin ligase for GRAF1. **A** Potential E3 ubiquitin ligases of GRAF1 were predicted by the UbiBrowser database. **B** Western blotting analysis of the predicted ligases derived from Co-IP in HCT116 cells transfected with the indicated constructs. **C** The interaction between endogenous GRAF1 and endogenous MIB1 was detected by Co-IP in HCT116 cells. Rabbit IgG was used as a negative control. **D** The interaction between exogenous GRAF1 and exogenous MIB1 was detected by Co-IP in HEK293T cells transfected with the indicated constructs. **E** The putative binding domain between GRAF1 and MIB1 predicted by the database mentioned above from (http://ubibrowser.bio-it.cn/ubibrowser/strict/index/edgeinfo/sub/Q9UNA1/e3/Q86YT6). **F** The interaction between exogenous GRAF1 (∆SH3) and endogenous MIB1 were detected by Co-IP in HCT116 cells. **G** The interaction between endogenous GRAF1 and MIB1 was further confirmed by the PLA assay in HCT116 cells. Scale bar, 10 μm. **H** The protein level of CEMIP and GRAF1 detected by western blotting transfected with MIB1 plasmid (MIB1-upregulated plasmid containing MIB1 cDNA) or si-MIB1 (small interfering RNA of MIB1) in HCT116 cells. **I** Western blotting analysis of the ubiquitination level of GRAF1 derived from Co-IP in HCT116 cells transfected with the indicated constructs.

Given that CEMIP promoted the ubiquitination and degradation of GRAF1, we wondered whether CEMIP was a novel E3 ubiquitin ligase for GRAF1. However, we found no canonical catalytic structure of E3 ubiquitin ligase in the domain of CEMIP by analyzing the amino acid sequence. Hence, we hypothesized that CEMIP regulated the ubiquitination level of GRAF1 by relying on an E3 ubiquitin ligase. To verify this hypothesis, we retrieved the UbiBrowser database (http://ubibrowser.ncpsb.org/) and screened the potential E3 ubiquitin ligases of GRAF1 (Fig. 3A and Supplementary Fig. S5A). Then we performed Co-IP assays to test which E3 ligase binding to GRAF1. However, we found none of top 5 potential E3 ligases binding to GRAF1. Fortunately, we changed the detection strategy and found MIB1, the 11th potential E3 ligases, could bind to GRAF1 (Fig. 3B). Hence MIB1 was regarded as the target of our research. Moreover, we showed that MIB1 interacted with GRAF1 both at the exogenous and endogenous level (Fig. 3B–D and Supplementary Fig. S5B). Importantly, as showed in Fig. 3E, F, MIB1 was predicted to binding the RhoGAP domain of GRAF1 in the UbiBrowser database, and the Co-IP assays showed that MIB1 could bind to the mutated GRAF1 without SH3 domain, indicating that MIB1 may interact with the RhoGAP domain of GRAF1 but not SH3 domain. Furthermore, the Duolink proximity ligation assays confirmed a direct linkage between GRAF1 and MIB1 (Fig. 3G). Therefore, these results demonstrated that MIB1 was a candidate E3 ubiquitin ligase for GRAF1.

Notably, we found that MIB1 regulated the protein level but not the mRNA level of GRAF1, while MIB1 had no effect on both protein and mRNA level of CEMIP (Fig. 3H and Supplementary Fig. S5C, D). And knock-down of MIB1 significantly decreased the ubiquitination level of GRAF1 (Fig. 3I). Collectively, these findings strongly suggested that MIB1 was an E3 ubiquitin ligase for GRAF1.

### CEMIP acts as a scaffold protein in bridging GRAF1 and MIB1, which is critical to GRAF1 degradation

**Fig. 4:**
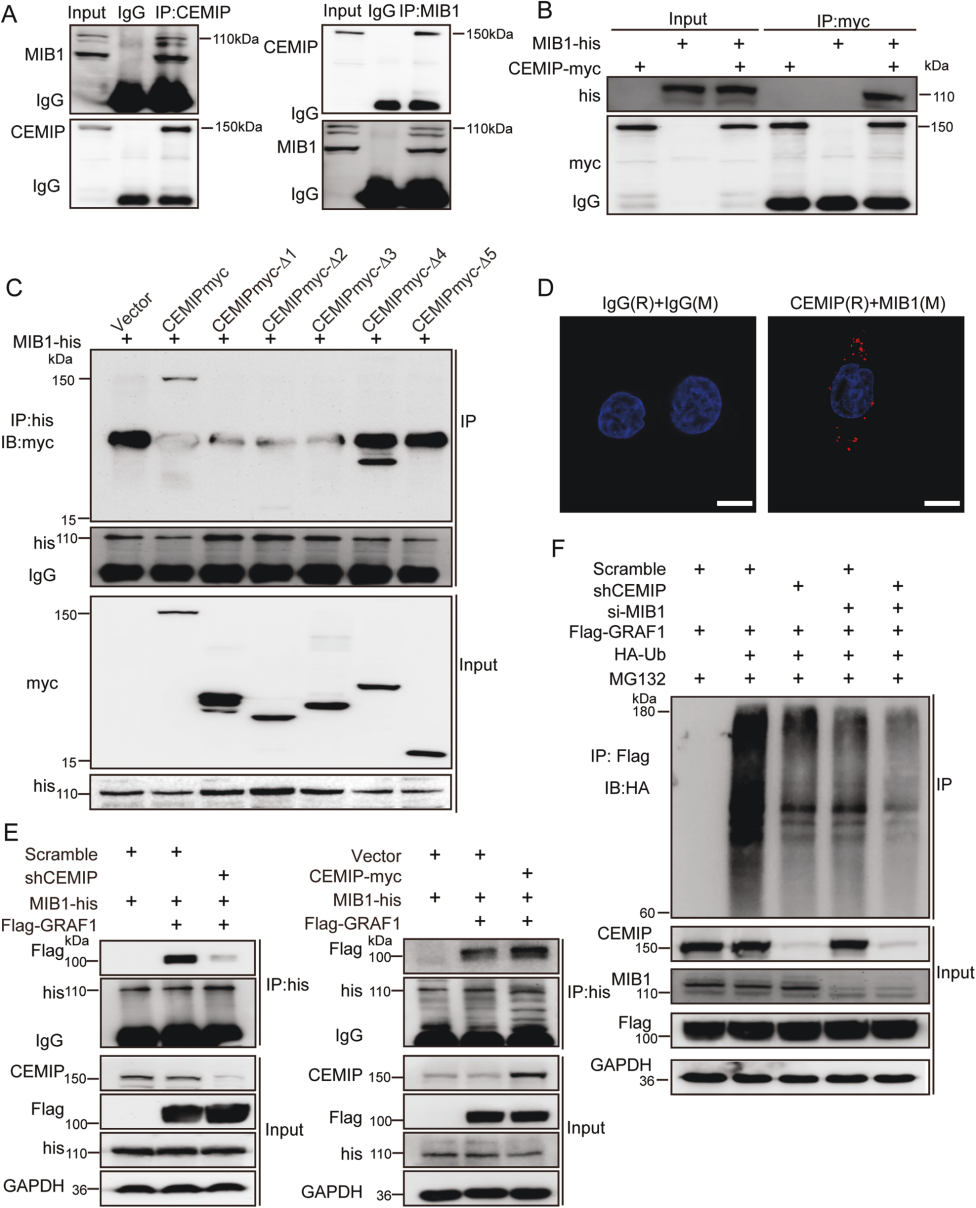
CEMIP acts as a scaffold protein in bridging GRAF1 and MIB1 which is critical to GRAF1 degradation. **A** The interactions between endogenous CEMIP and endogenous MIB1 were detected by Co-IP in HCT116 cells. Rabbit IgG was used as a negative control. **B** The interaction between exogenous CEMIP and exogenous MIB1 was detected by Co-IP in HEK293T cells. **C** The interactions between exogenous CEMIP deletion mutants and exogenous MIB1 were detected by Co-IP in HEK293T cells transfected with the indicated constructs. **D** The interaction between endogenous CEMIP and MIB1 was further confirmed by the PLA assay in HCT116 cells. Scale bar, 10 μm. **E** Western blotting analysis of proteins derived from Co-IP in HCT116 (left) and SW480 cells (right) transfected with the indicated plasmids. **F** Western blotting analysis of the ubiquitination level of GRAF1 derived from Co-IP in HCT116 cells transfected with the indicated constructs.

Based on these findings, we hypothesized that CEMIP acted as an adaptor protein in promoting the interaction of GRAF1 and its E3 ubiquitin ligase MIB1. To investigate the relationship between CEMIP and MIB1, we performed Co-IP assays and observed that CEMIP could interact with MIB1 both at the exogenous and endogenous level (Fig. 4A, B and Supplementary Fig. S6A, B). Moreover, as showed in Fig. 4C and Supplementary Fig. S6C, we indicated that CEMIP interacted with MIB1 though CEMIP-myc-∆4 (the 820–1204aa domain) but not CEMIP-myc-∆2 and -∆3 (the 295–819aa domain) which bound to GRAF1. Furthermore, the Duolink proximity ligation assays showed a direct linkage between CEMIP and MIB1 (Fig. 4D). Therefore, it suggested that CEMIP interacted with MIB1 though the 820–1204aa domain.

Notably, we observed that knock-down of CEMIP weakened the interaction of GRAF1 and MIB1, while exogenously expressed CEMIP enhanced the interaction of GRAF1 and MIB1, indicating that CEMIP promoted the interaction of GRAF1 and MIB1 (Fig. 4E and supplementary Fig. S6D). In addition, silence of CEMIP or MIB1 significantly attenuated the ubiquitination of GRAF1 (Fig. 4F). Together, these results demonstrated that CEMIP acted as a scaffold protein in bridging GRAF1 and MIB1, which was critical to GRAF1 degradation.

### CEMIP promotes metastasis of CRC cells by bridging GRAF1 and MIB1

**Fig. 5:**
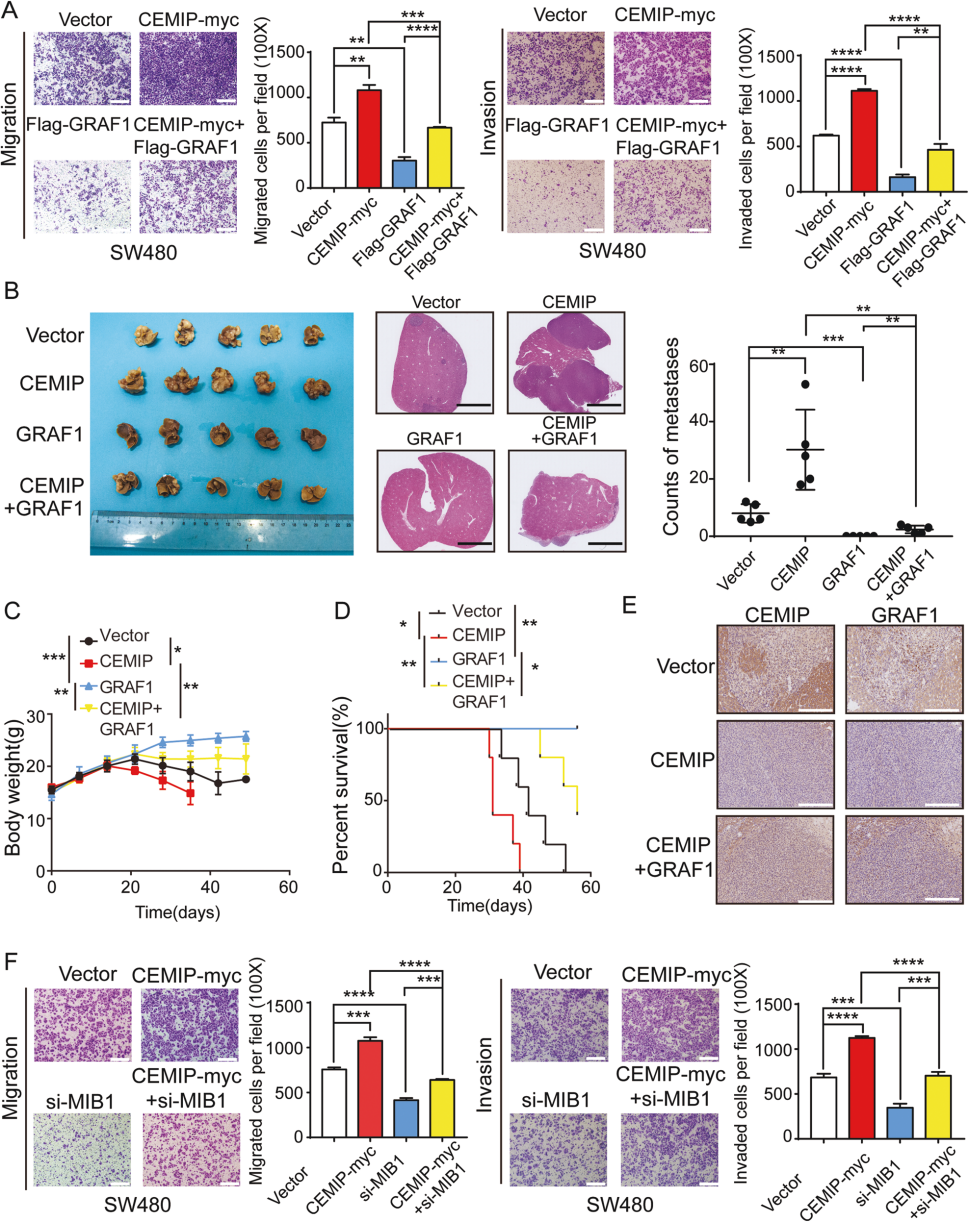
CEMIP promotes metastasis of CRC cells by bridging GRAF1 and MIB1. **A** Transwell chamber migration and invasion of SW480 cells transfected with the indicated constructs. **P < 0.01; ***P < 0.001; ****P < 0.0001. **B** Representative images of livers of BALB/c nude mice fixed in 10% formaldehyde. Numbers of liver metastases were detected in each group (day 28, *n* = 5). Representative HE staining of liver tissues was shown. Scale bar, 1 mm. **P < 0.01; ***P < 0.001. **C** Body weight curves of mice. *P < 0.05; **P < 0.01; ***P < 0.001. **D** Survival curves of mice. *P < 0.05; **P < 0.01. **E** Representative immunohistochemical staining for CEMIP, GRAF1 in primary colorectal cancer tissues of BALB/c nude mice mentioned above. The group of GRAF1 were not founded in metastatic foci. Scale bar, 50 μm. **F** Transwell chamber migration and invasion of SW480 cells transfected with the indicated constructs. ***P < 0.001; ****P < 0.0001.

Our previous data demonstrated that CEMIP promoted CRC metastasis. Here, we found that exogenously expressed GRAF1 attenuated CEMIP-mediated migration and invasion of CRC cells, while knock-down of GRAF1 promoted the migration and invasion of CRC cells which was inhibited by silence of CEMIP (Fig. 5A, Supplementary Fig. S7A). Moreover, to determine the relation of CEMIP and GRAF1 in vivo, we structured liver metastases model of BALB/c nude mice by intrasplenic injection. Compared to control group, it developed more liver metastases in CEMIP group, while less liver metastases in GRAF1 group. Importantly, liver metastases in CEMIP + GRAF1 group were significantly less than in CEMIP group, indicating that GRAF1 attenuated CEMIP-mediated liver metastasis (Fig. 5B). Consistently, it showed more weight loss and shorter survival of mice in CEMIP group compared with CEMIP + GRAF1 group, indicating that GRAF1 alleviated CEMIP-mediated weight loss and prolonged survival of mice (Fig. 5C, D). The IHC assays confirmed a negatively correlation between of CEMIP and GRAF1 (Fig. 5E). Furthermore, we observed that exogenously expressed CEMIP promoted the migration and invasion of CRC cells, which was weakened by knock-down of MIB1 (Fig. 5F). While suppression of MIB1 further depressed the migration and invasion of CRC cells which were inhibited by silencing CEMIP (Supplementary Fig. S7B). Summarily, we demonstrated that CEMIP promotes the metastasis of CRC cells by bridging GRAF1 and MIB1.

### CEMIP activates CDC42/MAPK pathway through inhibiting GRAF1

It was reported that GRAF1 regulated the expression of CDC42, which was able to activate the PAK1/MAPK pathway and led to the migration and invasion of cancer cells [28, 29]. Notably, our mRNA sequencing and KEGG (Kyoto Encyclopedia of Genes and Genomes) analyses showed a negative correlation between GRAF1 and the MAPK pathway (Supplementary Fig. S8A and Supplementary Table S4), and GESA (Gene Set Enrichment Analysis) analyses showed that CEMIP was positively associated with MAPK pathway (Supplementary Fig. S8B), suggesting that CEMIP promoted the migration and invasion of CRC cells though GRAF1/CDC42/PAK1/MAPK axis. In accordance with that, down-regulation of CEMIP resulted in the suppression of CDC42/PAK1/MAPK pathway but the increase of GRAF1, while up-regulation of CEMIP led to the hyper-activation of CDC42/PAK1/MAPK pathway but the decrease of GRAF1 (Supplementary Fig. S8C). Moreover, we observed that CEMIP-mediated activation of CDC42/PAK1/MAPK pathway was attenuated by silence of MIB1, and a similar synergistic effect of knock-down both of CEMIP and MIB1 to inhibit CDC42/PAK1/MAPK pathway (Supplementary Fig. S8D, E). Furthermore, in liver metastases model of BALB/c nude mice, IHC data showed that exogenously expressed GRAF1 attenuated CEMIP-mediated CDC42/MAPK pathway (Supplementary Fig. S8F). In addition, CDC42 inhibitor (ZCL278) blocked CEMIP-activated CDC42/PAK1/MAPK pathway (Supplementary Fig. S9A), and knock-down of CEMIP inhibited CDC42/PAK1/MAPK pathway, which was further suppressed by CDC42 inhibitor (Supplementary Fig. S9B). Therefore, these findings indicated that CEMIP promotes CDC42/MAPK pathway through inhibiting GRAF1.

### CEMIP promotes metastasis of CRC cells through GRAF1/CDC42/MAPK pathway-regulated EMT

**Fig. 6:**
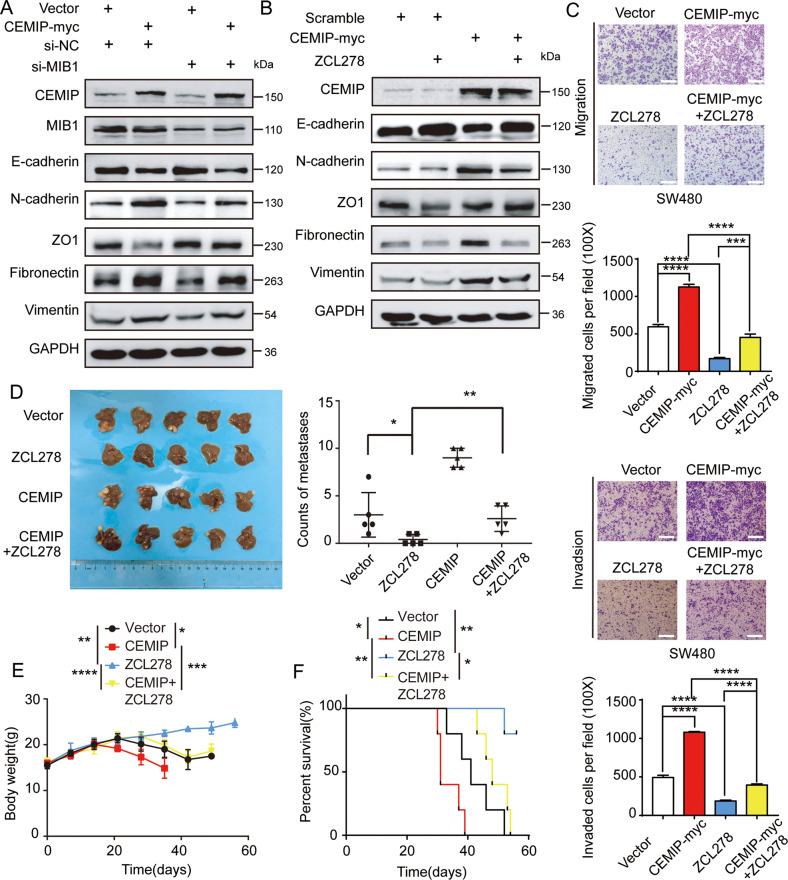
CEMIP promotes metastasis of CRC cells through GRAF1/CDC42/MAPK pathway-regulated EMT. **A** Western blotting analysis of EMT associated proteins in SW480 cells transfected with the indicated constructs. **B** Western blotting analysis of EMT associated proteins in SW480 cells transfected with the indicated constructs. **C** Transwell chamber migration and invasion of SW480 cells transfected with the indicated constructs with or without ZCL278. ***P < 0.001; ****P < 0.0001. **D** Representative images of livers of BALB/c nude mice, treated with or without ZCL278. Numbers of liver metastases were detected in each group (day 28, *n* = 5), mice were treated with or without ZCL278. *P < 0.05; **P < 0.01. **E** Body weight curves of mice treated with or without ZCL278. *P < 0.05; **P < 0.01; ***P < 0.001; ****P < 0.0001. **F** Survival curves of mice treated with or without ZCL278. *P < 0.05; **P < 0.01.

Considering we had proved that CEMIP activated CDC42/MAPK pathway, and it was reported that MAPK signaling was a key regulator of epithelial-mesenchymal transition (EMT) and tumor metastasis, we deduced that CEMIP promoted CRC metastasis via MAPK signaling-induced EMT [30, 31]. We found that CEMIP and MIB1 had a synergistic effect in the up-regulation of EMT in CRC cells (Fig. 6A, Supplementary Fig. S10A). Moreover, we showed that CDC42 inhibitor (ZCL278) could significantly weaken CEMIP-induced EMT (Fig. 6B), and knock-down of CEMIP inhibited EMT of CRC cells, which was further suppressed by CDC42 inhibitor (Supplementary Fig. S10B). It suggested that CEMIP promoted EMT of CRC cells through GRAF1/CDC42/MAPK pathway.

Then we validated the correlation of CEMIP-mediated CRC metastasis and CDC42/MAPK pathway-regulated EMT. Our results indicated that up-regulation of CEMIP increased the migration and invasion of CRC cells, which was attenuated by CDC42 inhibitor (Fig. 6C). Meanwhile, knock-down of CEMIP decreased the migration and invasion of CRC cells, which was further suppressed by CDC42 inhibitor (Supplementary Fig. S10C). Moreover, in liver metastases model of BALB/c nude mice, we observed that, compared to control group, it developed more liver metastases in CEMIP group, while less liver metastases in ZCL278 group. Importantly, liver metastases in CEMIP + ZCL278 group were significantly less than in CEMIP group (Fig. 6D and Supplementary Fig. S10D), indicating that CDC42 inhibitor suppressed CEMIP-mediated liver metastasis. Consistently, it showed less weight loss and better survival of mice in CEMIP + ZCL278 group compared with that in CEMIP group, indicating that CDC42 inhibitor alleviated CEMIP-mediated weight loss and prolonged survival of mice (Fig. 6E, F). In addition, IHC assays showed that CEMIP decreased GRAF1 expression but up-regulated CDC42/MAPK pathway, which was suppressed CDC42 inhibitor (Supplementary Fig. S10E). In conclusion, CEMIP promotes metastasis of CRC cells through GRAF1/CDC42/MAPK pathway-regulated EMT.

## Discussion

Metastasis is one of the main causes of death in patients with advanced colon cancer, and it is an obstacle in the clinical treatment. Our previous research proved that CEMIP functionally promoted CRC metastasis and was closely related to poor outcomes. And CDC42/MAPK pathway was proved to play a key role in the regulation of tumor growth, metastasis and drug resistance [28, 32, 33]. In the present study, we demonstrated that CEMIP promoted CRC metastasis through activating CDC42/MAPK pathway by enhancing the degradation of GRAF1, and CDC42 inhibitor suppressed CEMIP-mediated CRC metastasis.

Accumulating evidence showed that CEMIP was potential to serve as a therapeutic target for tumor metastasis. To further explore the underlying molecular mechanisms of CEMIP-mediated CRC metastasis, we identified CEMIP interacting with GRAF1, and CEMIP was negative correlated with GRAF1 in CRC. GRAF1 was reported to be a tumor suppressor that was genetically or functionally inactivated in several tumors, including gastric cancer, ovarian cancer, and metastatic brain cancer [19–22]. Interestingly, GRAF1 or CEMIP alone could not predict both of PFS and OS of CRC patients. However, we found that the combination of CEMIP and GRAF1 could effectively predict the survival of CRC patients. GRAF1 was comprised of a BAR domain, a PH domain, a RhoGAP domain, and a SH3 domain which was originally identified binding to focal adhesion kinase [23, 24]. Importantly, we elucidated that the domain (295–819aa sequence) of CEMIP was indispensable for interacting with the SH3 domain of GRAF1, which was essential to the ubiquitination and degradation of GRAF1. As a whole, we demonstrated that CEMIP interacted with GRAF1 to negatively regulate the stability of GRAF1.

Although CEMIP promoted the ubiquitination level and degradation of GRAF1, CEMIP had no canonical catalytic structure of E3 ubiquitin ligase. Hence, we hypothesized that CEMIP regulated the stability of GRAF1 by relying on an E3 ubiquitin ligase. Then we identified MIB1 as a potential E3 ubiquitin ligase of GRAF1. MIB1 was reported as an E3 ubiquitin ligase that promoted the ubiquitination and degradation of Notch ligands [34], and played an important role in growth, metastasis, and micro-environment of tumor [35–37]. In the current research, we proved that MIB1 interacted with the RhoGAP domain of GRAF1, and negatively regulated the ubiquitination of GRAF1, which supported that GRAF1 was a ubiquitinated substrate of MIB1 in CRC. It was reported that E3 ligases and their scaffold proteins could bind with numerous substrates via their multi-domain structures [34]. Interestingly, we uncovered that CEMIP interacted with MIB1 though the 820–1204aa domain, but not the 295–819aa domain which bound to GRAF1, indicating that CEMIP could bind GRAF1 and MIB1 in the meantime. Moreover, CEMIP promoted the interaction of GRAF1 and MIB1, which significantly enhanced the ubiquitination of GRAF1. Collectively, our results supported that CEMIP acted as a scaffold protein in bridging GRAF1 and MIB1, which was critical to GRAF1 degradation.

Previous studies indicated that GRAF1, as a GTPase activating protein, inhibited the function of CDC42 through GTP hydrolysis via protein-protein interaction [38, 39]. CDC42 was proved to regulate EMT and cancer metastasis [28, 32, 40], and activate PAK1 (a serine/threonine protein kinase) which was involved in various tumor signaling pathways including MAPK [41]. In our research, we found that CEMIP down-regulated the expression level of GRAF1, activated CDC42/MAPK pathway-regulated EMT, thus promoted the metastasis of CRC cells. It suggested that CEMIP activates CDC42/MAPK pathway-regulated EMT by enhancing the degradation of GRAF1, which is indispensable to CEMIP-mediated migration and invasion of CRC cells. Importantly, we demonstrated that CDC42 inhibitor dramatically suppressed CEMIP-mediated CRC metastasis in vitro *and* in vivo. Altogether, our results revealed that CEMIP promoted CRC metastasis through GRAF1/CDC42/MAPK pathway-regulated EMT.

In conclusion, we identified CEMIP interacting with GRAF1, and the combination of high-CEMIP and low-GRAF1 predicted poor survival of patients. Moreover, we uncovered that CEMIP acted as a scaffold protein in bridging MIB1 and GRAF1, which was critical to GRAF1 degradation and CEMIP-mediated CRC metastasis. Furthermore, we found that CEMIP activated CDC42/MAPK pathway-regulated EMT by enhancing the degradation of GRAF1, which was indispensable to CEMIP-mediated migration and invasion of CRC cells. Importantly, we proved that CDC42 inhibitor suppressed CEMIP-mediated CRC metastasis in vitro and in vivo. Therefore, our research suggested that CDC42 inhibitor could be a novel therapeutic strategy for CEMIP-mediated CRC metastasis.

## Methods and materials

### Cell culture

Human CRC cells (HCT116, SW480) and HEK293T cells were purchased from Cell Bank, Type Culture Collection, Chinese Academy of Sciences (CBTCCCAS, Shanghai, China), and were cultured in DMEM or RPMI 1640 (GIBCO, USA) supplemented with 10% fetal bovine serum (BI, Israel), 100 U/mL penicillin and 100 μg/mL streptomycin. All human cell lines have been authenticated using STR profiling within the last 3 years. All experiments were performed with mycoplasma-free cells.

### Immunoprecipitation and Western blot analysis

For immunoprecipitation, cells were harvested and resuspended in NETN buffer (20 mM of Tris HCl, pH 8.0, 100 mM of NaCl, 1 mM of EDTA, and 0.5% NonidetP-40). Cell lysate was centrifuged for 20 min at 13200 rpm at 4 °C. The supernatant was incubated with Pierce Protein G agarose beads (Santa Cruz, USA) which had been incubated with the primary antibody or IgG, shaking overnight at 4 °C. Then the precipitates were washed five times with NETN buffer and analyzed by NanoLC-Ultra 1D plus system mass spectrometer (Eksigent, USA), and analyzed by the Chemiluminescent Western Blot Detection Kit (Cat No. 32209, Thermo Fisher Scientific, USA).

### Transwell migration and invasion assays

For transwell assays, cells (5*10^4^ per well for migration, 10*10^4^ per well for invasion) were seeded in the upper well of the transwell chamber (Corning, USA) coated with or without Matrigel (BD Bioscience, USA) with serum-free medium and allowed to migrate or invade towards the medium containing 10% FBS in the lower compartment for 24 h or 48 h. Cells reaching the lower surface of each chamber were fixed with 4% paraformaldehyde for 20 min, stained with 0.1% crystal violet for 30 min and counted in five randomly selected microscopic fields.

### Proximity ligation assay (PLA)

The HCT116 cells were fixed by the blocking solution following the manufacture’s protocol (Duolink in situ fluorescence; Sigma, USA). Then, the primary antibodies, GRAF1 (Proteintech, #17747-1-AP, 1:50 dilution) and CEMIP (Santa Cruz, #sc-293483,1:25 dilution), GRAF1 (Proteintech, #17747-1-AP, 1:50 dilution) and MIB1 (Santa Cruz, #sc-393551,1:25 dilution), CEMIP (Proteintech, #21129-1-AP, 1:50 dilution) and MIB1 (Santa Cruz, #sc-393551, 1:25 dilution) or IgG (Rabbit, Cell Signaling Technology, #3900, 1:5000 dilution) and IgG (Mouse, Cell Signaling Technology, #5415, 1:5000 dilution) were incubated with the cells for 2 h at 37 °C. Then, cells were washed with wash buffer and incubated with PLA probe for 1 h at 37 °C. The ligation-ligase was added to cells at 37 °C for half an hour, cells were next incubated with amplification-polymerase solution for 100 min. The Duolink In Situ Mounting Medium with DAPI was applied to take photos under the confocal microscope.

### Animal experiments

All animal experiments were approved by the Medical Ethics Committee of Tongji Medical College, Huazhong University of Science and Technology. We established stable HCT116 lines which respectively overexpressed CEMIP, GRAF1 and CEMIP + GRAF1. Female BALB/c nude mice (4–6 weeks old) were allocated randomly into four groups (*n* = 10). Intrasplenic injection with 3*10^6^ HCT116 cells (Wild type, CEMIP, GRAF1 and CEMIP + GRAF1) was conducted in mice. Three days after cells inoculation, mice were treated with ZCL278(50 mg/kg) or DMSO every 2 days by intraperitoneal injection. Four weeks later, five randomly selected mice from each group were sacrificed, and liver metastasis was analyzed. The rest five mice in each group were observed for other 4 weeks for survival analysis. Mice were excluded if died from other causes (such as fighting and infection). Researchers were not blinded to the group allocation during the experiment and outcome assessment.

The other Methods and materials used in this research are described in Supplementary Methods and Materials.

## Supplementary information

original WB

Supplementary Figure S1–10 and Table S1–3

Supplementary Table.S4

Supplementary methods and materials

E3 ligase from ubibrowser database

STR report

Animal Ethics Provement

Ethics Provement

Supplementary sequencing and other data

check list

## Data Availability

The data that support the findings of this study are available from the corresponding author (taozhangxh@hust.edu.cn, zhangdejun@hust.edu.cn and yudandan@hust.edu.cn) upon reasonable request. Additional data are available as supplementary material.
