# Supplementary material for: CEMIP, acting as a scaffold protein for bridging GRAF1 and MIB1, promotes colorectal cancer metastasis via activating CDC42/MAPK pathway
Source: Cell Death Dis. 2023 Feb 27;14(2):167. doi: 10.1038/s41419-023-05644-z (PMC9971195; doi:10.1038/s41419-023-05644-z)

Figure 1

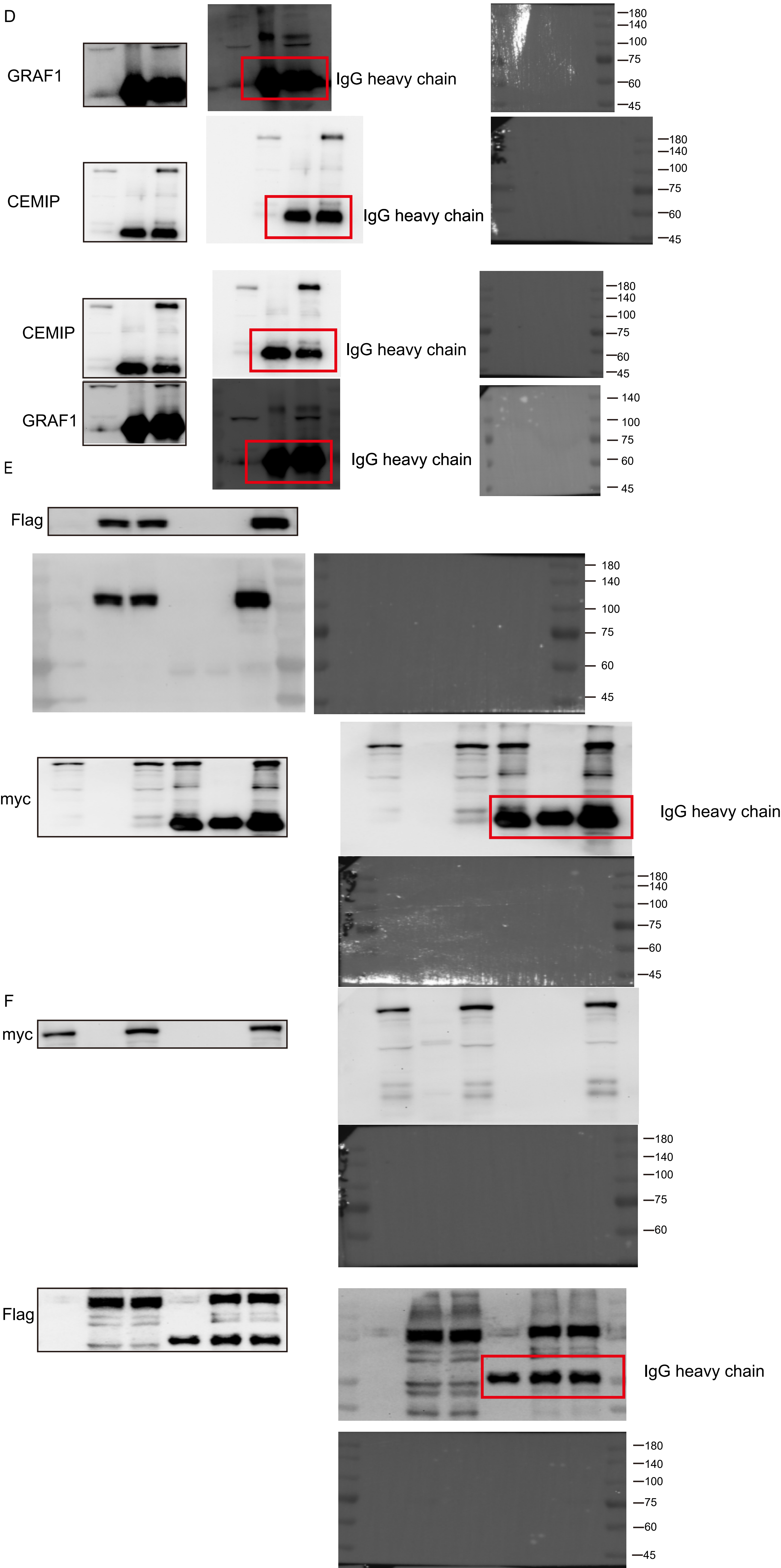
D
GRAF1
IgG heavy chain
180
140
100
75
60
45
CEMIP
IgG heavy chain
CEMIP
IgG heavy chain
GRAF1
IgG heavy chain
E
Flag
myc
IgG heavy chain
F
myc
Flag
IgG heavy chain

Figure 2

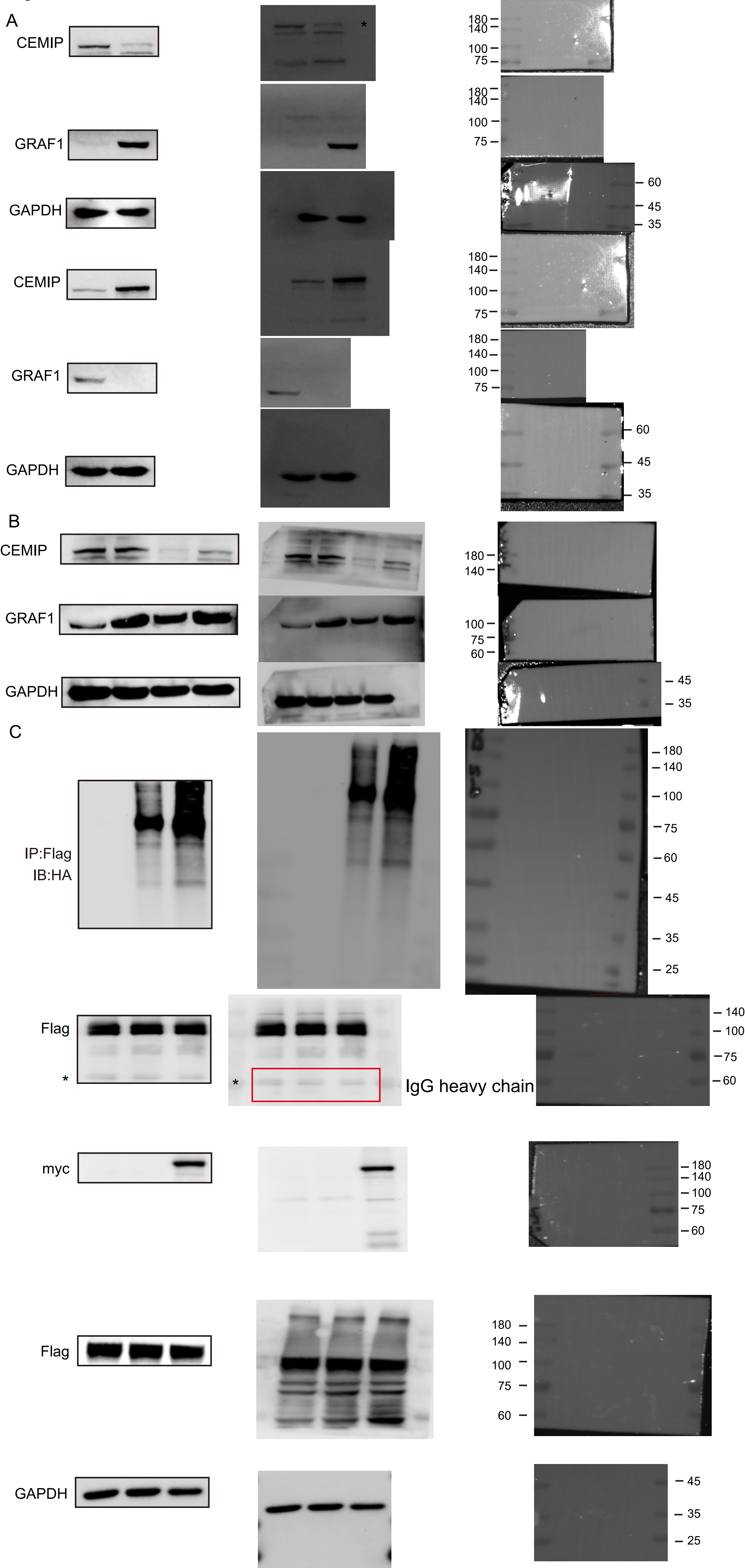

A
CEMIP
GRAF1
GAPDH
CEMIP
GRAF1
GAPDH
*
180
140
100
75
60
45
35
B
CEMIP
GRAF1
GAPDH
180
140
100
75
60
45
35
C
IP:Flag
IB:HA
180
140
100
75
60
45
35
25
Flag
*
IgG heavy chain
140
100
75
60
myc
180
140
100
75
60
Flag
180
140
100
75
60
GAPDH
45
35
25

Figure 2

D

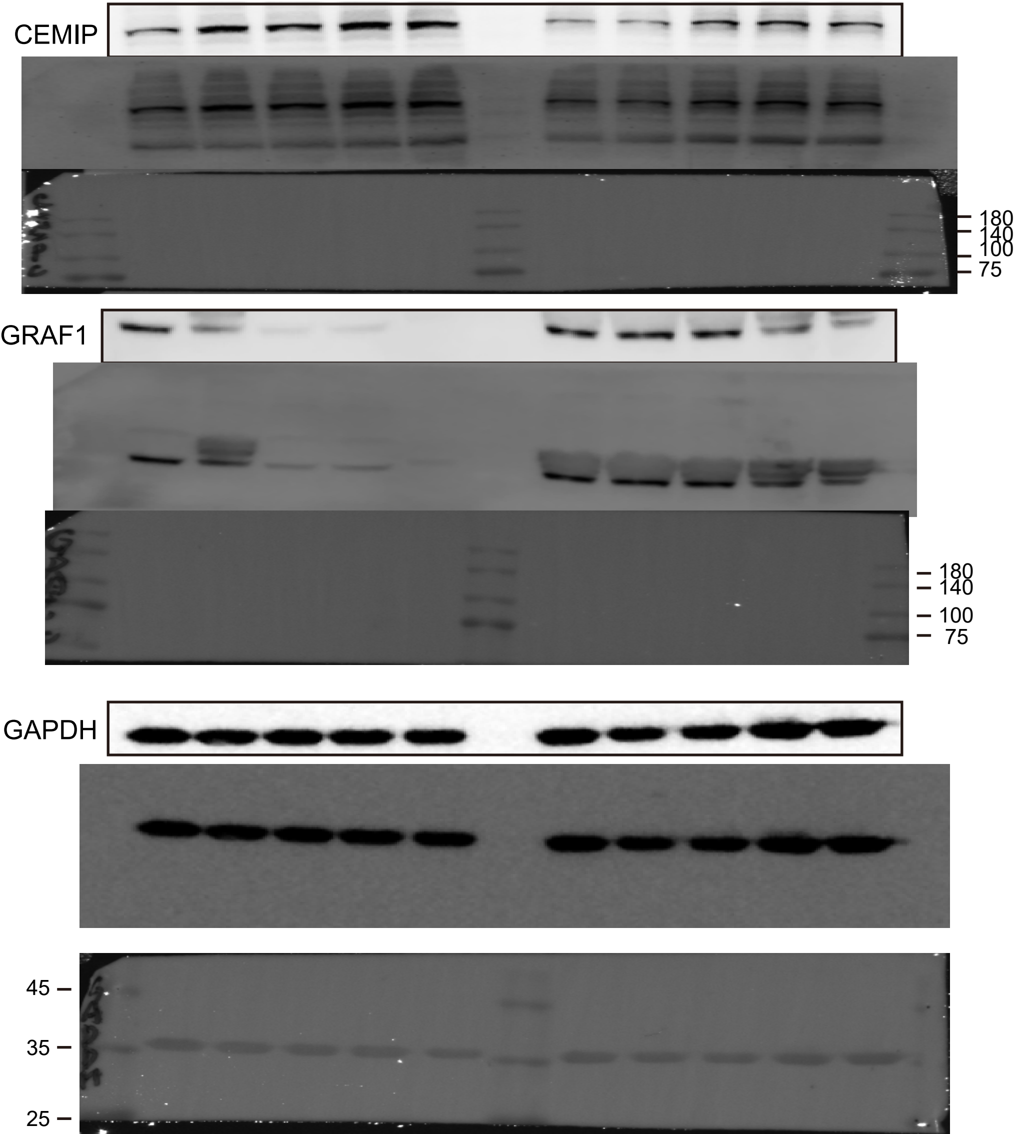

Figure 2

F

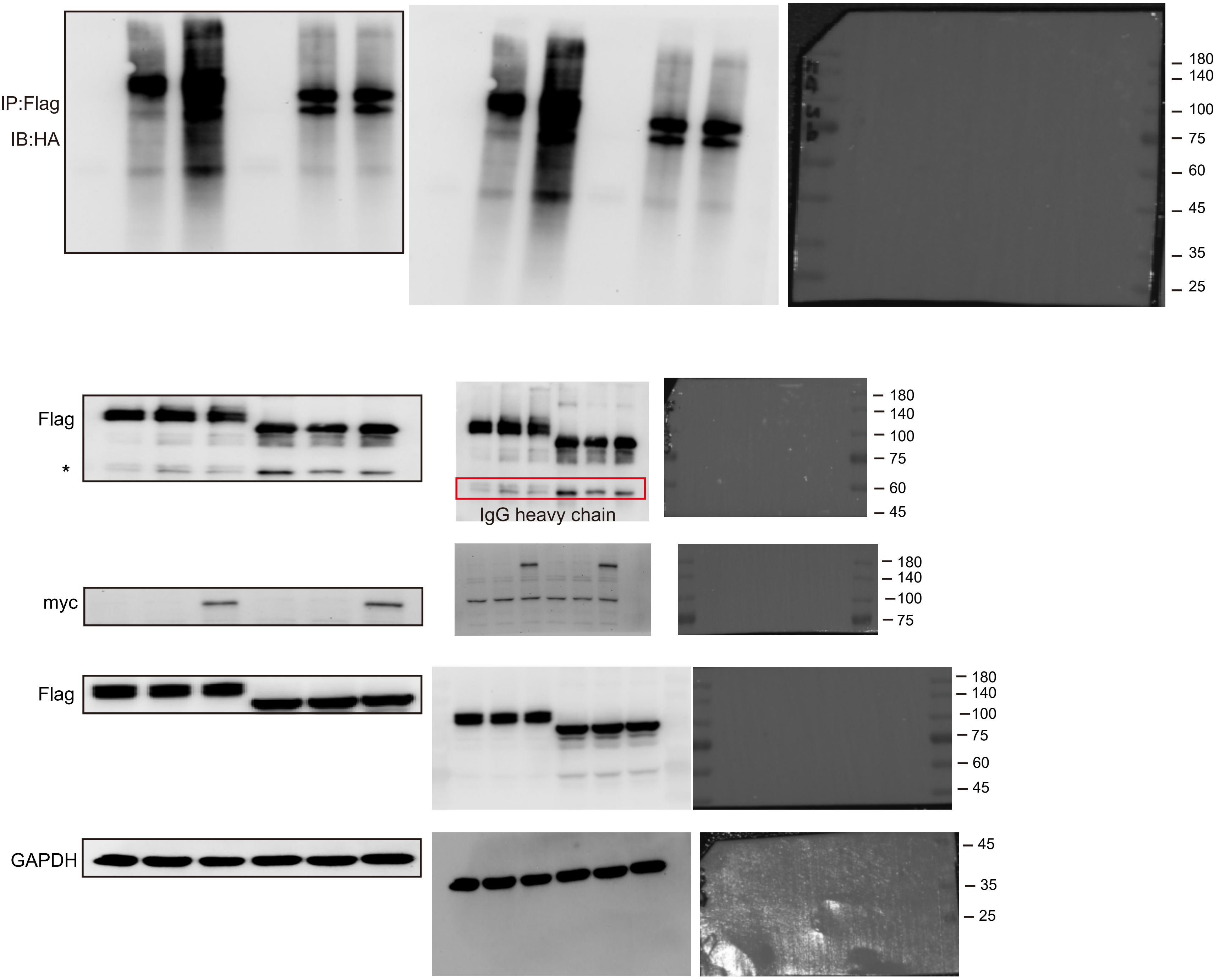
IP:Flag
IB:HA
180
140
100
75
60
45
35
25
Flag
*
IgG heavy chain
180
140
100
75
60
45
myc
180
140
100
75
Flag
180
140
100
75
60
45
GAPDH
45
35
25

G

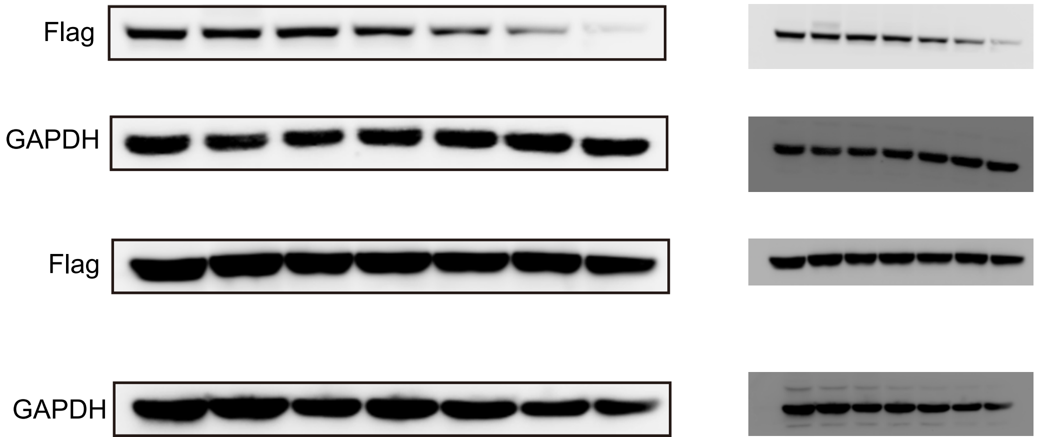
Flag
GAPDH
Flag
GAPDH

Figure 3

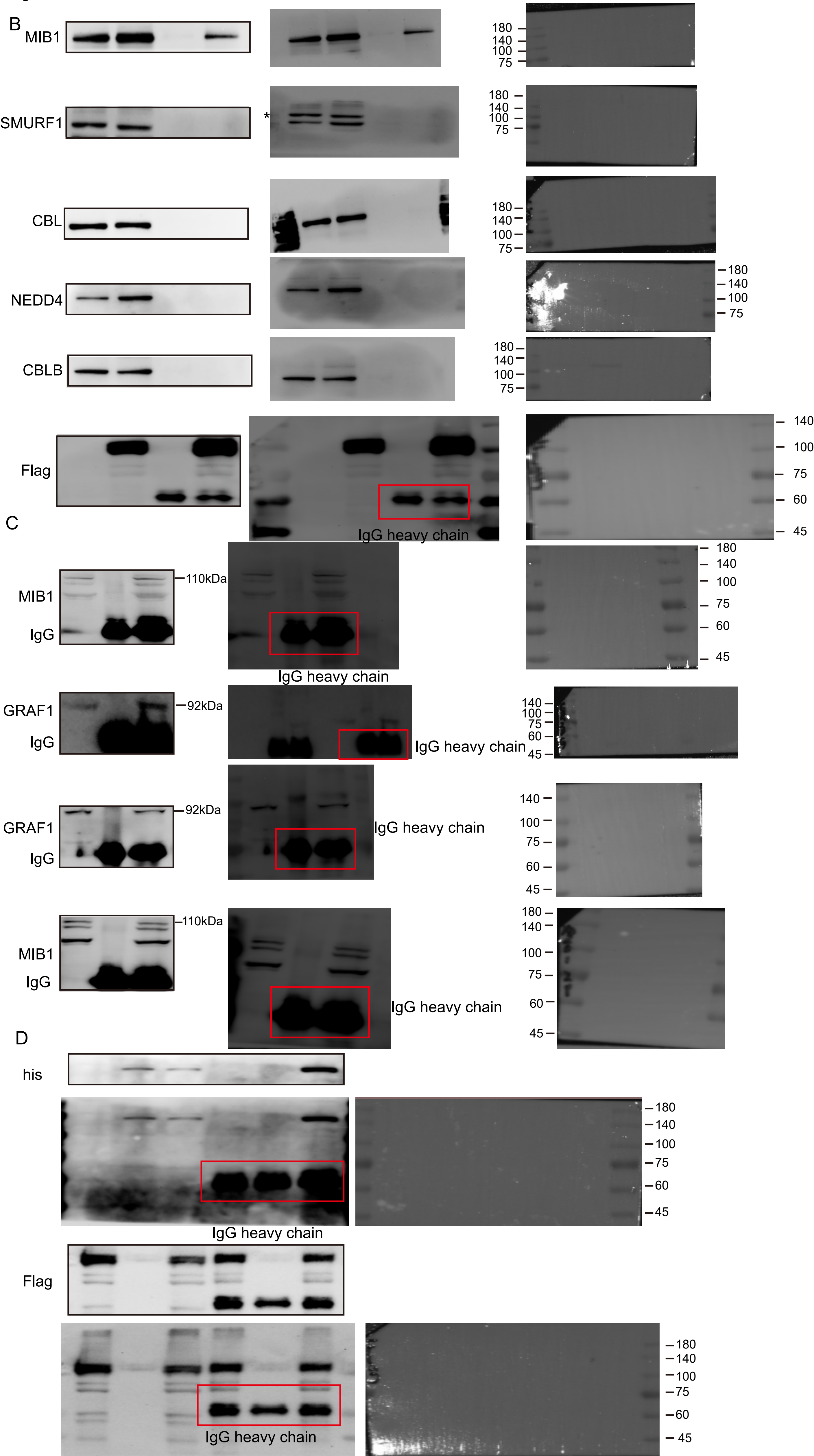

B
MIB1
SMURF1
*
CBL
NEDD4
CBLB
Flag
180
140
100
75
60
45
IgG heavy chain
C
MIB1
IgG
110kDa
GRAF1
92kDa
D
his
Flag

Figure 3

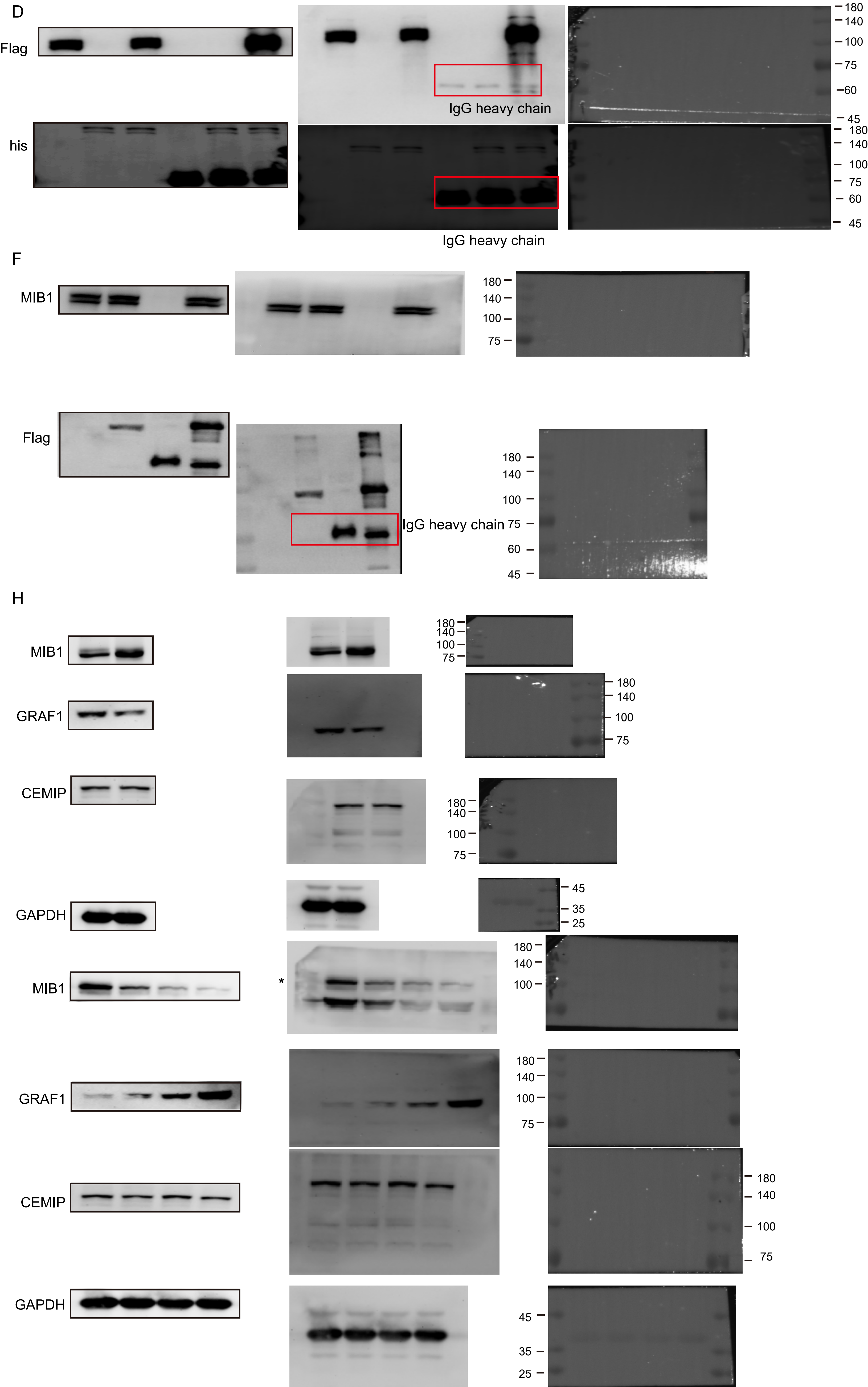

D
Flag
his
IgG heavy chain
IgG heavy chain
180
140
100
75
60
45
F
MIB1
Flag
IgG heavy chain
H
MIB1
GRAF1
CEMIP
GAPDH
MIB1
GRAF1
CEMIP
GAPDH
*
35
25

Figure 3

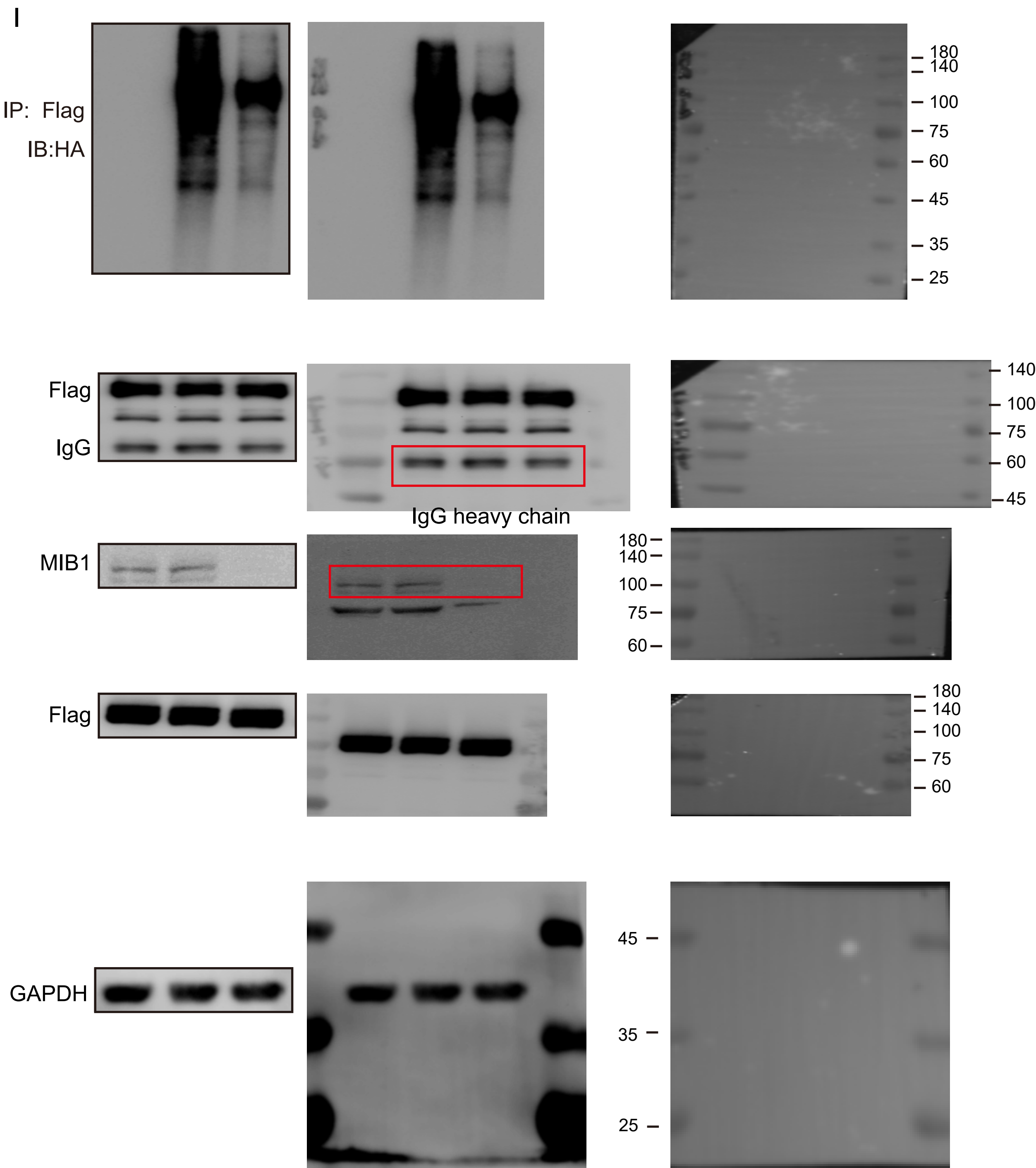
I
IP: Flag
IB:HA
180
140
100
75
60
45
35
25
Flag
IgG
IgG heavy chain
MIB1
Flag
GAPDH

Figure 4

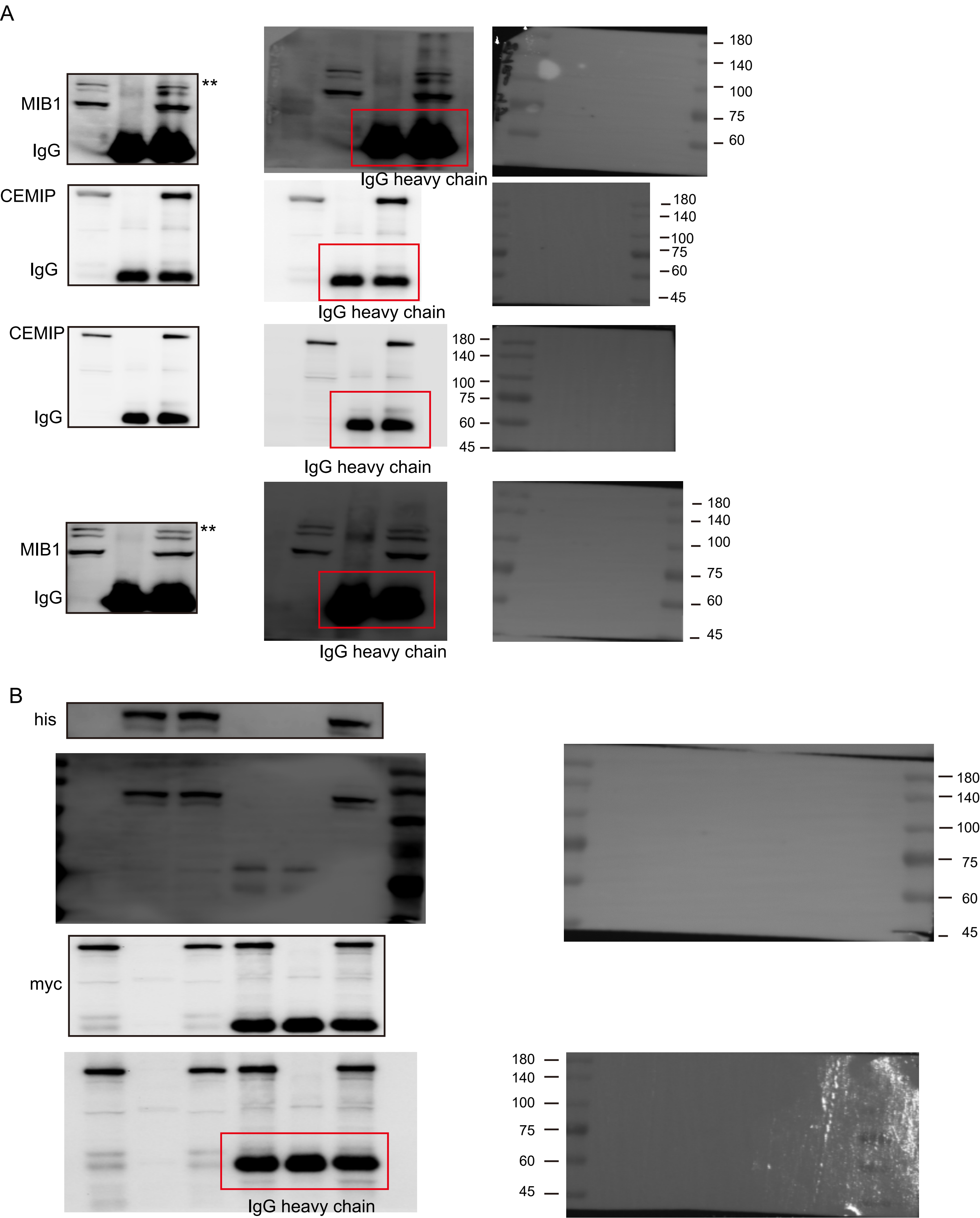
A
MIB1
IgG
**
IgG heavy chain
180
140
100
75
60
CEMIP
IgG
IgG heavy chain
180
140
100
75
60
45
CEMIP
IgG
IgG heavy chain
MIB1
IgG
**
IgG heavy chain
B
his
myc
IgG heavy chain

Figure 4

C

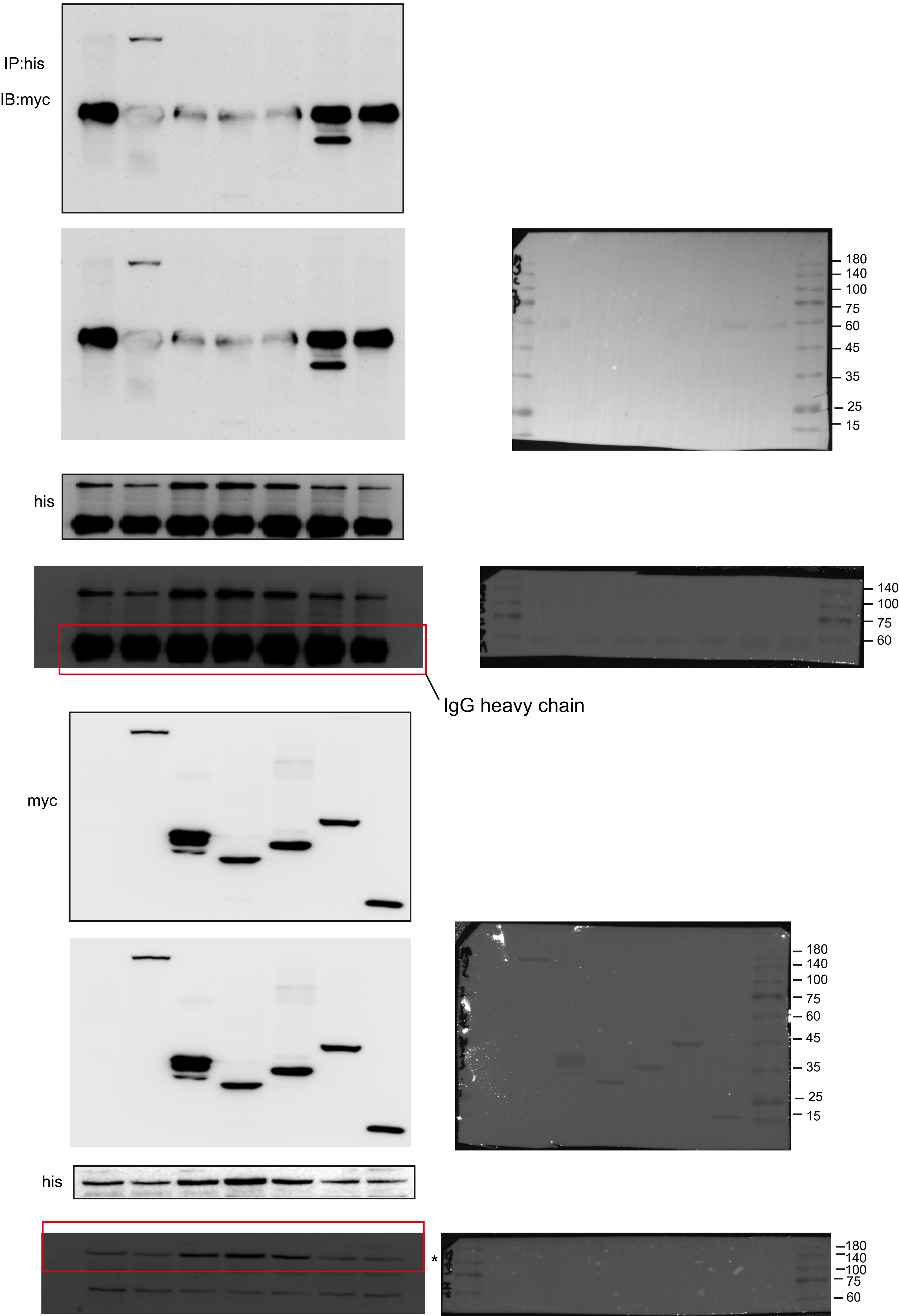

IP:his
IB:myc
180
140
100
75
60
45
35
25
15
his
140
100
75
60
IgG heavy chain
myc
180
140
100
75
60
45
35
25
15
his
*
180
140
100
75
60

Figure 4

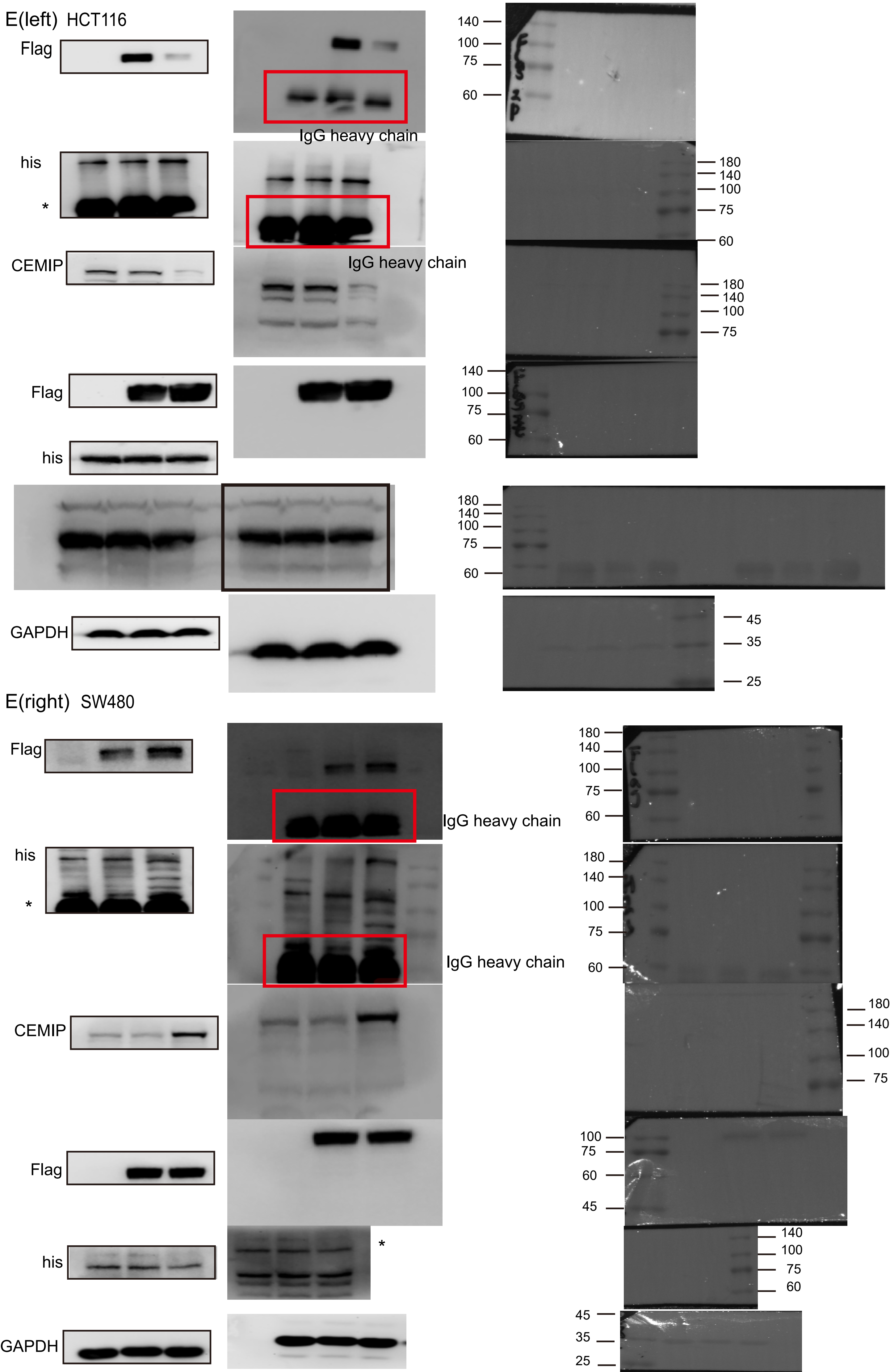

E(left) HCT116
Flag
his
*
CEMIP
Flag
his
GAPDH
IgG heavy chain
IgG heavy chain
140
100
75
60
180
140
100
75
60
180
140
100
75
140
100
75
60
180
140
100
75
60
45
35
25
E(right) SW480
Flag
his
*
CEMIP
Flag
his
*
GAPDH
IgG heavy chain
IgG heavy chain
180
140
100
75
60
180
140
100
75
60
180
140
100
75
100
75
60
45
140
100
75
60
45
35
25

Figure 4

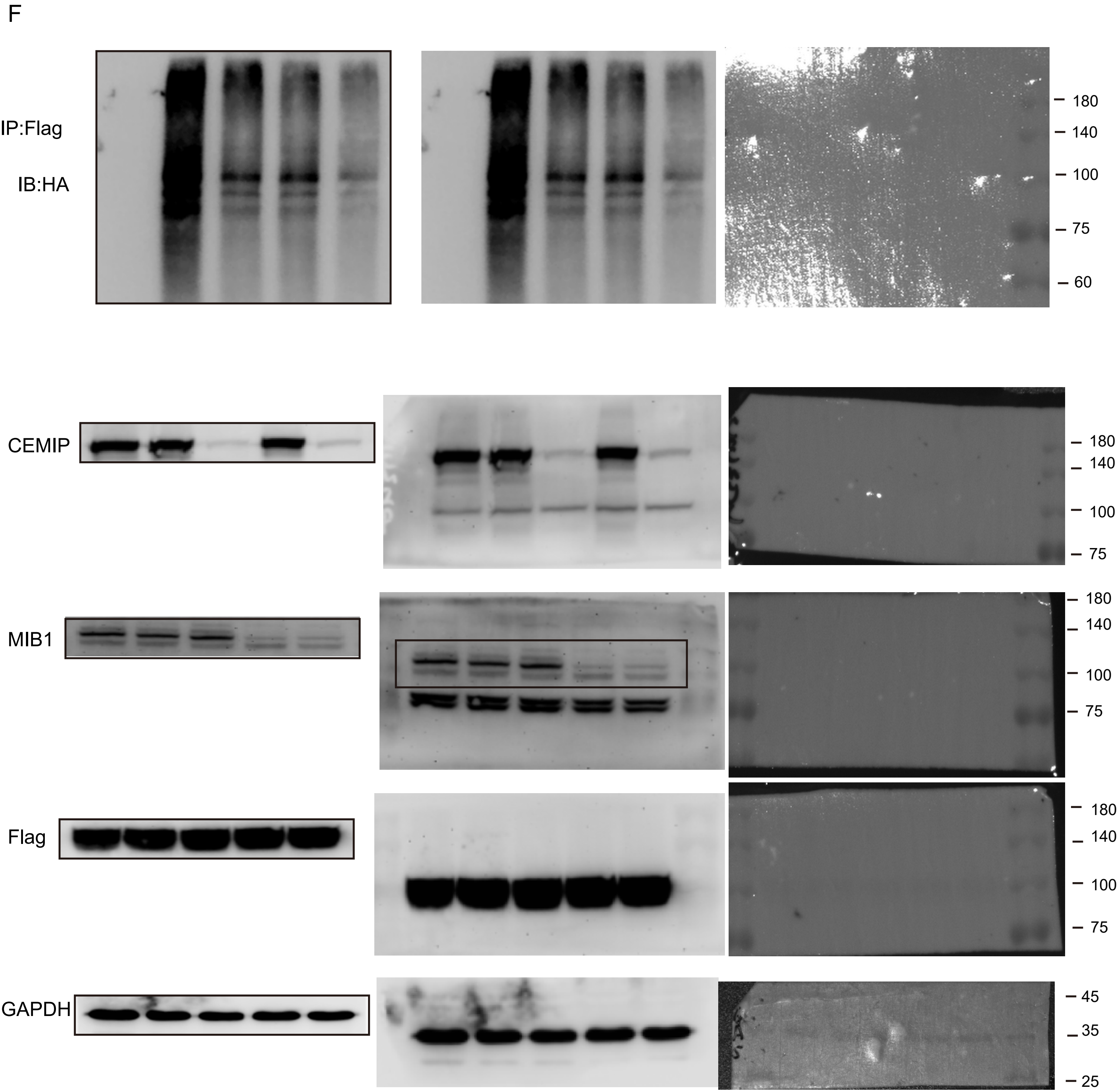
F
IP:Flag
IB:HA
180
140
100
75
60
CEMIP
180
140
100
75
MIB1
180
140
100
75
Flag
180
140
100
75
GAPDH
45
35
25

Figure 6

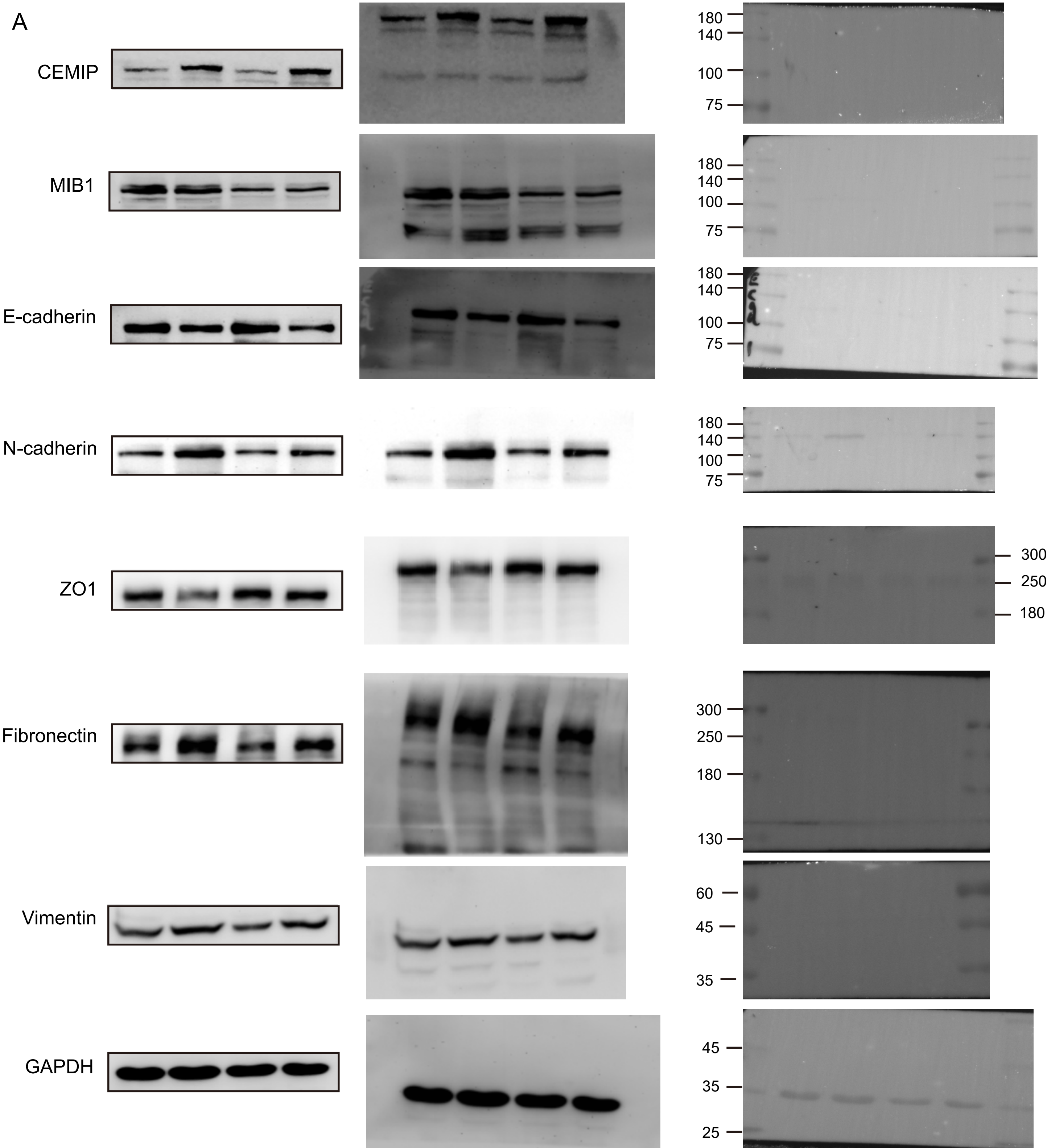

A
CEMIP
MIB1
E-cadherin
N-cadherin
ZO1
Fibronectin
Vimentin
GAPDH
180
140
100
75
180
140
100
75
180
140
100
75
180
140
100
75
300
250
180
300
250
180
130
60
45
35
45
35
25

Figure 6

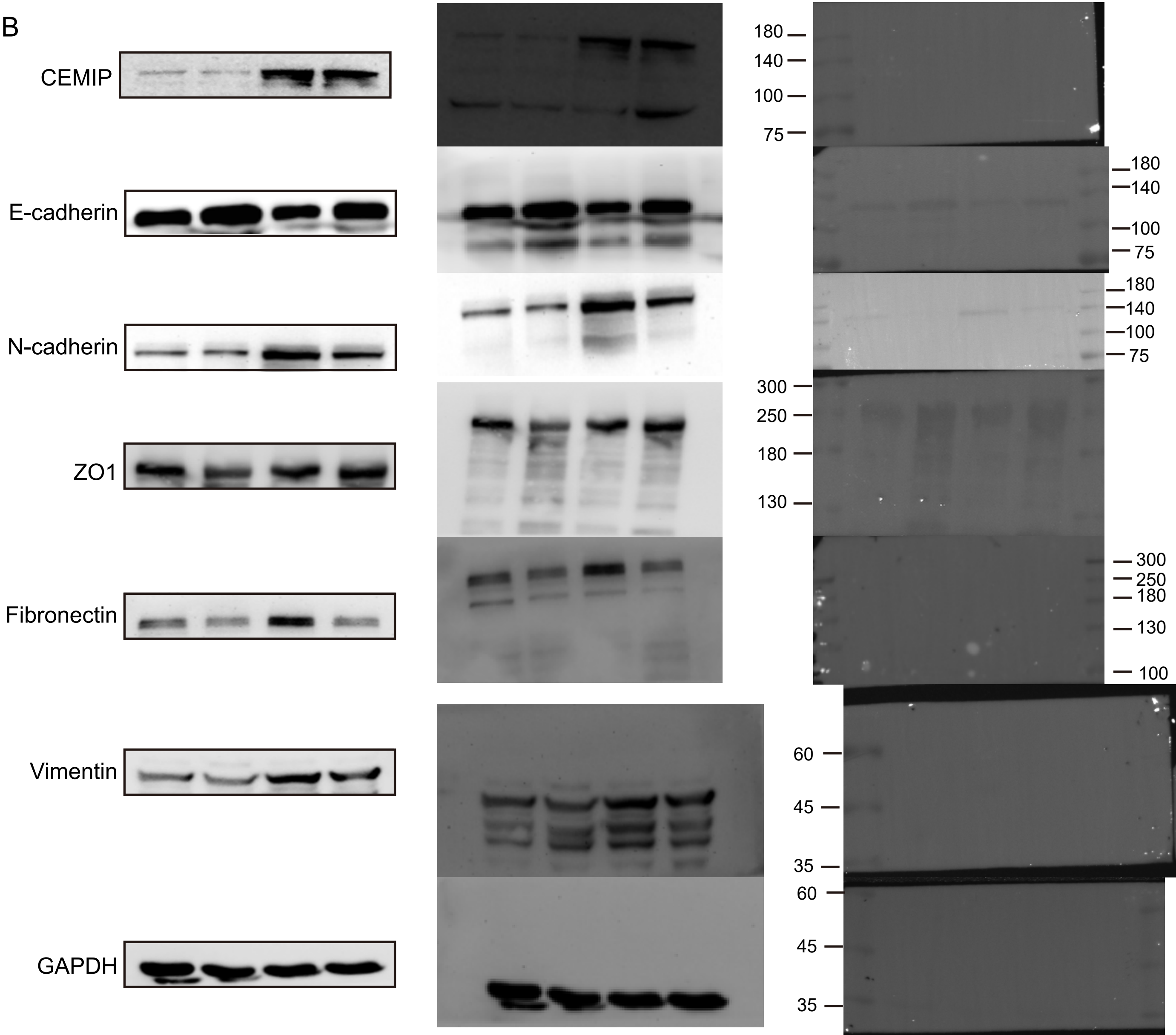
B
CEMIP
E-cadherin
N-cadherin
ZO1
Fibronectin
Vimentin
GAPDH
180
140
100
75
180
140
100
75
180
140
100
75
300
250
180
130
300
250
180
130
100
60
45
35
60
45
35

Supplementary Figure 2

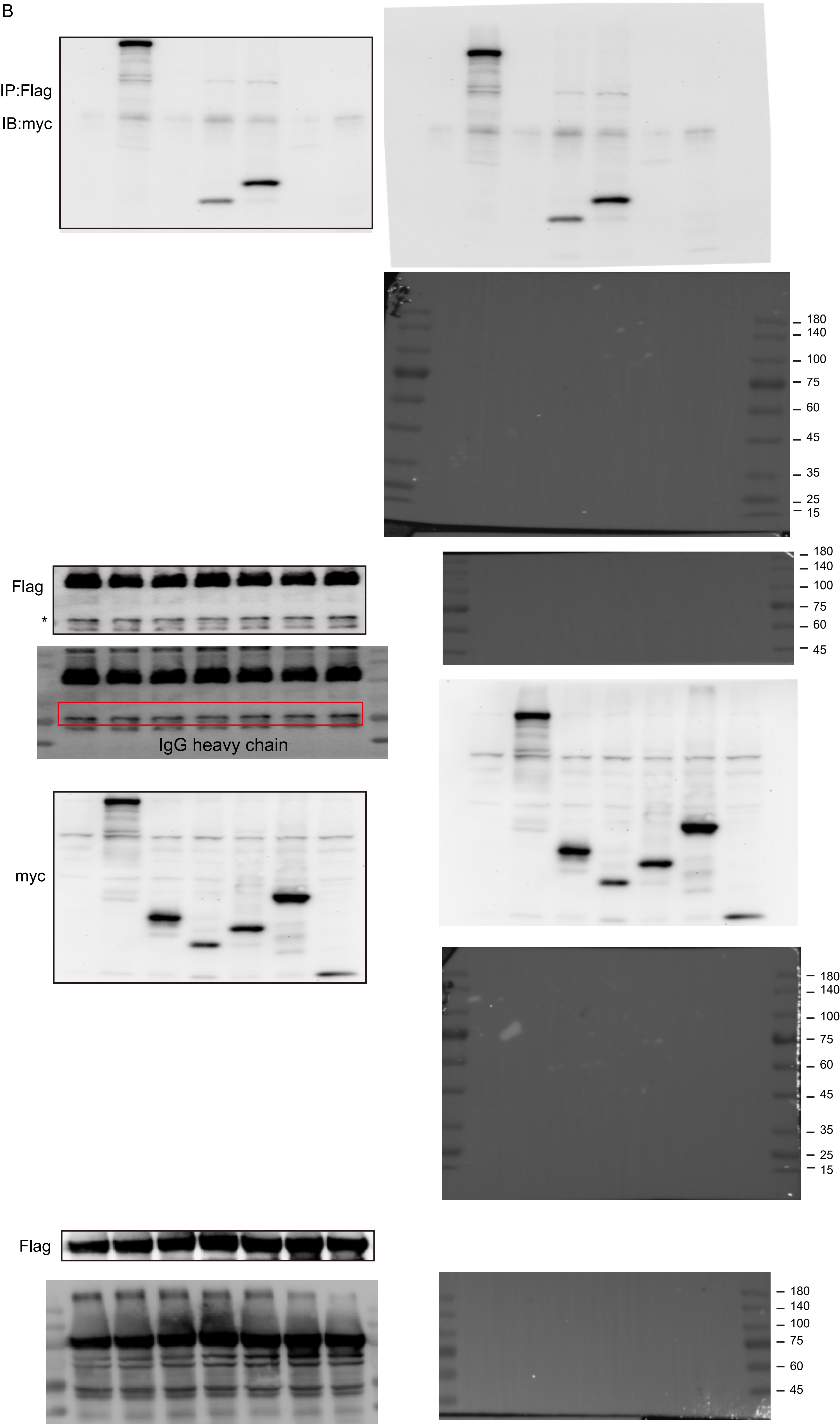

Supplementary Figure 2

C

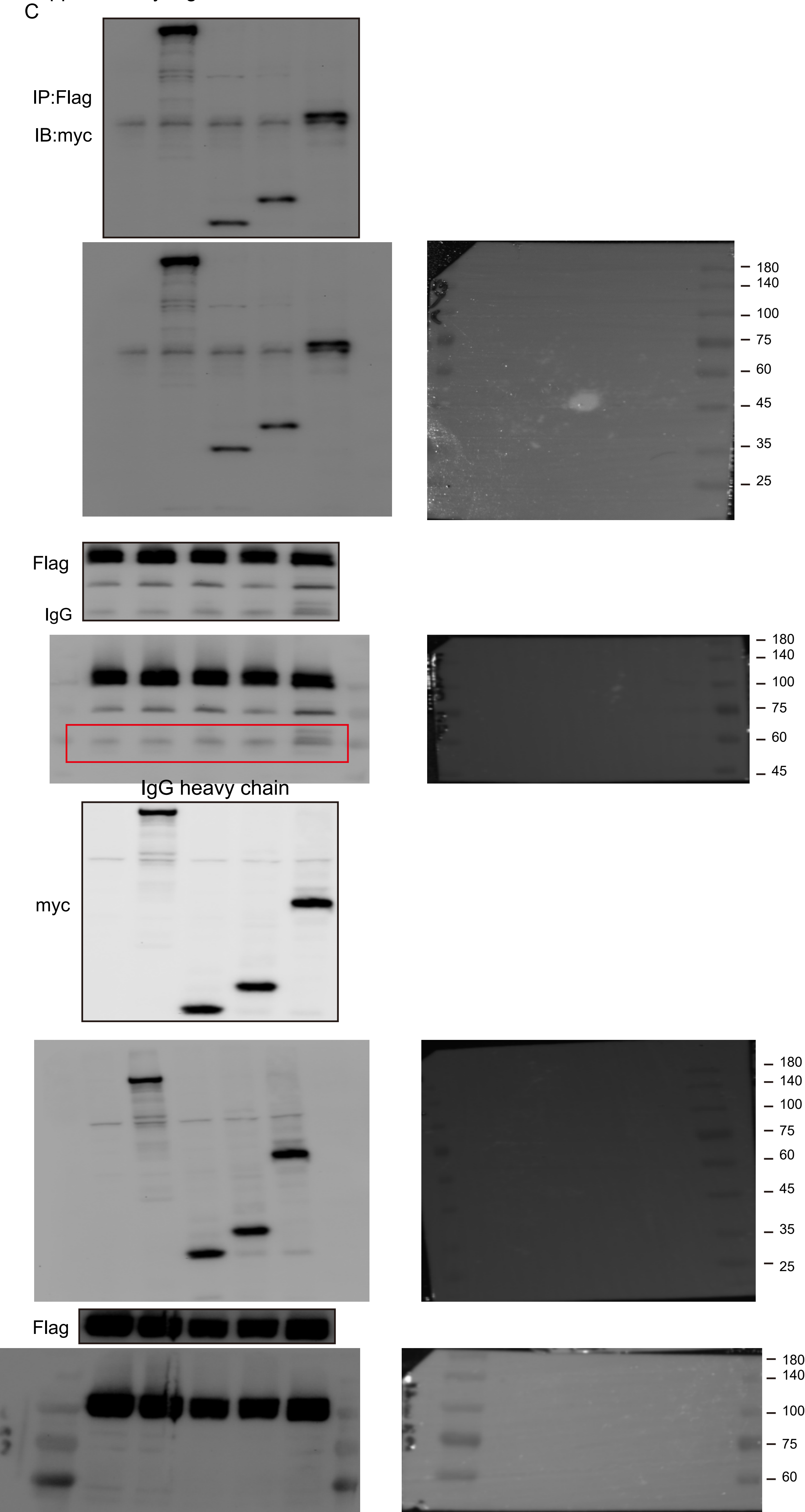

Supplementary Figure 3

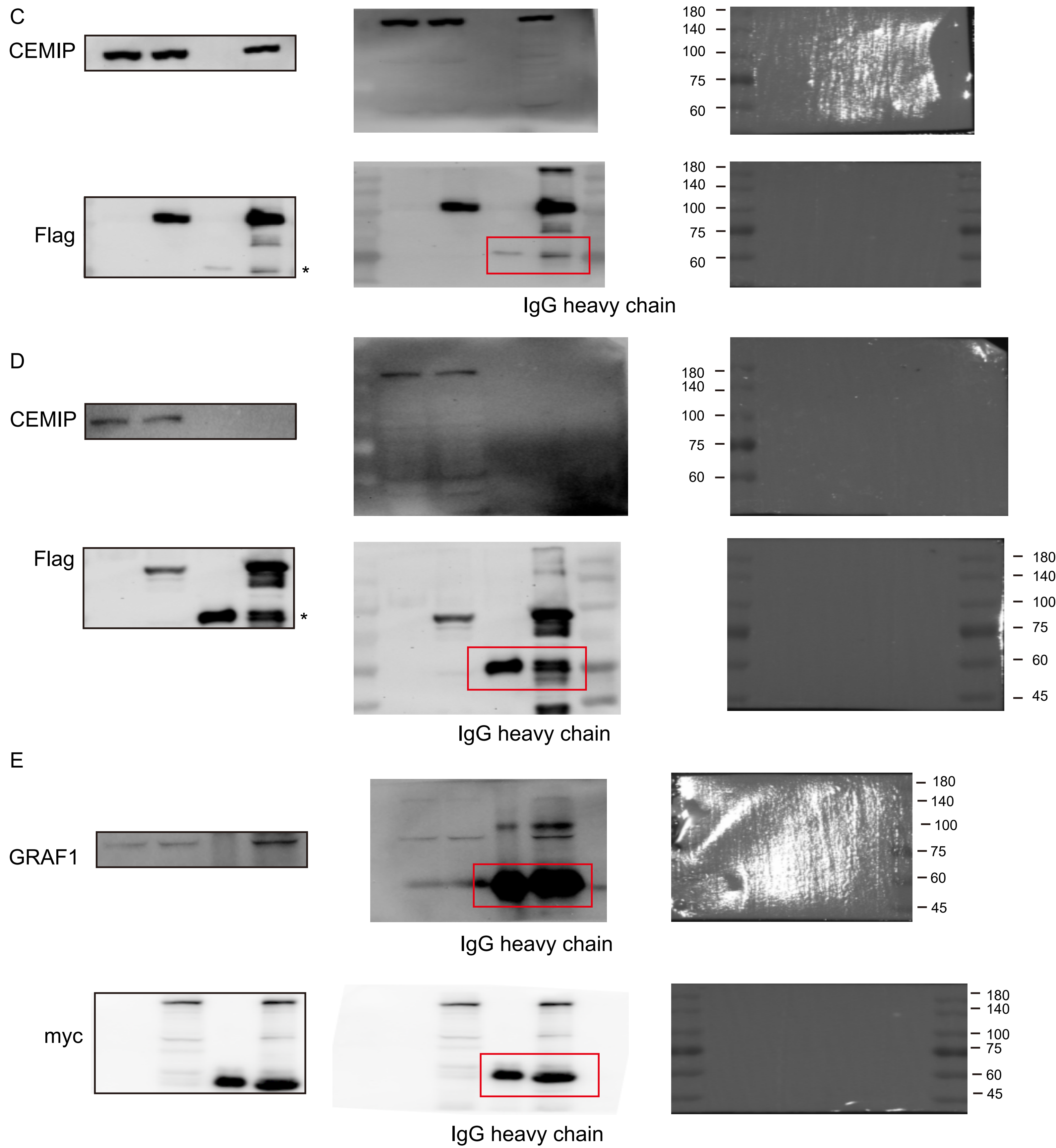

Supplementary Figure 5

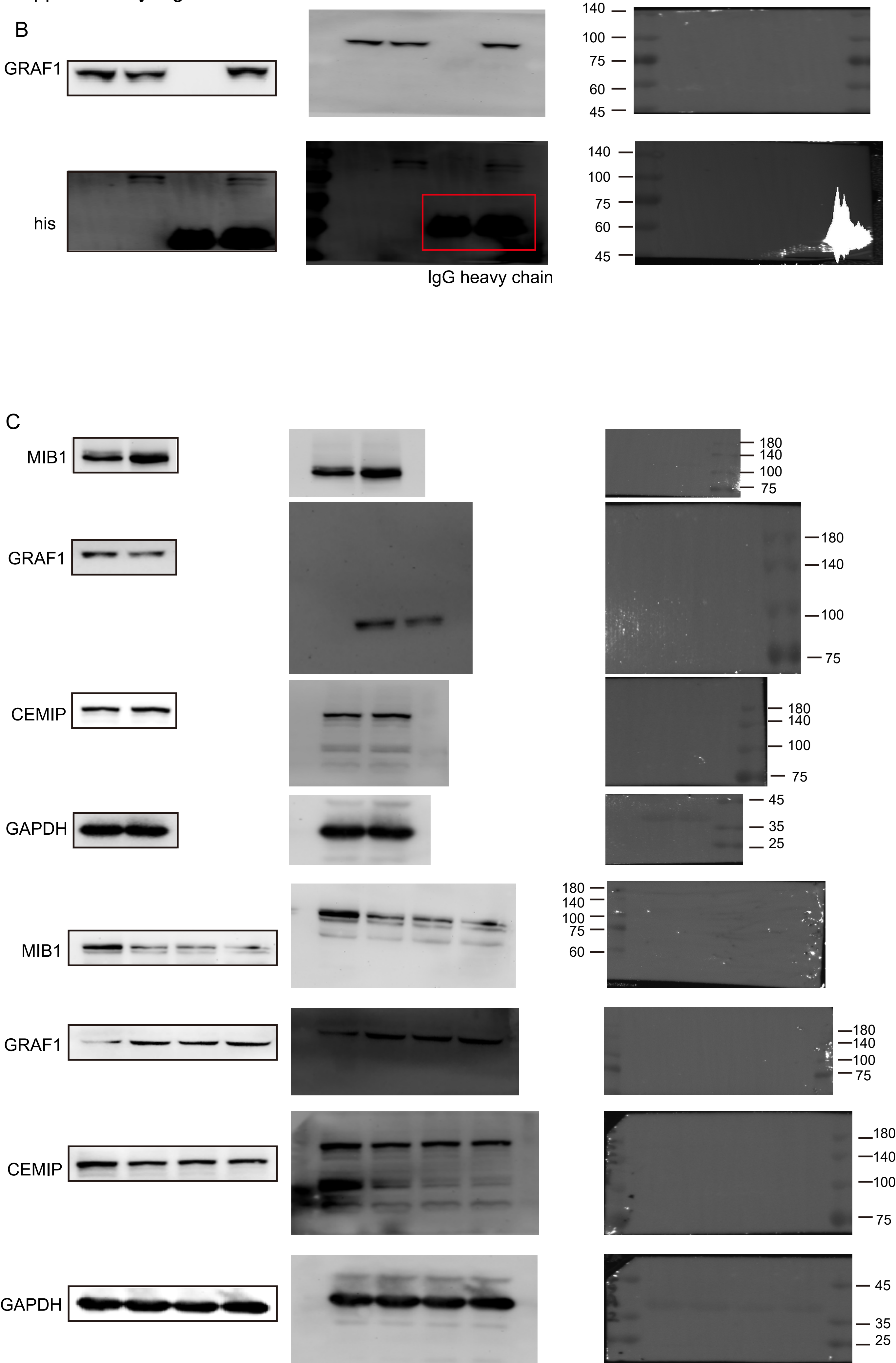

Supplementary Figure 6

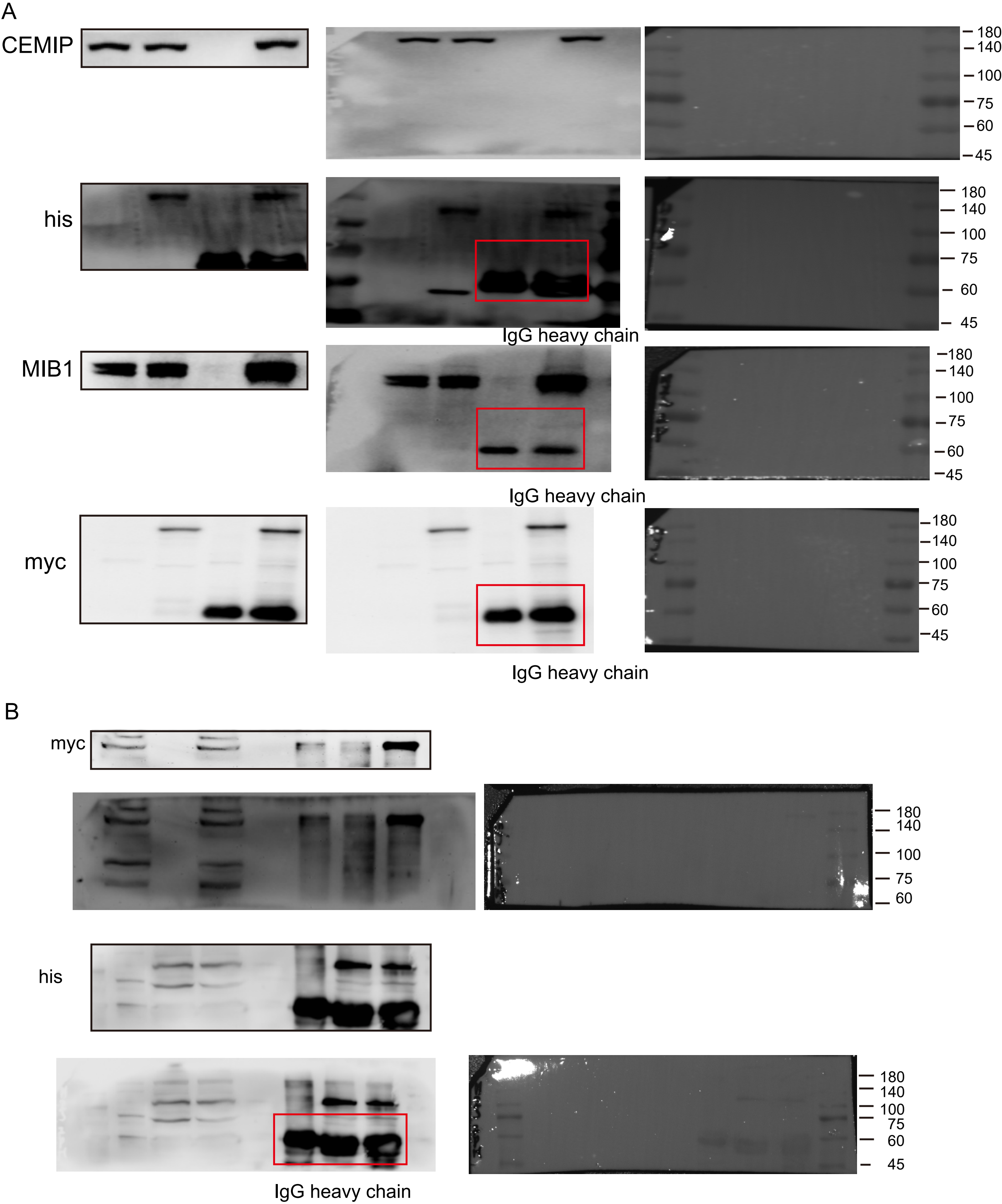

Supplementary Figure 6

C

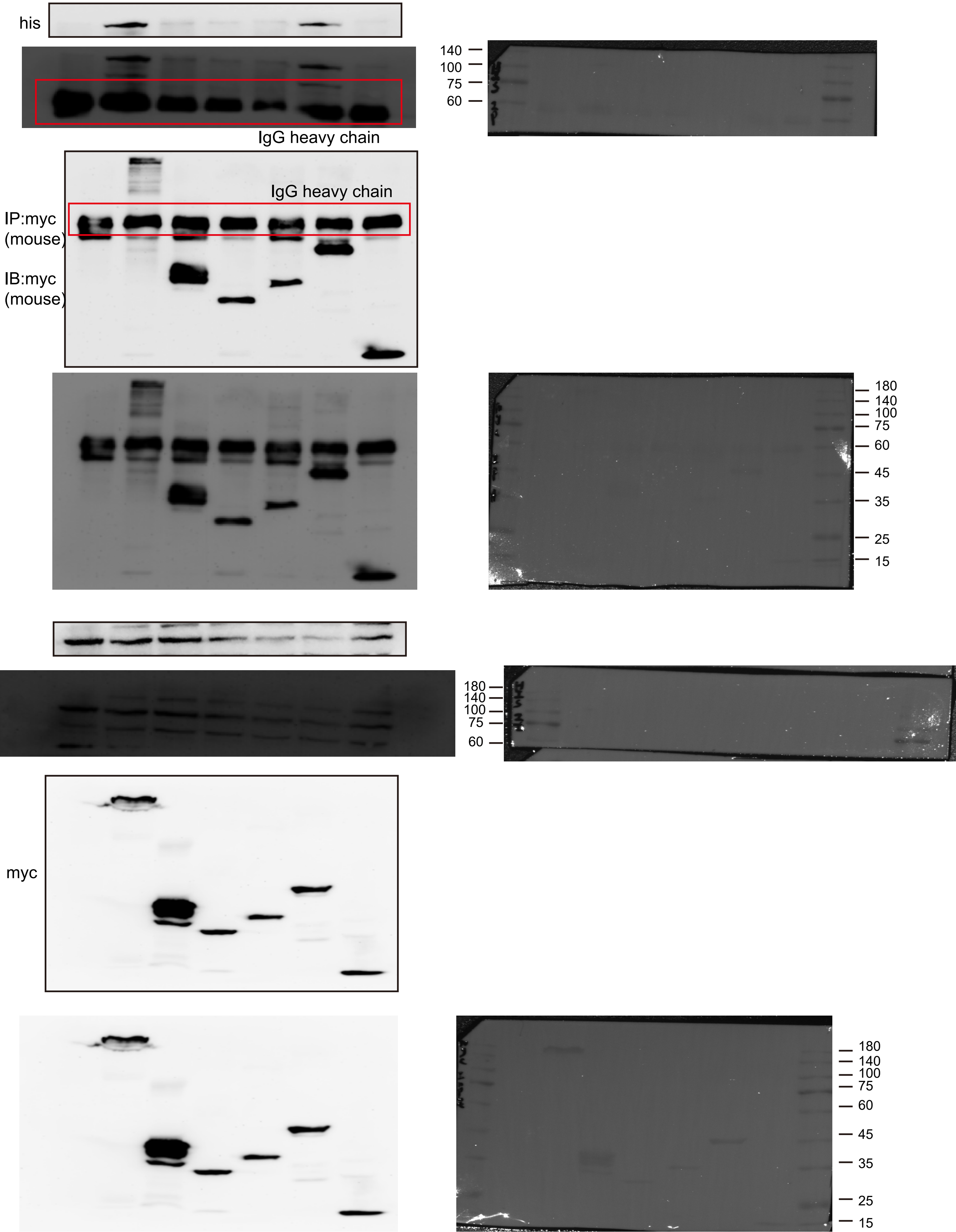

Supplementary Figure 6

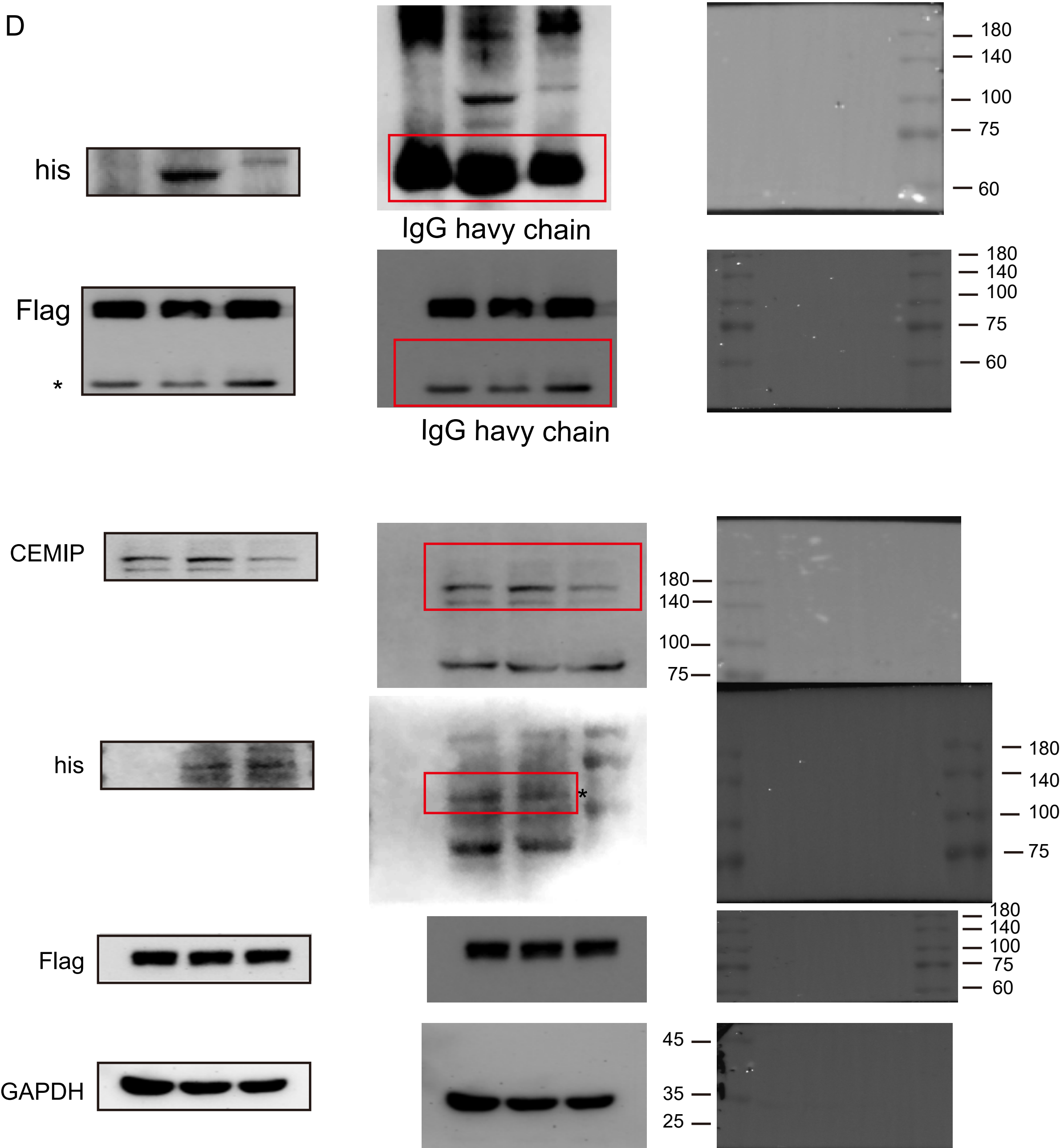

Supplementary Figure 8

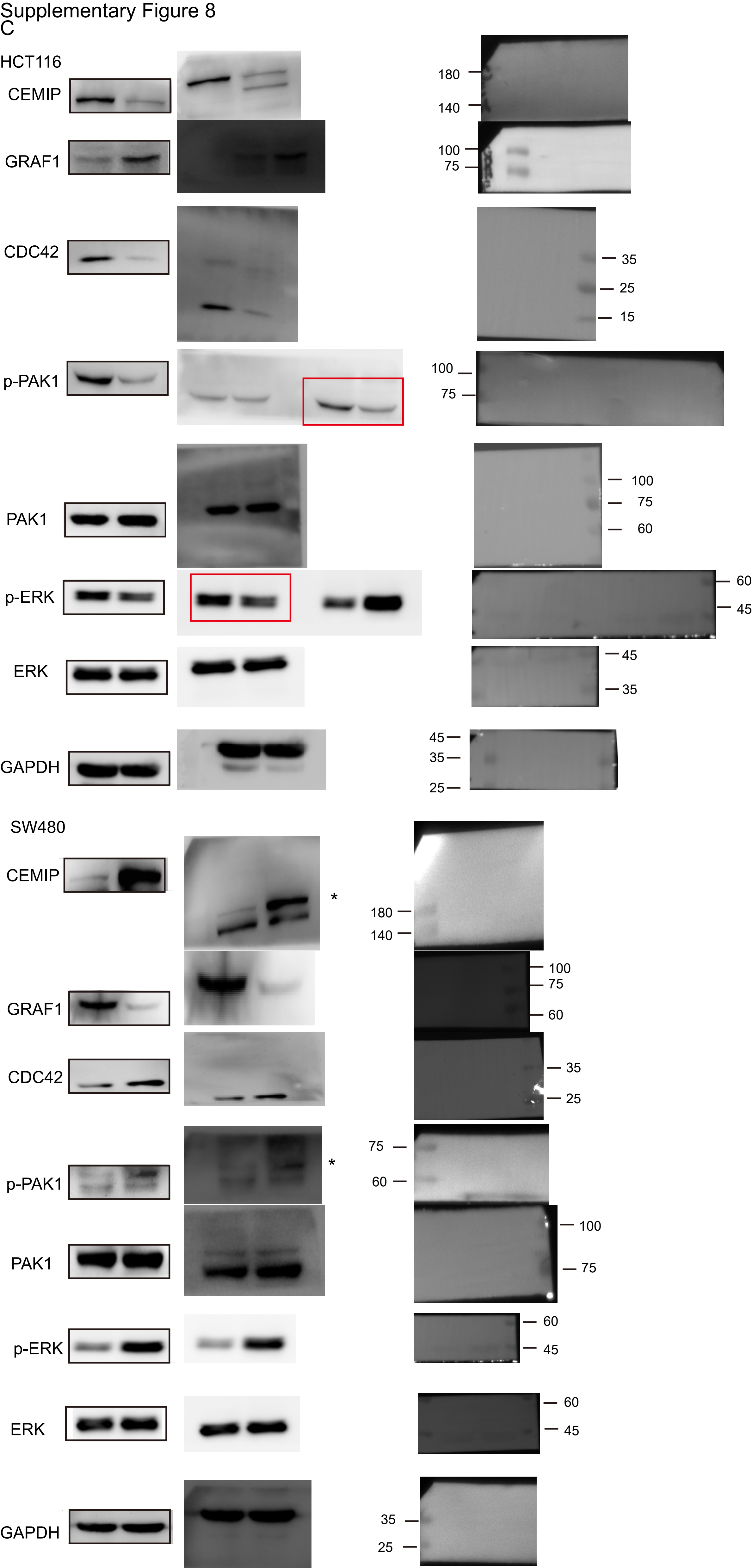

Supplementary Figure 8

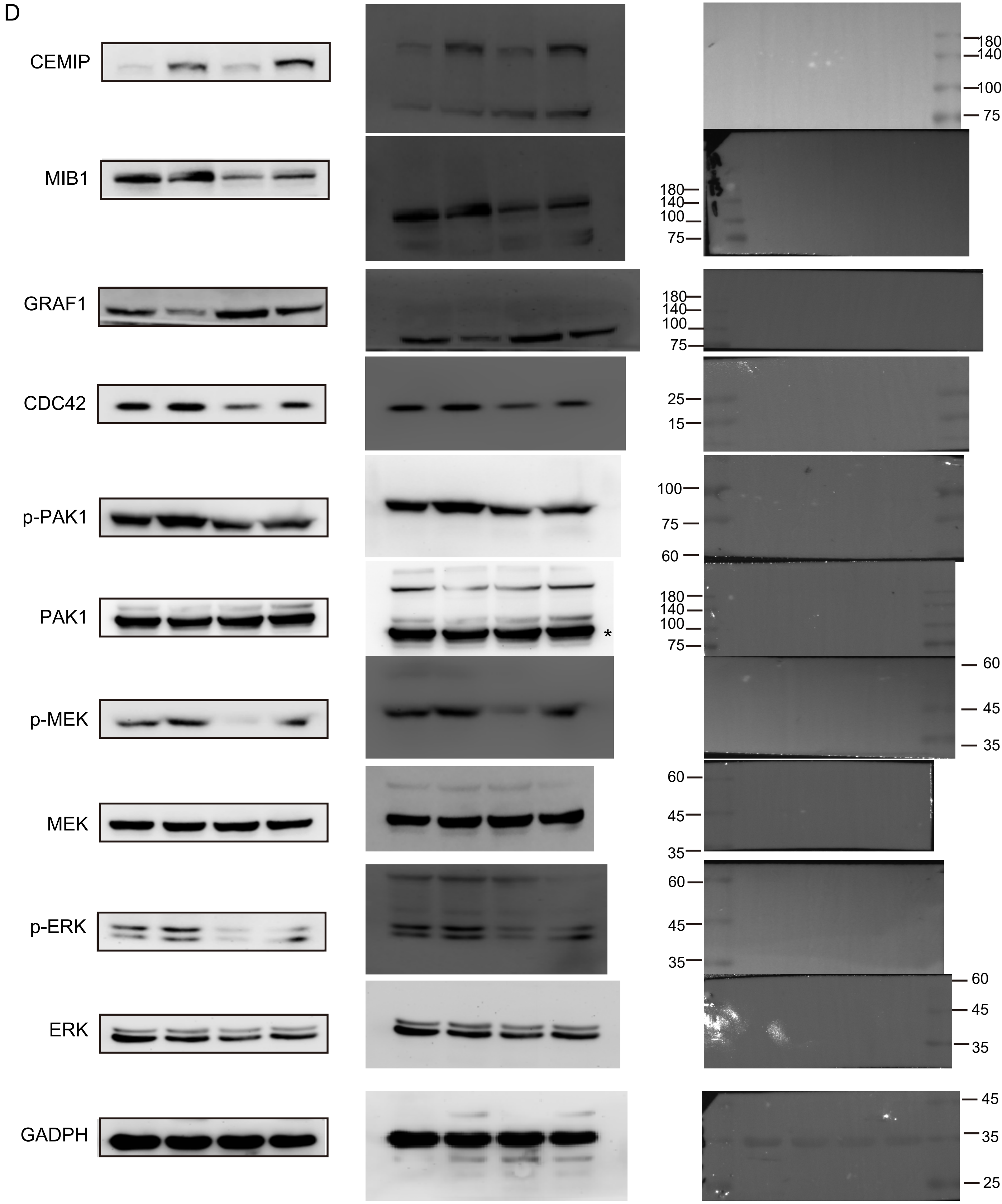

Supplementary Figure 8

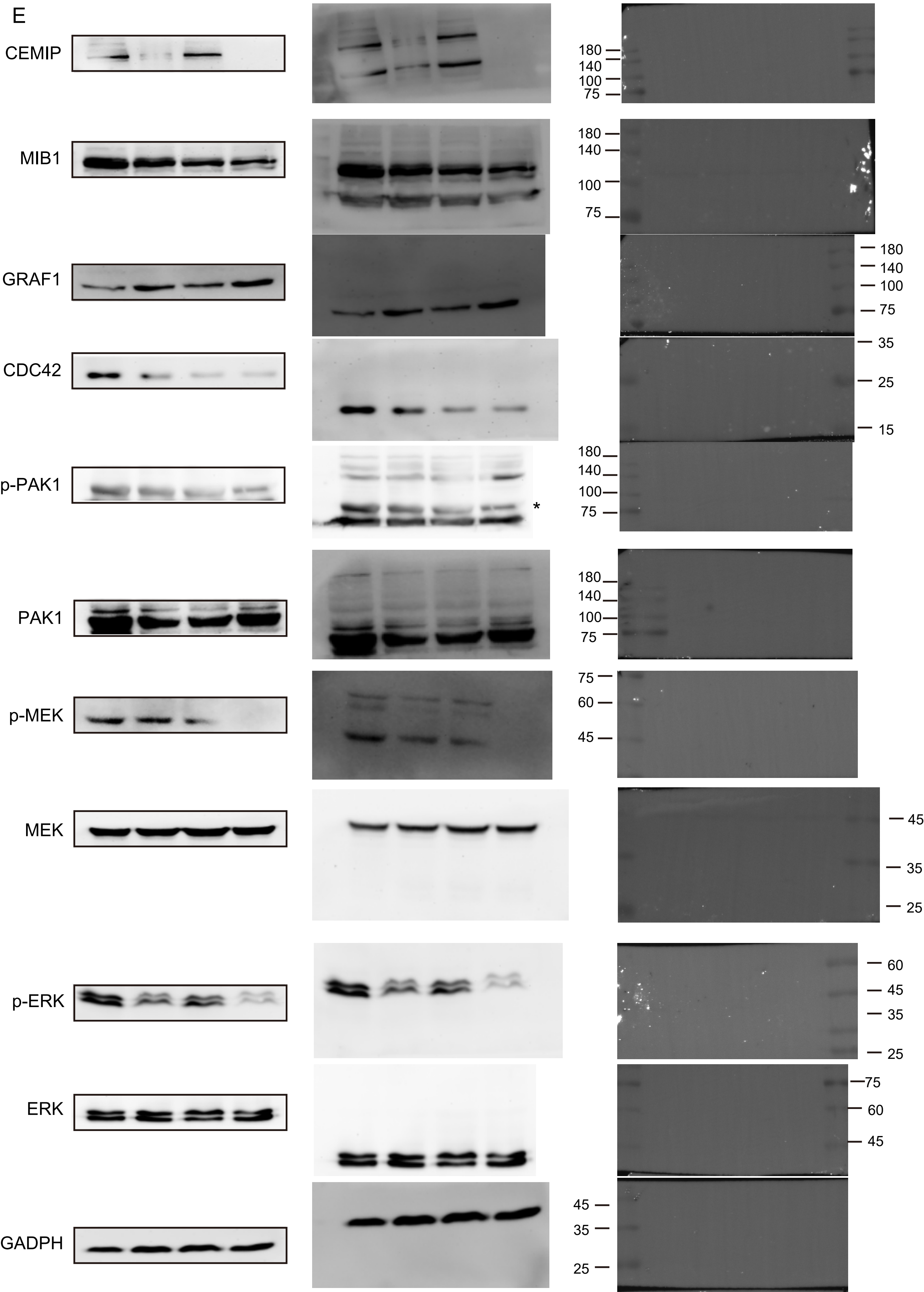

Supplementary Figure 9

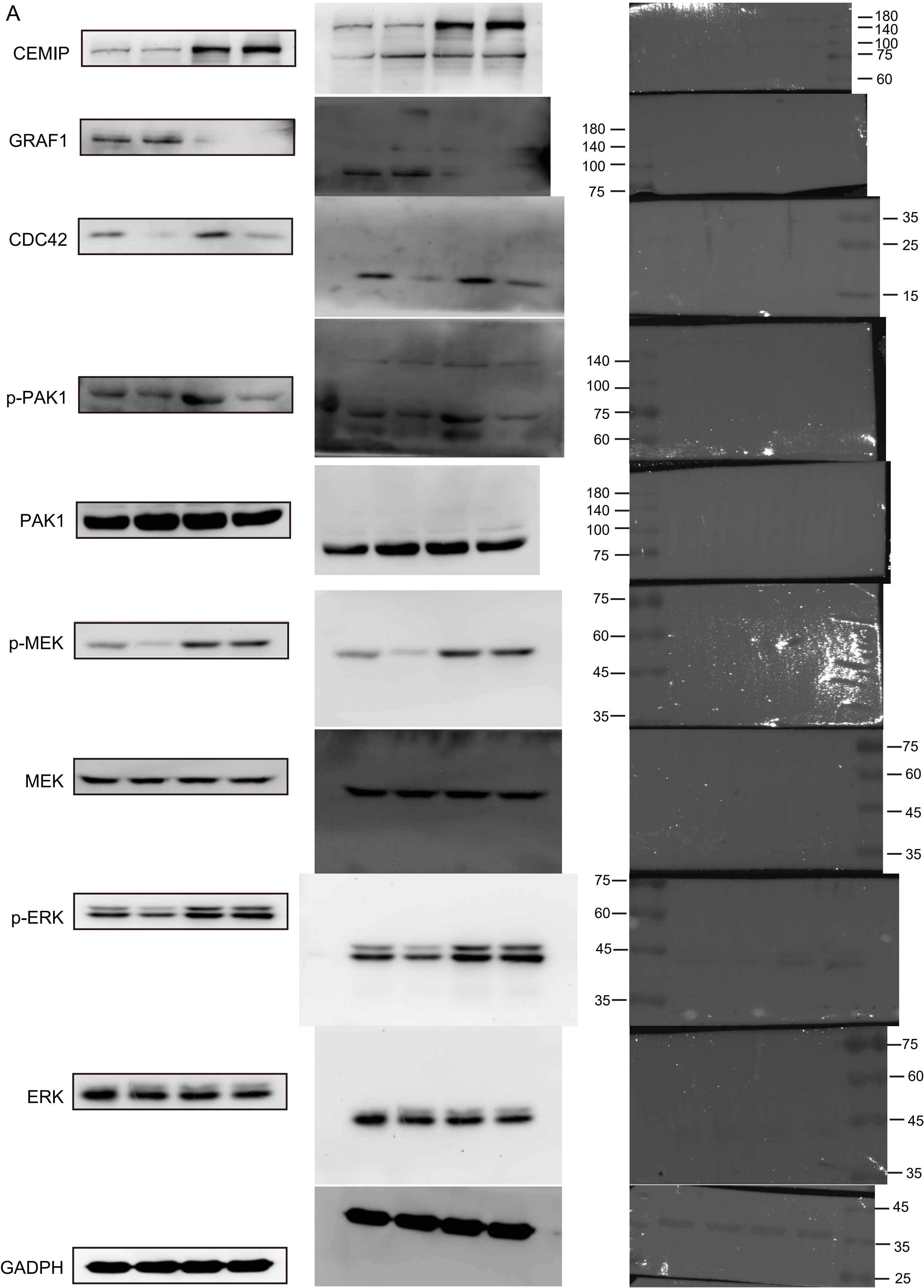

Supplementary Figure 9

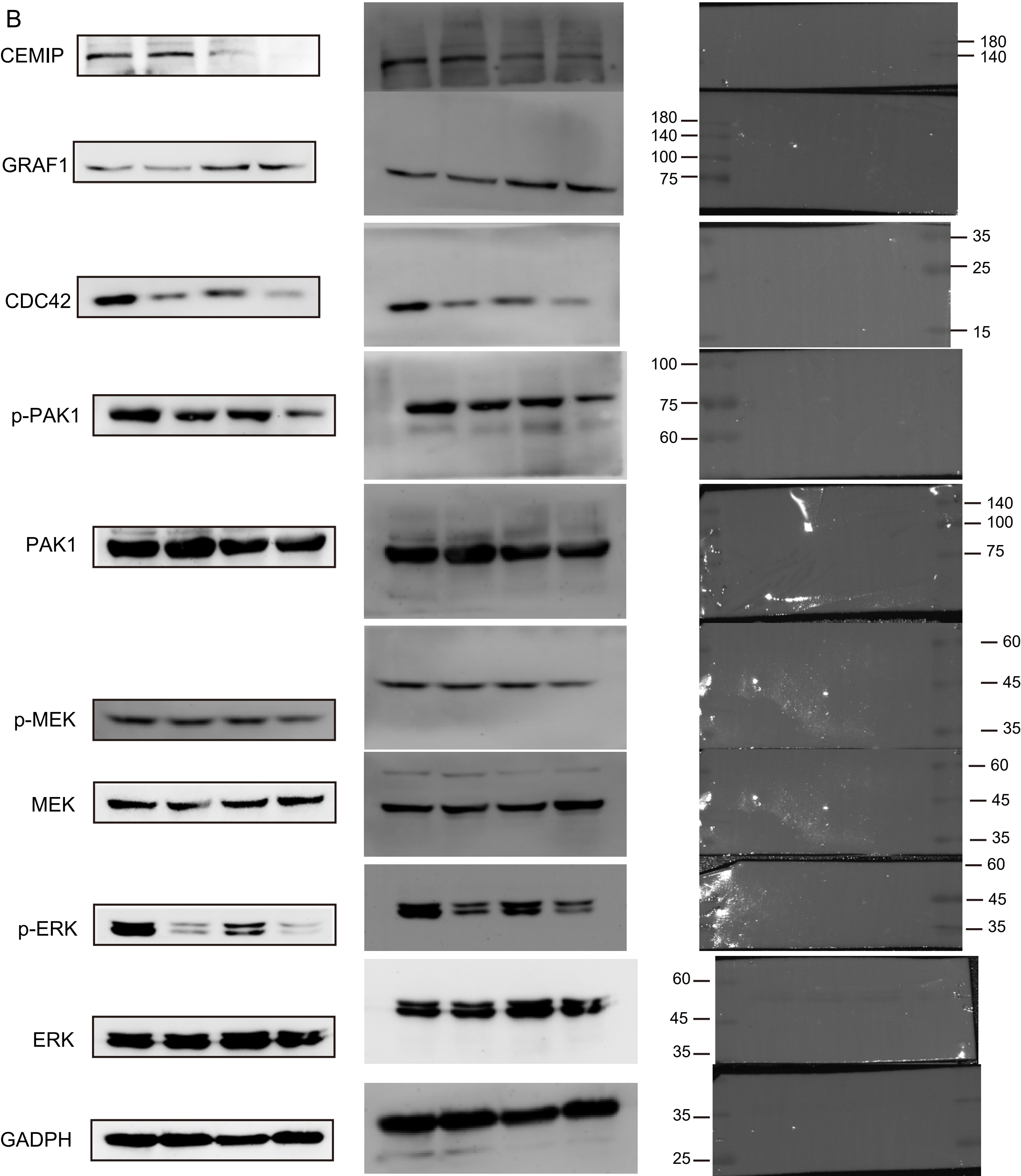

Supplementary Figure 10

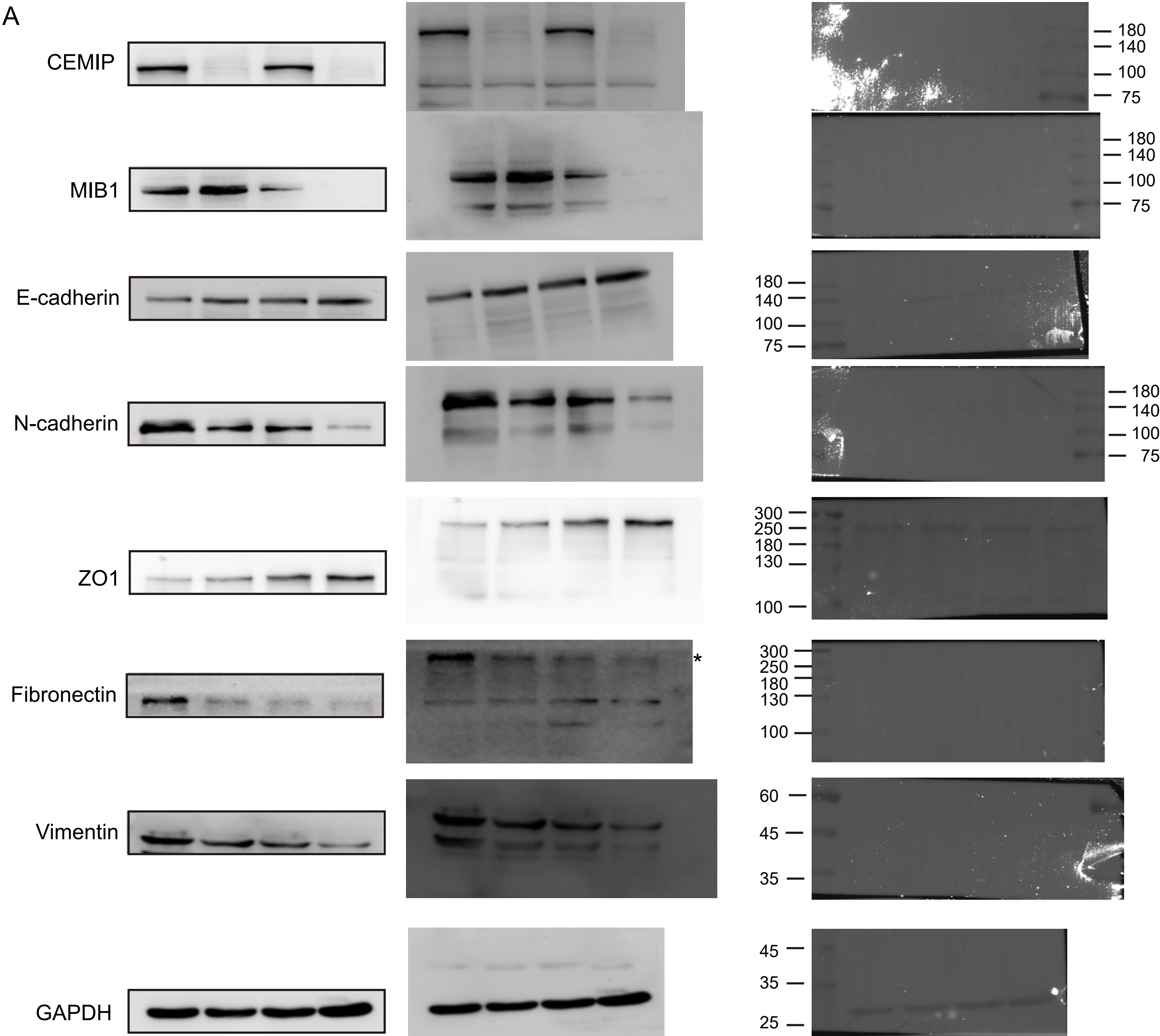

Supplementary Figure 10

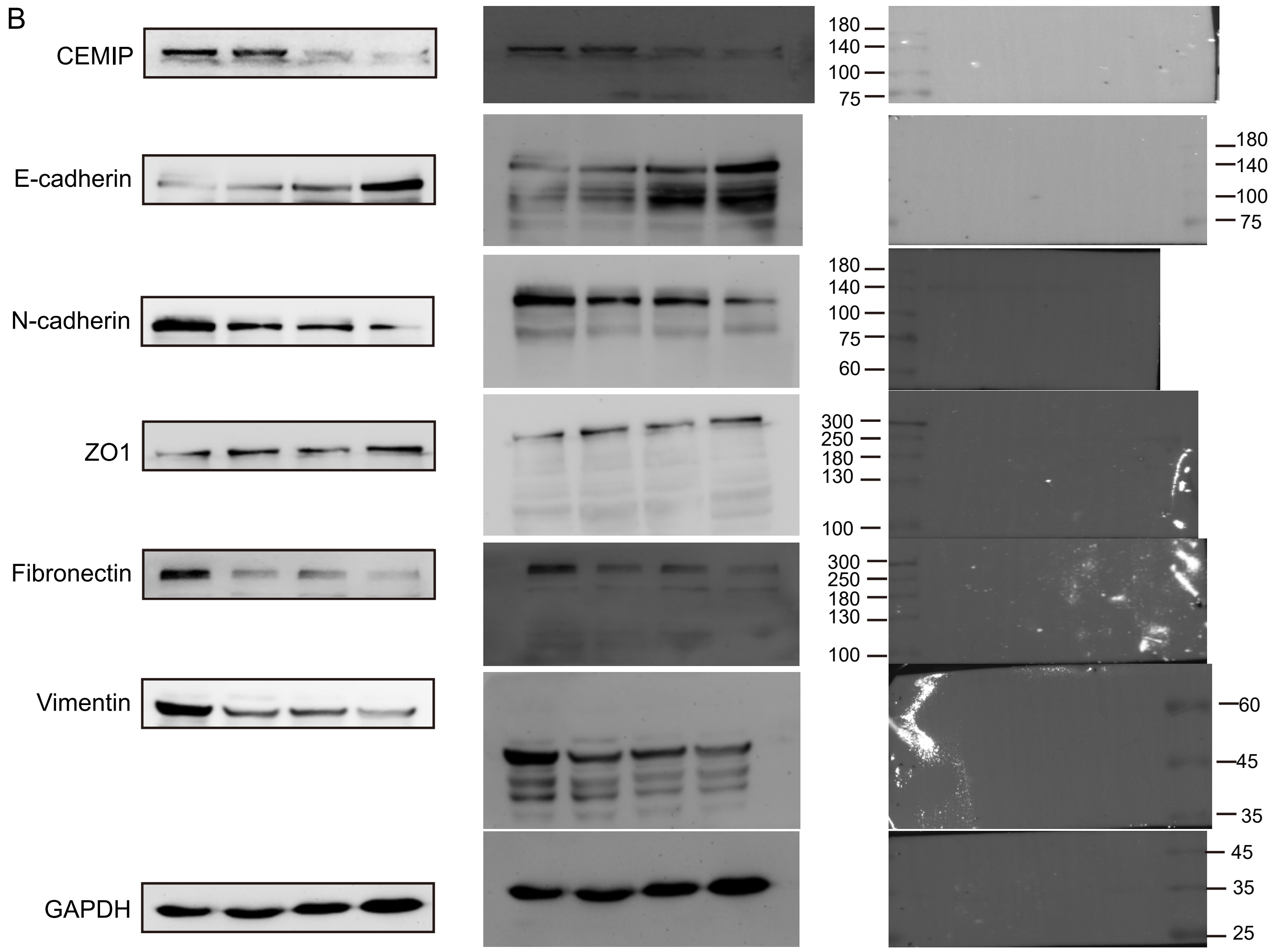

Supplement: Supplementary file 1 — original WB [file 41419_2023_5644_MOESM1_ESM.pdf]
