# Supplementary material for: CEMIP, acting as a scaffold protein for bridging GRAF1 and MIB1, promotes colorectal cancer metastasis via activating CDC42/MAPK pathway
Source: Cell Death Dis. 2023 Feb 27;14(2):167. doi: 10.1038/s41419-023-05644-z (PMC9971195; doi:10.1038/s41419-023-05644-z)
Supplement: Supplementary file 2 — Supplementary Figure S1–10 and Table S1–3 [file 41419_2023_5644_MOESM2_ESM.pdf]

Supplementary Figure S1-10 and Table S1-3

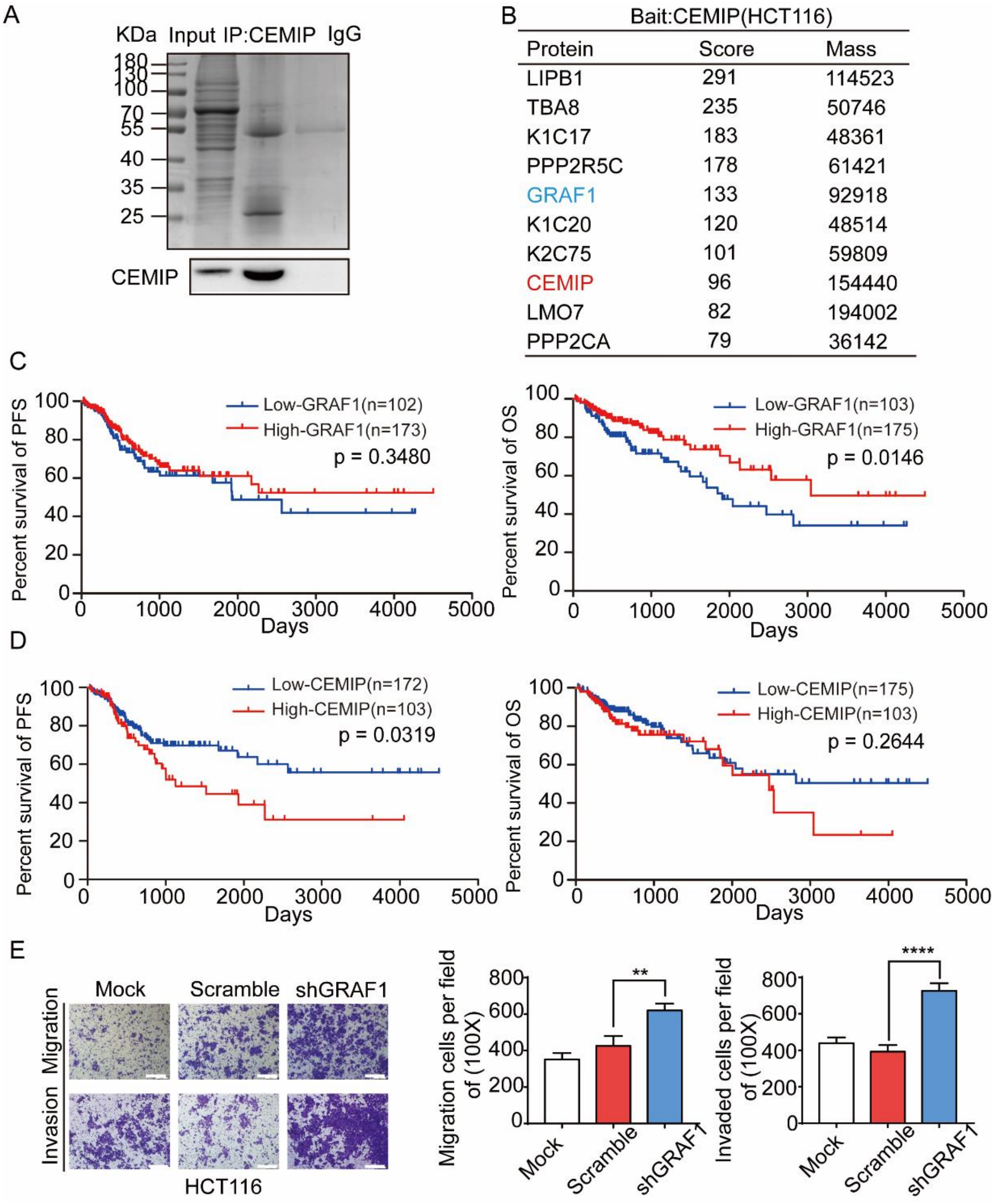

Supplementary Fig. S1 CEMIP has negative correlation with GRAF1 in CRC.
(A-B) A mass spectrometry analysis uncovered multiple CEMIP-interacted proteins in HCT116 cells. The protein score for each protein identified by mass spectrometry analysis was listed. Letters in blue indicated the bait protein. (C-D) The progression-free survival (PFS) and the overall survival (OS) of colon cancer patients of GRAF1 (C) and CEMIP (D) were generated through TCGA (The Cancer Genome Atlas) database from http://www.sangerbox.com/. (E) Transwell chamber migration and invasion of HCT116 cells transfected with shGRAF1 (GRAF1-downregulated plasmid) or Scramble plasmid (negative control). **, $P < 0.01$, ****, $P < 0.0001$. Scale bar, 50 μm.

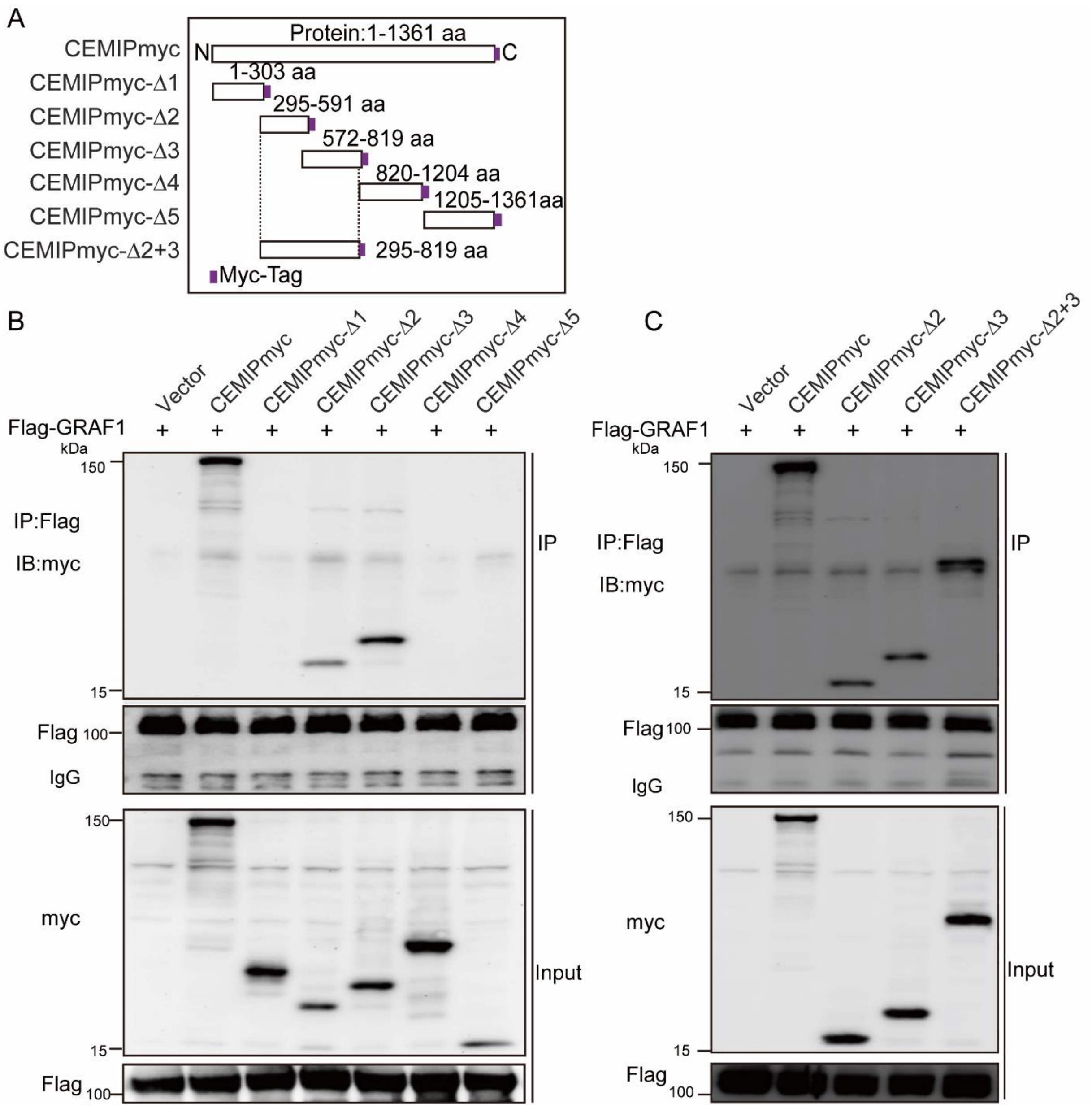

Supplementary Fig. S2 CEMIP interacts with GRAF1 through the 295-819aa domain
(A) Schematic diagram of CEMIP deletion mutants, attached c-myc tag in the C terminal. (B-C) The interactions between exogenous CEMIP deletion mutants and exogenous GRAF1 were detected by Co-IP in HEK293T cells transfected with the indicated constructs.

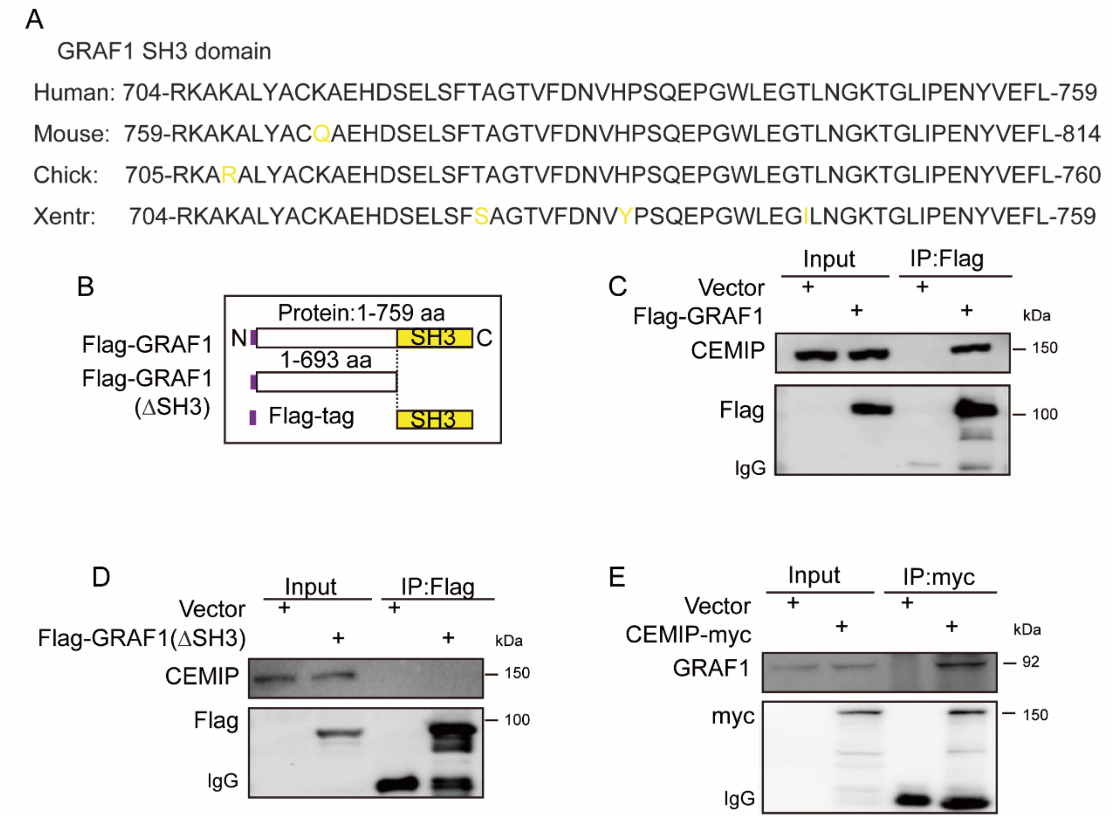

Supplementary Fig. S3 CEMIP interacts with the SH3 domain of GRAF1 and mRNA level of CEMIP and GRAF1.

(A) The specific sequence of SH3 domain of GRAF1 in different species. (B) Schematic diagram of the Flag-GRAF1 and Flag-GRAF1 (ΔSH3) plasmids (GRAF1-overexpression plasmid with lack of SH3 domain). (C-D) The interactions between endogenous CEMIP and exogenous GRAF1 or Flag-GRAF1 (ΔSH3) were detected by Co-IP in HCT116 cells transfected with the indicated constructs. (E)The interactions between endogenous GRAF1 and exogenous CEMIP were detected by Co-IP in HCT116 cells transfected with the indicated constructs.

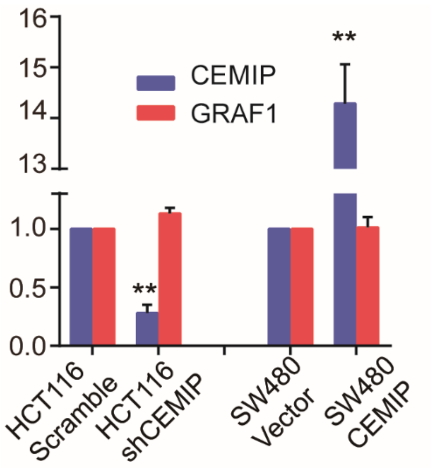

Supplementary Fig. S4 CEMIP has not impact on the mRNA level of GRAF1.
(A) The mRNA level of CEMIP and GRAF1 of CRC cells transfected indicated plasmids analyzed by PCR. **, $P < 0.01$.

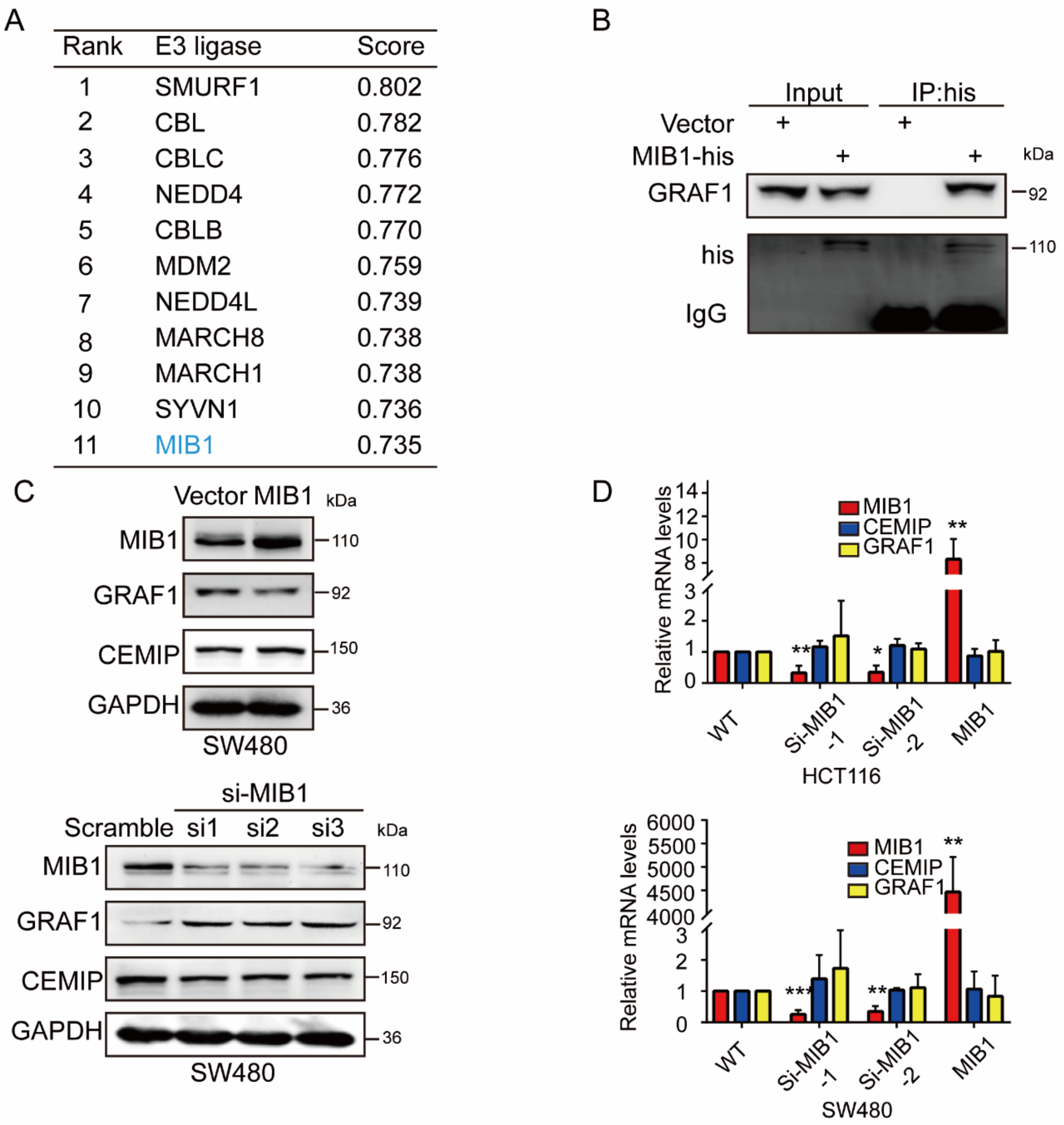

Supplementary Fig. S5 MIB1 interacts with GRAF1 and regulates GRAF1.
(A) The descend order of score calculating the possibility of predicted E3 ligase of GRAF1 (http://ubibrowser.bioit.cn/ubibrowser/strict/networkview/networkview/name/Q9UNA1/jobId/ubibrowse-I2022-12-23-34742-1671805417).(B) The interaction between exogenous MIB1 and endogenous GRAF1 was detected by Co-IP in HCT116 cells transfected with the indicated constructs. (C) The protein level of CEMIP and GRAF1 in SW480 cells transfected with MIB1 plasmid (MIB1-upregulated plasmid containing MIB1 cDNA) or si-MIB1 plasmid (MIB1-downregulated plasmid containing siRNA of MIB1). (D) The mRNA level of CEMIP and GRAF1 detected by PCR analysis transfected with the indicated plasmids in HCT116 or SW480 cells. *, $P < 0.05$; **, $P < 0.01$; ***, $P < 0.001$.

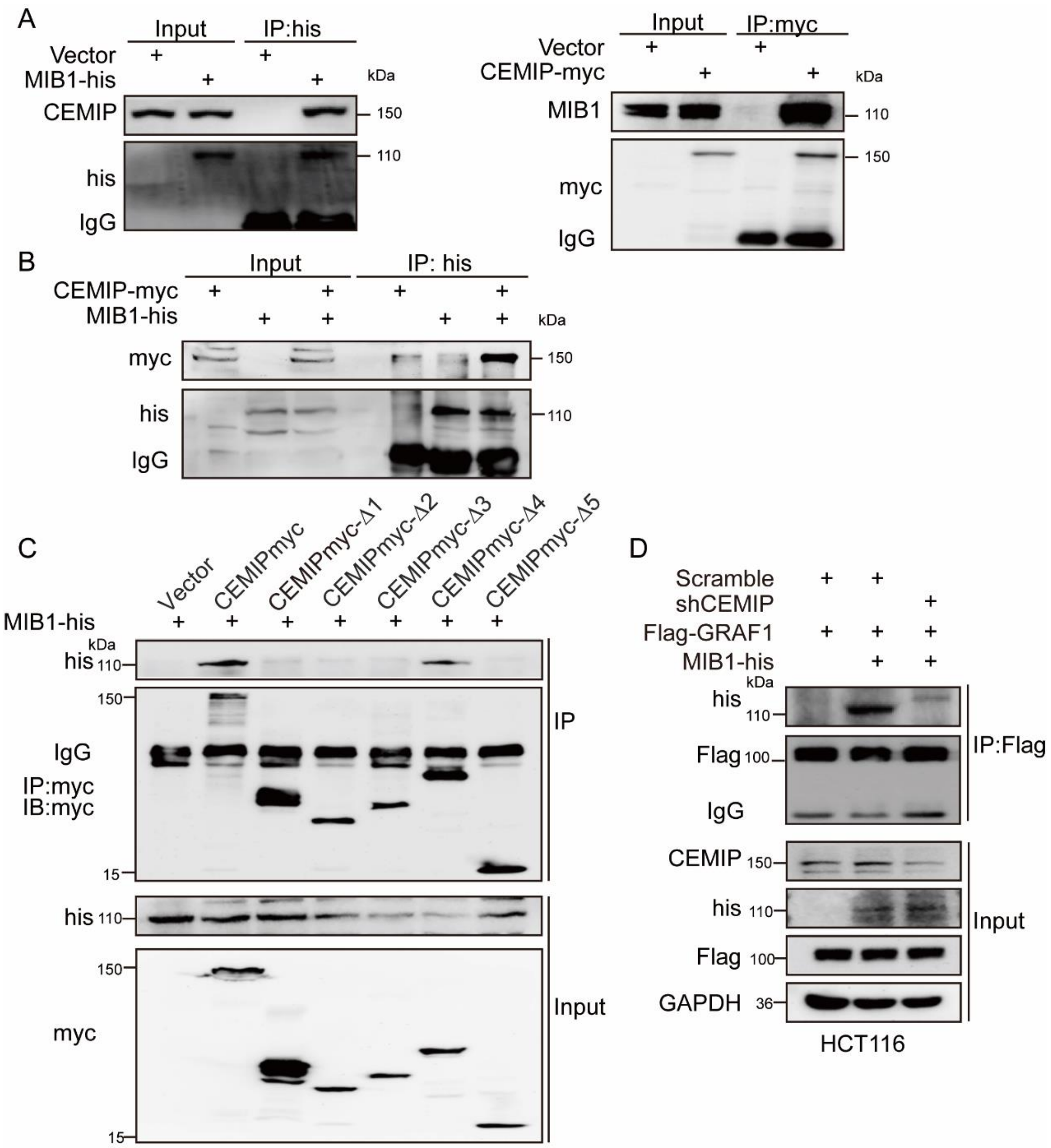

Supplementary Fig. S6 MIB1 interacts with CEMIP.

(A) The interaction between exogenous MIB1 and endogenous CEMIP was detected by Co-IP in HCT116 cells and the interaction between exogenous CEMIP and endogenous MIB1 was detected by Co-IP in HCT116 cells transfected with the indicated constructs. (B) The interaction between exogenous MIB1 and exogenous CEMIP was detected by Co-IP in HEK293T cells. (C) The interactions between exogenous CEMIP deletion mutants and exogenous MIB1 were detected by Co-IP in HEK293T cells transfected with the indicated constructs. (D) Western blotting analysis of proteins derived from Co-IP in HCT116 cells transfected with the indicated plasmids.

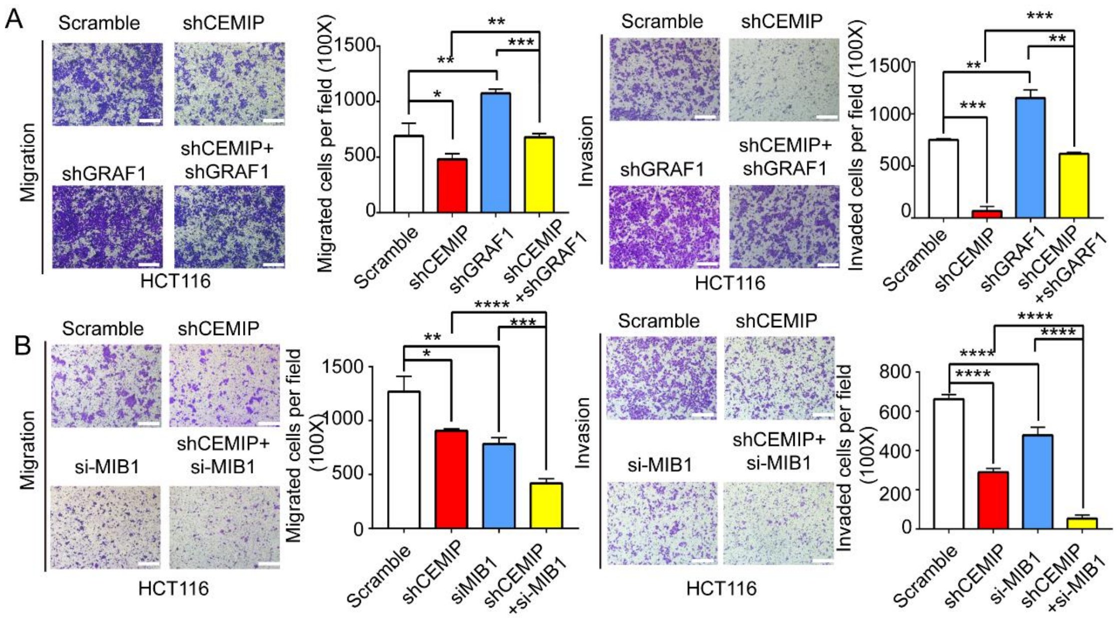

Supplementary Fig. S7 CEMIP and MIB1 synergistically promoted migration and invasion of CRC cells through regulating GRAF1.

(A-B) Transwell chamber migration and invasion of HCT116 cells transfected with the indicated constructs. *, $P<0.05$; **, $P < 0.01$; ***, $P < 0.001$; ****, $P < 0.0001$. Scale bar, 50μm.

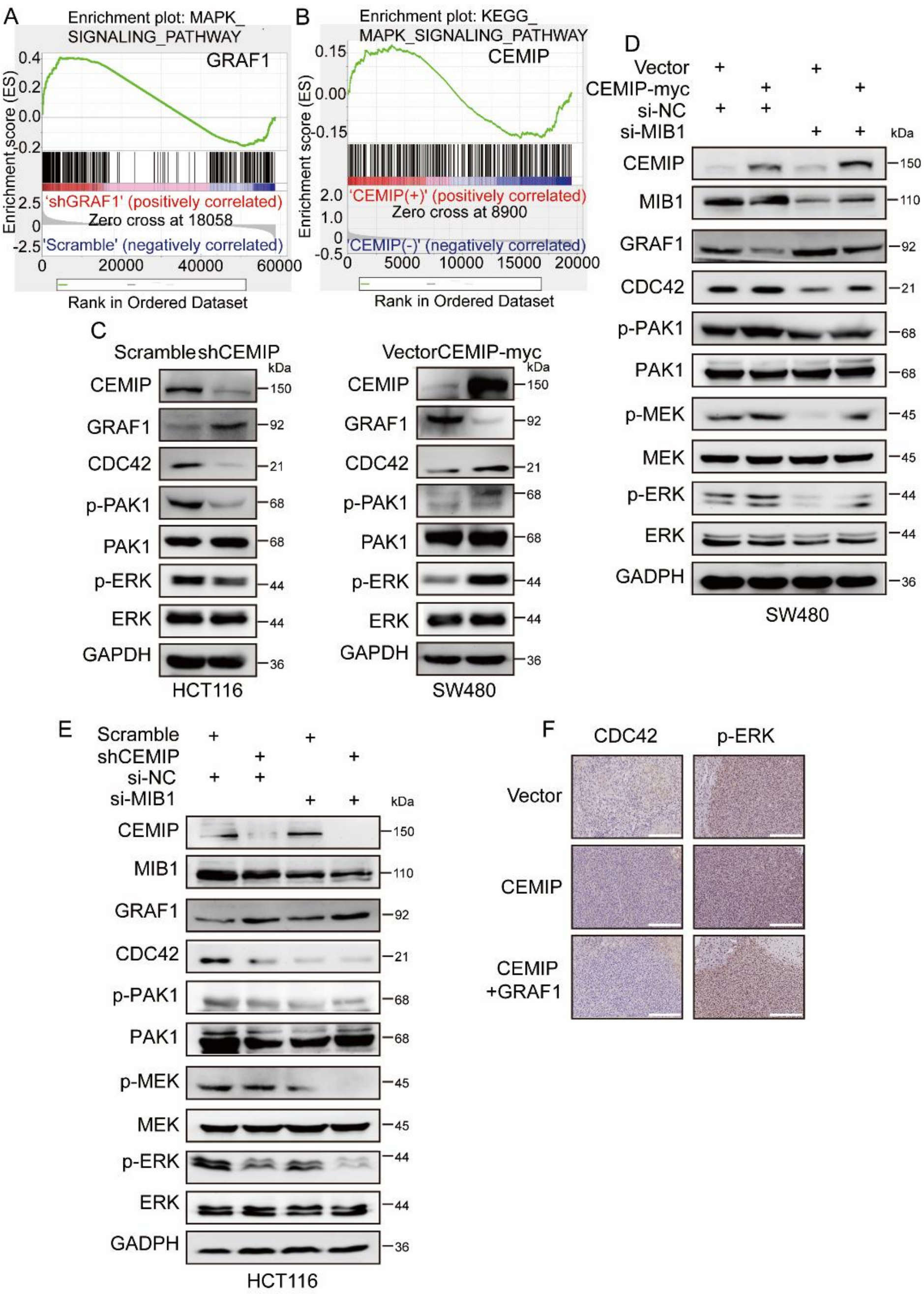

Supplementary Fig. S8 CEMIP activates CDC42/MAPK pathway through inhibiting GRAF1

(A) The correlation between GRAF1 and MAPK pathway through KEGG database analysis by mRNA sequencing of knocked-down GRAF1 in HCT116 cells. (B) The correlation between CEMIP and MAPK pathway through GESA analysis of CRC patients from TCGA database. (C) Western blotting analysis of CEMIP, GRAF1, CDC42, PAK1 and other MAPK pathway proteins in HCT116 (left) and SW480 (right) cells transfected with the indicated plasmids. (D-E) Western blotting analysis of CEMIP, GRAF1, CDC42, PAK1 and MAPK pathway proteins in SW480(D) and HCT116(E) cells transfected with the indicated constructs. (F) Representative immunohistochemical staining for CDC42 and p-ERK in primary colorectal cancer tissues of BALB/c nude mice mentioned above. The group of GRAF1 were not founded in metastatic foci, Scale bar, 50 μm.

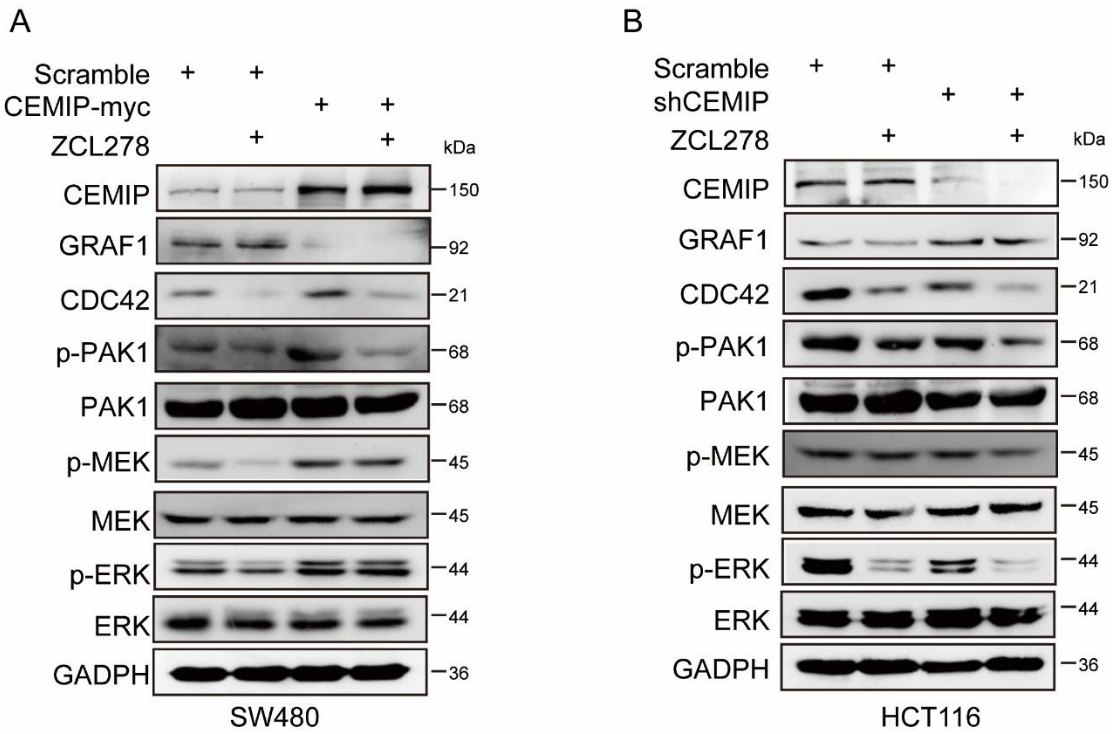

Supplementary Fig. S9 CDC42 inhibitor suppresses GRAF1/CDC42/MAPK pathway
(A-B) Western blotting analysis of CEMIP and EMT related proteins in SW480 (A) and HCT116(B) cells transfected with the indicated constructs with or without ZCL278.

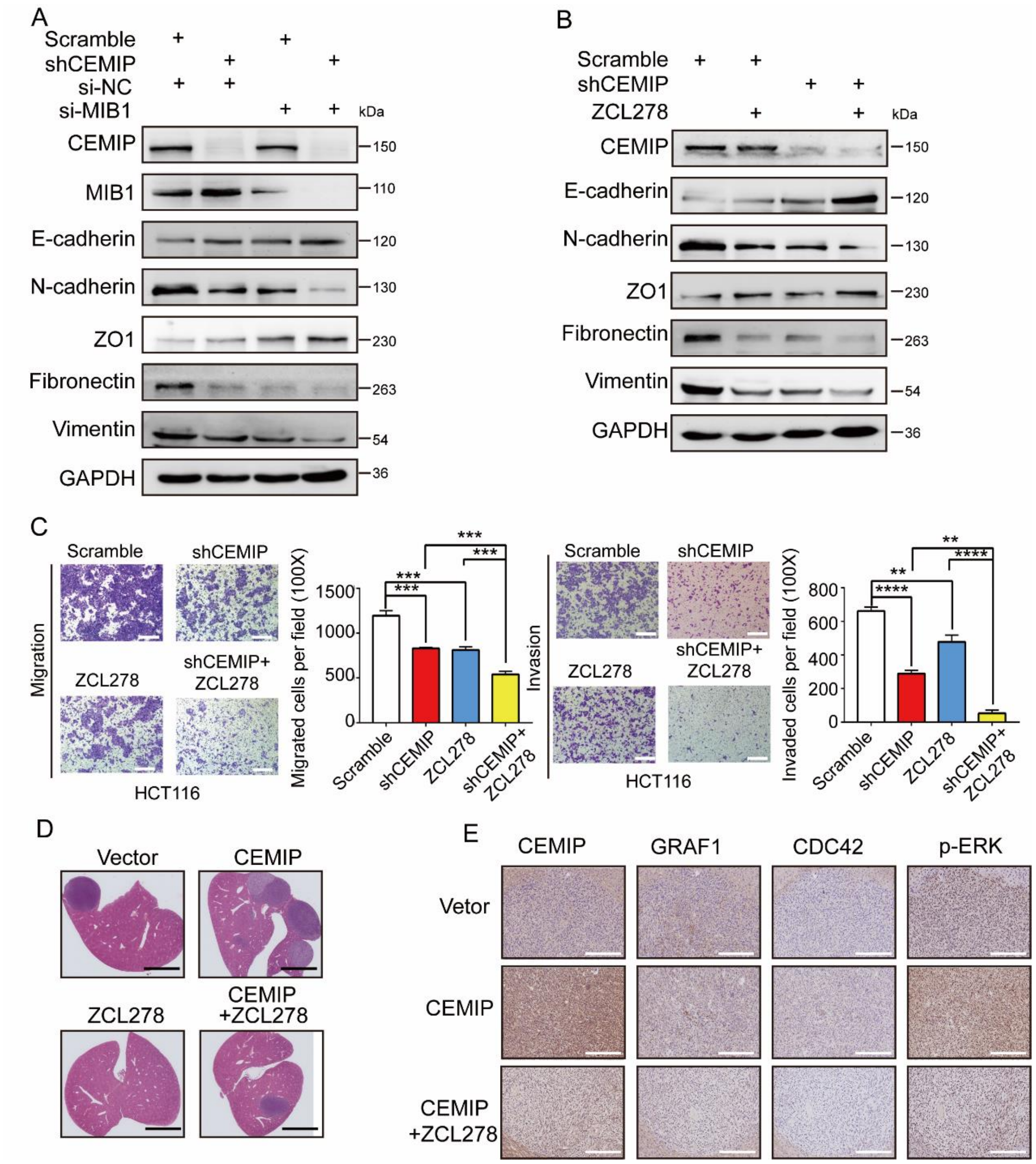

Supplementary Fig. S10 CEMIP promotes metastasis of CRC cells through GRAF1/CDC42/MAPK pathway-regulated EMT (A) Western blotting analysis of EMT associated proteins in HCT116 cells transfected with the indicated constructs. (B) Western blotting analysis of EMT associated proteins in HCT116 cells transfected with the indicated constructs. (C) Transwell chamber migration and invasion of HCT116 cells transfected with the indicated constructs with or without ZCL278. **, P < 0.01; ***, P < 0.001; ****, P < 0.0001. (D) Representative HE staining of liver tissues of BALB/C nude mice (day 28, n = 5), and mice were treated with or without ZCL278. Scale bar, 1 mm. The group of ZCL278 were not founded in metastatic foci, because the surface of metastatic foci were not embedding. (E) Representative immunohistochemical staining in primary colorectal cancer tissues of BALB/C nude mice for CEMIP, GRAF1, CDC42 and p-ERK (day 28, n = 5), and mice were treated with or without ZCL278. Scale bar, 50 μm.

Supplementary Table. S1 mass-spectrometric (MS) peptide outcome of CEMIP-interacting proteins

prot_num	protein	prot_score	prot_mass
31	sp|Q86W92|LIPB1_HUMAN	291	114523
34	sp|Q9NY65|TBA8_HUMAN	235	50746
39	sp|Q04695|K1C17_HUMAN	183	48361
40	sp|Q13362|2A5G_HUMAN	178	61421
**51**	**sp|Q9UNA1|RHG26_HUMAN**	**133**	**92918**
56	sp|P35900|K1C20_HUMAN	120	48514
62	sp|O95678|K2C75_HUMAN	101	59809
**66**	**sp|Q8WUJ3|CEMIP_HUMAN**	**96**	**154440**
73	sp|Q8WWI1|LMO7_HUMAN	82	194002
74	sp|P67775|PP2AA_HUMAN	79	36142
76	sp|P01602|KV110_HUMAN	76	12944
79	sp|Q9H361|PABP3_HUMAN	73	70215
91	sp|P62873|GBB1_HUMAN	59	38151
92	sp|Q8IZS6|TC1D3_HUMAN	58	23161
95	sp|P22090|RS4Y1_HUMAN	55	29665
96	sp|Q9NVI7|ATD3A_HUMAN	54	71610
99	sp|Q8WVV4|POF1B_HUMAN	54	68878
106	sp|P24534|EF1B_HUMAN	50	24919
111	sp|P05109|S10A8_HUMAN	48	10885
112	sp|P05198|IF2A_HUMAN	48	36374
121	sp|P08670|VIME_HUMAN	47	53676
124	sp|P61619|S61A1_HUMAN	46	52687
127	sp|O60506|HNRPQ_HUMAN	44	69788
132	sp|P06756|ITAV_HUMAN	42	117048
133	sp|P01876|IGHA1_HUMAN	41	38486
134	sp|P22570|ADRO_HUMAN	41	54259
136	sp|Q9H078|CLPB_HUMAN	41	79193
138	sp|Q96L21|RL10L_HUMAN	41	24959
140	sp|Q6EMB2|TTLL5_HUMAN	40	144457
141	sp|O94832|MYO1D_HUMAN	40	116927
148	sp|Q9H6S1|AZI2_HUMAN	37	45420
149	sp|A9Z1Z3|FR1L4_HUMAN	37	202166
151	sp|Q9H9J2|RM44_HUMAN	37	37854
152	sp|Q9P2B2|FPRP_HUMAN	36	99464
154	sp|H7BZ55|CRCC2_HUMAN	36	186900
156	sp|Q9BX70|BTBD2_HUMAN	35	56580
157	sp|Q9NTM9|CUTC_HUMAN	35	29721
158	sp|Q86UX7|URP2_HUMAN	35	76475
160	sp|P46779|RL28_HUMAN	35	15795
162	sp|Q9C0C2|TB182_HUMAN	34	182711
163	sp|P19440|GGT1_HUMAN	34	61714
164	sp|Q8TB92|HMGC2_HUMAN	34	40059
165	sp|P48736|PK3CG_HUMAN	34	127571
167	sp|Q96QF7|ACRC_HUMAN	33	77150
175	sp|Q9P2E9|RRBP1_HUMAN	32	152780
176	sp|P04920|B3A2_HUMAN	32	137493
177	sp|Q99819|GDIR3_HUMAN	32	25367
179	sp|Q8TF72|SHRM3_HUMAN	32	218321
180	sp|Q9NRL3|STRN4_HUMAN	32	81287
181	sp|P11498|PYC_HUMAN	32	130293
183	sp|Q2NL68|PRSR3_HUMAN	32	51158
185	sp|Q92839|HYAS1_HUMAN	32	65645
186	sp|Q96EK7|F120B_HUMAN	32	105769
188	sp|Q9HCE1|MOV10_HUMAN	32	114512
189	sp|O95405|ZFYV9_HUMAN	32	158926
190	sp|Q9H000|MKRN2_HUMAN	32	48278

prot_num	protein	prot_score	prot_mass
192	sp|P01605|KV113_HUMAN	32	11941
194	sp|Q07955|SRSF1_HUMAN	32	27842
195	sp|Q9NWX5|ASB6_HUMAN	32	47676
196	sp|Q9NXG2|THUM1_HUMAN	32	39690
197	sp|Q14738|2A5D_HUMAN	32	70289
198	sp|P52746|ZN142_HUMAN	32	193289
199	sp|Q6ZUX3|F179A_HUMAN	32	111996
200	sp|Q9NZN4|EHD2_HUMAN	32	61294
201	sp|Q07283|TRHY_HUMAN	32	254233
202	sp|P14061|DHB1_HUMAN	32	35270
203	sp|Q9P2M7|CING_HUMAN	31	136532
206	sp|Q2TAC6|KIF19_HUMAN	31	111834
207	sp|Q7Z7A1|CNTRL_HUMAN	31	269860
209	sp|Q12836|ZP4_HUMAN	31	60730
210	sp|Q9UPT8|ZC3H4_HUMAN	31	140797
211	sp|P23246|SFPQ_HUMAN	31	76216
212	sp|Q9UBG7|RBPJL_HUMAN	31	57627
213	sp|Q9C0D2|CE295_HUMAN	31	296535
215	sp|O95999|BCL10_HUMAN	30	26520
217	sp|O43734|CIKS_HUMAN	30	65082
218	sp|O60307|MAST3_HUMAN	30	143847
220	sp|P18077|RL35A_HUMAN	30	12587
221	sp|Q8WXI9|P66B_HUMAN	29	65562
222	sp|B1ANS9|WDR64_HUMAN	29	124807
223	sp|P78509|RELN_HUMAN	29	394980
224	sp|P35269|T2FA_HUMAN	29	58262

Supplementary Table. S2. Clinicopathological features and the correlation with CEMIP expression.

Characteristics	No. of patients (%)	CEMIP expression		P-value
		low	high	
**All patients**	87	34	53	
**Gender**				
**Male**	56	23	33	0.5349
**Female**	31	11	20	
**Age (median 54.5, years)**				
**<60**	58	26	32	0.1203
**≥60**	29	8	21	
**Tumor size (cm) (n=83)**				
**<5**	51	22	29	0.4272
**≥5**	32	11	21	
**Tumor location**				
**Colon**	52	22	30	0.4521
**Rectum**	35	12	23	
**Histology differentiation**				
**Poor**	32	15	17	0.4382
**Moderate**	47	17	30	
**Well**	8	2	6	
**Depth of invasion**				
**T1~T3**	22	8	14	0.9499
**T4a**	43	17	26	
**T4b**	22	9	13	
**Lymphatic invasion**				
**N0**	22	8	14	0.2015
**N1**	26	7	19	
**N2**	39	19	20	
**TNM stage**				
**I+II**	9	4	5	0.8666
**III**	29	12	17	
**IV**	49	18	31	

Notes: TNM, tumor-nodes-metastasis. The 2017 American Joint Committee on Cancer (AJCC) 8th TNM staging system was used for determining tumor stages. The correlation between CEMIP expression and clinicopathological features was analyzed by the χ2-test.

Supplementary Table. S3. Clinicopathological features and the correlation with GRAF1 expression.

Characteristics	No. of patients (%)	GRAF1 expression		P-value
		low	high	
**All patients**	87	45	42	
**Gender**				
**Male**	56	25	31	0.0756
**Female**	31	20	11	
**Age (median 54.5,years)**				
**<60**	58	38	20	0.5294
**≥60**	29	17	12	
**Tumor size (cm) (n=83)**				
**<5**	51	26	25	0.8491
**≥5**	32	17	15	
**Tumor location**				
**Colon**	52	26	26	0.6949
**Rectum**	35	19	16	
**Histology differentiation**				
**Poor**	32	18	14	0.2737
**Moderate**	47	25	22	
**Well**	8	2	6	
**Depth of invasion**				
**T1~T3**	22	12	10	0.8677
**T4a**	43	21	22	
**T4b**	22	12	10	
**Lymphatic invasion**				
**N0**	22	9	13	0.3612
**N1**	26	16	10	
**N2**	39	20	19	
**TNM stage**				
**I+II**	9	3	6	0.4860
**III**	29	15	14	
**IV**	49	27	22	

Notes: TNM, tumor-nodes-metastasis. The 2017 American Joint Committee on Cancer (AJCC) 8th TNM staging system was used for determining tumor stages. The correlation between GRAF1 expression and clinicopathological features was analyzed by the $\chi 2$-test.
