# Supplementary material for: CEMIP, acting as a scaffold protein for bridging GRAF1 and MIB1, promotes colorectal cancer metastasis via activating CDC42/MAPK pathway
Source: Cell Death Dis. 2023 Feb 27;14(2):167. doi: 10.1038/s41419-023-05644-z (PMC9971195; doi:10.1038/s41419-023-05644-z)
Supplement: Supplementary file 3 — Supplementary Table.S4 [file 41419_2023_5644_MOESM3_ESM.docx]

| Supplementary Table. S4. The MAPK signaling pathway of KEGG analysis of the mRNA sequencing of down-regulated GRAF1 comparing to Scramble in HCT116 cells. | | | | | |
| --- | --- | --- | --- | --- | --- |
| NAME | PROBE | RANK IN GENE | RANK METRIC SCORE | RUNNING ES | CORE |
| row_0 | DDIT3(ENSG00000175197) | 0 | 3.75768137 | 0.0290374 | **Yes** |
| row_1 | FOS(ENSG00000170345) | 35 | 2.556934357 | 0.04821435 | **Yes** |
| row_2 | AREG(ENSG00000109321) | 90 | 2.001704216 | 0.0627586 | **Yes** |
| row_3 | GADD45B(ENSG00000099860) | 100 | 1.952501297 | 0.07769252 | **Yes** |
| row_4 | NR4A1(ENSG00000123358) | 128 | 1.851655245 | 0.09153921 | **Yes** |
| row_5 | DUSP1(ENSG00000120129) | 204 | 1.643016458 | 0.10295241 | **Yes** |
| row_6 | GADD45A(ENSG00000116717) | 215 | 1.618007541 | 0.11528444 | **Yes** |
| row_7 | DUSP5(ENSG00000138166) | 247 | 1.56477046 | 0.12684579 | **Yes** |
| row_8 | IL1RAP(ENSG00000196083) | 266 | 1.545220971 | 0.1384785 | **Yes** |
| row_9 | ANGPT4(ENSG00000101280) | 273 | 1.535480261 | 0.15024123 | **Yes** |
| row_10 | JUN(ENSG00000177606) | 288 | 1.52305913 | 0.16177112 | **Yes** |
| row_11 | PDGFA(ENSG00000197461) | 355 | 1.432908773 | 0.1717147 | **Yes** |
| row_12 | FLT3(ENSG00000122025) | 432 | 1.356689215 | 0.1808982 | **Yes** |
| row_13 | NTRK1(ENSG00000198400) | 496 | 1.301102281 | 0.18987457 | **Yes** |
| row_14 | MAP2K3(ENSG00000034152) | 506 | 1.29367733 | 0.19971746 | **Yes** |
| row_15 | RRAS(ENSG00000126458) | 719 | 1.158338428 | 0.20504141 | **Yes** |
| row_16 | EPHA2(ENSG00000142627) | 741 | 1.151118755 | 0.21357736 | **Yes** |
| row_17 | ERBB4(ENSG00000178568) | 756 | 1.144922853 | 0.22218521 | **Yes** |
| row_18 | RELB(ENSG00000104856) | 799 | 1.124245644 | 0.23015422 | **Yes** |
| row_19 | KDR(ENSG00000128052) | 921 | 1.072750568 | 0.2363737 | **Yes** |
| row_20 | EFNA5(ENSG00000184349) | 997 | 1.04834938 | 0.24319161 | **Yes** |
| row_21 | MKNK2(ENSG00000099875) | 1021 | 1.038130164 | 0.25082025 | **Yes** |
| row_22 | NFKB2(ENSG00000077150) | 1160 | 0.9861781 | 0.25607988 | **Yes** |
| row_23 | CACNA1G(ENSG00000006283) | 1233 | 0.962763548 | 0.26228777 | **Yes** |
| row_24 | RPS6KA5(ENSG00000100784) | 1241 | 0.961363196 | 0.2695969 | **Yes** |
| row_25 | FGF8(ENSG00000107831) | 1386 | 0.920750439 | 0.2742483 | **Yes** |
| row_26 | VEGFA(ENSG00000112715) | 1502 | 0.896048546 | 0.27920496 | **Yes** |
| row_27 | PLA2G4C(ENSG00000105499) | 1676 | 0.861117542 | 0.2828994 | **Yes** |
| row_28 | FGF21(ENSG00000105550) | 1777 | 0.843603253 | 0.28770742 | **Yes** |
| row_29 | PRKCA(ENSG00000154229) | 1837 | 0.828831077 | 0.29310277 | **Yes** |
| row_30 | ATF4(ENSG00000128272) | 1860 | 0.82410264 | 0.29909462 | **Yes** |
| row_31 | CACNA2D1(ENSG00000153956 | 2033 | 0.790373504 | 0.30225947 | **Yes** |
| row_32 | CDC42(ENSG00000070831) | 2382 | 0.735497117 | 0.3019891 | **Yes** |
| row_33 | NTRK2(ENSG00000148053) | 2467 | 0.721298099 | 0.30612576 | **Yes** |
| row_34 | FLNA(ENSG00000196924) | 2468 | 0.720927238 | 0.3116967 | **Yes** |
| row_35 | CACNB4(ENSG00000182389) | 2480 | 0.719380975 | 0.31706753 | **Yes** |
| row_36 | CSF1R(ENSG00000182578) | 2502 | 0.717039526 | 0.32224914 | **Yes** |
| row_37 | PPP3CC(ENSG00000120910) | 2624 | 0.69931072 | 0.32558286 | **Yes** |
| row_38 | STK4(ENSG00000101109) | 2785 | 0.675765932 | 0.3280674 | **Yes** |
| row_39 | MAP3K13(ENSG00000073803) | 2790 | 0.675037086 | 0.3332153 | **Yes** |
| row_40 | FLNB(ENSG00000136068) | 2946 | 0.653514504 | 0.33561343 | **Yes** |
| row_41 | IL1A(ENSG00000115008) | 3059 | 0.643043518 | 0.33866635 | **Yes** |
| row_42 | MAP4K2(ENSG00000168067) | 3392 | 0.606084228 | 0.33766967 | **Yes** |
| row_43 | DUSP2(ENSG00000158050) | 3411 | 0.603469193 | 0.342025 | **Yes** |
| row_44 | PRKACA(ENSG00000072062) | 3414 | 0.603068054 | 0.346651 | **Yes** |
| row_45 | FLNC(ENSG00000128591) | 3416 | 0.603004456 | 0.3512936 | **Yes** |
| row_46 | NGFR(ENSG00000064300) | 3452 | 0.600467861 | 0.35533488 | **Yes** |
| row_47 | PLA2G4A(ENSG00000116711) | 3486 | 0.596505523 | 0.35937977 | **Yes** |
| row_48 | MAP3K2(ENSG00000169967) | 3604 | 0.586946428 | 0.36191362 | **Yes** |
| row_49 | CRKL(ENSG00000099942) | 3696 | 0.578043401 | 0.36482355 | **Yes** |
| row_50 | EREG(ENSG00000124882) | 3738 | 0.573560715 | 0.36855426 | **Yes** |
| row_51 | RAP1A(ENSG00000116473) | 3804 | 0.567110658 | 0.3718245 | **Yes** |
| row_52 | PPM1B(ENSG00000138032) | 3862 | 0.560220659 | 0.3751784 | **Yes** |
| row_53 | RAC2(ENSG00000128340) | 3989 | 0.546748579 | 0.37724766 | **Yes** |
| row_54 | FGF22(ENSG00000070388) | 4033 | 0.542766929 | 0.3807062 | **Yes** |
| row_55 | RASGRP3(ENSG00000152689) | 4056 | 0.541062295 | 0.38451084 | **Yes** |
| row_56 | NF1(ENSG00000196712) | 4060 | 0.540413737 | 0.38863555 | **Yes** |
| row_57 | DUSP10(ENSG00000143507) | 4145 | 0.533577919 | 0.3913216 | **Yes** |
| row_58 | MAP2K4(ENSG00000065559) | 4199 | 0.529664934 | 0.3945078 | **Yes** |
| row_59 | DUSP4(ENSG00000120875) | 4360 | 0.517957568 | 0.39577287 | **Yes** |
| row_60 | TGFBR2(ENSG00000163513) | 4380 | 0.516137838 | 0.39943627 | **Yes** |
| row_61 | MAPKAPK3(ENSG00000114738 | 4381 | 0.516070902 | 0.40342417 | **Yes** |
| row_62 | PPP3CB(ENSG00000107758) | 4492 | 0.508018434 | 0.4054679 | **Yes** |
| row_63 | IL1R1(ENSG00000115594) | 4509 | 0.506626725 | 0.40910912 | **Yes** |
| row_64 | PLA2G4F(ENSG00000168907) | 5135 | 0.46642971 | 0.40202036 | No |
| row_65 | CDC25B(ENSG00000101224) | 5268 | 0.457941651 | 0.4033007 | No |
| row_66 | CHUK(ENSG00000213341) | 5532 | 0.443138927 | 0.4022254 | No |
| row_67 | TAOK3(ENSG00000135090) | 5776 | 0.431453437 | 0.40140197 | No |
| NAME | PROBE | RANK IN GENE | RANK METRIC SCORERUNNING ES CORE | | |
| row_68 | GADD45G(ENSG00000130222) | 5961 | 0.423276752 | 0.4015248 | No |
| row_69 | MAP3K8(ENSG00000107968) | 5979 | 0.422527701 | 0.40449902 | No |

| row_70 | MAP3K3(ENSG00000198909) | | 6108 | 0.415491909 | 0.40551978 | No |
| --- | --- | --- | --- | --- | --- | --- |
| row_71 | CACNG7(ENSG00000105605) | | 6466 | 0.396928102 | 0.40247914 | No |
| row_72 | EGF(ENSG00000138798) | | 6545 | 0.391661376 | 0.4041712 | No |
| row_73 | PDGFB(ENSG00000100311) | | 6650 | 0.385546774 | 0.4053712 | No |
| row_74 | FGFR2(ENSG00000066468) | | 6747 | 0.380419195 | 0.4066684 | No |
| row_75 | RASA1(ENSG00000145715) | | 6858 | 0.375424474 | 0.40768752 | No |
| row_76 | IKBKG(ENSG00000269335) | | 7142 | 0.360836416 | 0.40563405 | No |
| row_77 | FGF3(ENSG00000186895) | | 7260 | 0.353728801 | 0.40636572 | No |
| row_78 | MAP3K20(ENSG00000091436) | | 7376 | 0.348123074 | 0.4070883 | No |
| row_79 | STK3(ENSG00000104375) | | 7632 | 0.335661322 | 0.40531933 | No |
| row_80 | TGFA(ENSG00000163235) | | 7806 | 0.326946676 | 0.40488598 | No |
| row_81 | MAPK8(ENSG00000107643) | | 8127 | 0.312180191 | 0.4018235 | No |
| row_82 | RASGRP2(ENSG00000068831) | | 8414 | 0.300594002 | 0.39925316 | No |
| row_83 | MKNK1(ENSG00000079277) | | 8503 | 0.297760725 | 0.40004852 | No |
| row_84 | ELK4(ENSG00000158711) | | 8661 | 0.291600913 | 0.39961573 | No |
| row_85 | TGFB1(ENSG00000105329) | | 8694 | 0.290412635 | 0.4013124 | No |
| row_86 | PDGFRA(ENSG00000134853) | | 8731 | 0.288950384 | 0.40292937 | No |
| row_87 | GRB2(ENSG00000177885) | | 8830 | 0.285406321 | 0.40345815 | No |
| row_88 | MAPK8IP1(ENSG00000121653) | | 8934 | 0.280890346 | 0.4038665 | No |
| row_89 | ECSIT(ENSG00000130159) | | 8971 | 0.280146271 | 0.40541542 | No |
| row_90 | PPM1A(ENSG00000100614) | | 9143 | 0.271952331 | 0.4045913 | No |
| row_91 | MAP4K1(ENSG00000104814) | | 9413 | 0.258937657 | 0.4019899 | No |
| row_92 | FGF2(ENSG00000138685) | | 9582 | 0.250748277 | 0.40105328 | No |
| row_93 | ELK1(ENSG00000126767) | | 9876 | 0.238260269 | 0.3978815 | No |
| row_94 | LAMTOR3(ENSG00000109270) | | 9954 | 0.235396594 | 0.39838314 | No |
| row_95 | MAPK8IP2(ENSG00000008735) | | 9957 | 0.235256255 | 0.40016687 | No |
| row_96 | RASGRP4(ENSG00000171777) | | 10098 | 0.228971139 | 0.39954096 | No |
| row_97 | MAPKAPK2(ENSG00000162889 | | 10104 | 0.228699833 | 0.4012227 | No |
| row_98 | BRAF(ENSG00000157764) | | 10400 | 0.219891161 | 0.39787477 | No |
| row_99 | CACNB2(ENSG00000165995) | | 10553 | 0.213765889 | 0.39692608 | No |
| row_100 | IRAK4(ENSG00000198001) | | 10566 | 0.213337213 | 0.39836934 | No |
| row_101 | MAPK3(ENSG00000102882) | | 10596 | 0.212570921 | 0.3995158 | No |
| row_102 | RASGRF2(ENSG00000113319) | | 10723 | 0.209155932 | 0.39897633 | No |
| row_103 | MAPK9(ENSG00000050748) | | 10789 | 0.207512349 | 0.3994678 | No |
| row_104 | DUSP16(ENSG00000111266) | | 10927 | 0.203150898 | 0.3986937 | No |
| row_105 | RASA2(ENSG00000155903) | | 11233 | 0.194426849 | 0.39497793 | No |
| row_106 | RAC3(ENSG00000169750) | | 11524 | 0.185613945 | 0.39145067 | No |
| row_107 | MAP2K1(ENSG00000169032) | | 11540 | 0.185062498 | 0.3926241 | No |
| row_108 | RPS6KA3(ENSG00000177189) | | 11955 | 0.171613887 | 0.38686714 | No |
| row_109 | PPP3R2(ENSG00000188386) | | 12252 | 0.161954641 | 0.3830544 | No |
| row_110 | VEGFD(ENSG00000165197) | | 12633 | 0.148936316 | 0.3777039 | No |
| row_111 | RELA(ENSG00000173039) | | 12689 | 0.147285223 | 0.37790105 | No |
| row_112 | MAPK8IP3(ENSG00000138834) | | 12690 | 0.147275224 | 0.37903914 | No |
| row_113 | KIT(ENSG00000157404) | | 12721 | 0.146349132 | 0.37965676 | No |
| row_114 | RRAS2(ENSG00000133818) | | 12780 | 0.144648522 | 0.37978223 | No |
| row_115 | TNFRSF1A(ENSG00000067182) | | 12830 | 0.143148258 | 0.38005006 | No |
| row_116 | MAP3K7(ENSG00000135341) | | 12934 | 0.140075251 | 0.37937027 | No |
| row_117 | TGFB3(ENSG00000119699) | | 12986 | 0.138494805 | 0.37956792 | No |
| row_118 | TEK(ENSG00000120156) | | 13079 | 0.135761693 | 0.379043 | No |
| row_119 | ARAF(ENSG00000078061) | | 13081 | 0.135689959 | 0.38007444 | No |
| row_120 | TAB2(ENSG00000055208) | | 13175 | 0.132647291 | 0.37950832 | No |
| row_121 | MAP2K5(ENSG00000137764) | | 13266 | 0.129865408 | 0.37897205 | No |
| row_122 | SRF(ENSG00000112658) | | 13583 | 0.118870385 | 0.3744842 | No |
| row_123 | NRAS(ENSG00000213281) | | 13591 | 0.11871241 | 0.37528178 | No |
| row_124 | MAPK11(ENSG00000185386) | | 13614 | 0.117921986 | 0.37581664 | No |
| row_125 | TAOK2(ENSG00000149930) | | 13635 | 0.117212065 | 0.3763802 | No |
| row_126 | MAP4K3(ENSG00000011566) | | 13637 | 0.117147766 | 0.37726834 | No |
| row_127 | RPS6KA1(ENSG00000117676) | | 13708 | 0.11505235 | 0.3769598 | No |
| row_128 | MAPK1(ENSG00000100030) | | 13725 | 0.114244364 | 0.37756887 | No |
| row_129 | CRK(ENSG00000167193) | | 13775 | 0.112831354 | 0.37760243 | No |
| row_130 | KRAS(ENSG00000133703) | | 13777 | 0.112815574 | 0.3784571 | No |
| row_131 | RAC1(ENSG00000136238) | | 14160 | 0.100624509 | 0.37269908 | No |
| row_132 | DUSP3(ENSG00000108861) | | 14520 | 0.089227587 | 0.36724648 | No |
| row_133 | PGF(ENSG00000119630) | | 14602 | 0.086629748 | 0.36653006 | No |
| row_134 | NFATC3(ENSG00000072736) | | 14975 | 0.07327076 | 0.36073175 | No |
| row_135 | PAK1(ENSG00000149269) | | 15275 | 0.06266208 | 0.35610038 | No |
| row_136 | MAP2K2(ENSG00000126934) | | 15345 | 0.059945211 | 0.3553831 | No |
| row_137 | MET(ENSG00000105976) | | 15379 | 0.05888354 | 0.35527354 | No |
| NAME | PROBE | RANK IN GENE RANK METRIC SCORE RUNNING ES CORE | | | | |
| row_138 | RPS6KA4(ENSG00000162302) | | 15396 | 0.058403272 | 0.3554511 | No |
| row_139 | MAP2K7(ENSG00000076984) | | 15419 | 0.057731129 | 0.3555208 | No |
| row_140 | ARRB2(ENSG00000141480) | | 15511 | 0.05416356 | 0.35438246 | No |

| row_141 | DUSP7(ENSG00000164086) | | 15586 | 0.05129642 | 0.3535128 | No |
| --- | --- | --- | --- | --- | --- | --- |
| row_142 | FGFR4(ENSG00000160867) | | 15791 | 0.043262042 | 0.35035688 | No |
| row_143 | VEGFC(ENSG00000150630) | | 15881 | 0.04031973 | 0.34914574 | No |
| row_144 | MAPT(ENSG00000186868) | | 16178 | 0.030147478 | 0.34431446 | No |
| row_145 | RAPGEF2(ENSG00000109756) | | 16225 | 0.029000826 | 0.34375155 | No |
| row_146 | PDGFD(ENSG00000170962) | | 16394 | 0.024782941 | 0.34106877 | No |
| row_147 | DUSP9(ENSG00000130829) | | 16737 | 0.01908653 | 0.335365 | No |
| row_148 | FGF23(ENSG00000118972) | | 16944 | 0.016649935 | 0.33196923 | No |
| row_149 | NGF(ENSG00000134259) | | 18941 | 0 | 0.2978198 | No |
| row_150 | FASLG(ENSG00000117560) | | 19404 | 0 | 0.28991547 | No |
| row_151 | FGF5(ENSG00000138675) | | 23358 | 0 | 0.22228386 | No |
| row_152 | FGF16(ENSG00000196468) | | 28207 | 0 | 0.13933976 | No |
| row_153 | RPS6KA6(ENSG00000072133) | | 28245 | 0 | 0.13870673 | No |
| row_154 | FGF20(ENSG00000078579) | | 29103 | 0 | 0.12404437 | No |
| row_155 | ANGPT1(ENSG00000154188) | | 29770 | 0 | 0.11264982 | No |
| row_156 | PRKACG(ENSG00000165059) | | 30365 | 0 | 0.10248711 | No |
| row_157 | IGF2(ENSG00000284779) | | 30824 | 0 | 0.09465122 | No |
| row_158 | INS(ENSG00000254647) | | 30827 | 0 | 0.094617 | No |
| row_159 | FGF4(ENSG00000075388) | | 31632 | 0 | 0.08086142 | No |
| row_160 | IGF1(ENSG00000017427) | | 33884 | 0 | 0.04234922 | No |
| row_161 | FLT1(ENSG00000102755) | | 34280 | 0 | 0.03559119 | No |
| row_162 | PRKCB(ENSG00000166501) | | 37049 | 0 | -0.01176634 | No |
| row_163 | CACNG3(ENSG00000006116) | | 37055 | 0 | -0.01185188 | No |
| row_164 | CACNG5(ENSG00000075429) | | 38469 | 0 | -0.0360268 | No |
| row_165 | CACNG4(ENSG00000075461) | | 38471 | 0 | -0.03604391 | No |
| row_166 | CACNG2(ENSG00000166862) | | 41576 | 0 | -0.08915004 | No |
| row_167 | PDGFRB(ENSG00000113721) | | 42295 | -0.006304791 | -0.10138553 | No |
| row_168 | FGF7(ENSG00000140285) | | 42424 | -0.011095292 | -0.10348973 | No |
| row_169 | INSR(ENSG00000171105) | | 42482 | -0.01134769 | -0.10437726 | No |
| row_170 | FGFR1(ENSG00000077782) | | 42612 | -0.017431123 | -0.10644961 | No |
| row_171 | MAP3K4(ENSG00000085511) | | 42633 | -0.018679708 | -0.10664744 | No |
| row_172 | HGF(ENSG00000019991) | | 42669 | -0.020199411 | -0.10709016 | No |
| row_173 | TRAF2(ENSG00000127191) | | 42940 | -0.030968804 | -0.11147027 | No |
| row_174 | SOS2(ENSG00000100485) | | 43192 | -0.041102048 | -0.11544699 | No |
| row_175 | MAPK10(ENSG00000109339) | | 43225 | -0.042362932 | -0.11566712 | No |
| row_176 | RAP1B(ENSG00000127314) | | 43290 | -0.044606313 | -0.11641739 | No |
| row_177 | CACNA2D2(ENSG00000007402 | | 43425 | -0.049063366 | -0.11833086 | No |
| row_178 | MAP2K6(ENSG00000108984) | | 43610 | -0.05538268 | -0.12105093 | No |
| row_179 | FGF10(ENSG00000070193) | | 43755 | -0.061031222 | -0.123043 | No |
| row_180 | CACNA1S(ENSG00000081248) | | 43953 | -0.068856142 | -0.12588137 | No |
| row_181 | FGF6(ENSG00000111241) | | 44126 | -0.075346395 | -0.12824188 | No |
| row_182 | CACNA1I(ENSG00000100346) | | 44153 | -0.076702274 | -0.12809399 | No |
| row_183 | CACNA1H(ENSG00000196557) | | 44247 | -0.08034341 | -0.12906428 | No |
| row_184 | AKT1(ENSG00000142208) | | 44277 | -0.081308924 | -0.12893212 | No |
| row_185 | ATF2(ENSG00000115966) | | 44328 | -0.082715452 | -0.12914838 | No |
| row_186 | MAP3K1(ENSG00000095015) | | 44370 | -0.083903469 | -0.12920149 | No |
| row_187 | TRADD(ENSG00000102871) | | 44611 | -0.092590891 | -0.13259214 | No |
| row_188 | CASP3(ENSG00000164305) | | 44884 | -0.101084359 | -0.13646464 | No |
| row_189 | PTPN5(ENSG00000110786) | | 45024 | -0.105579399 | -0.13802692 | No |
| row_190 | SOS1(ENSG00000115904) | | 45146 | -0.110290058 | -0.13924484 | No |
| row_191 | GNG12(ENSG00000172380) | | 45303 | -0.115156814 | -0.14102395 | No |
| row_192 | RPS6KA2(ENSG00000071242) | | 45336 | -0.11610359 | -0.14067425 | No |
| row_193 | PPP3R1(ENSG00000221823) | | 45559 | -0.124328963 | -0.14351168 | No |
| row_194 | TAOK1(ENSG00000160551) | | 45582 | -0.124979518 | -0.14292231 | No |
| row_195 | EFNA3(ENSG00000143590) | | 45860 | -0.135092303 | -0.14661756 | No |
| row_196 | CACNG6(ENSG00000130433) | | 46184 | -0.145504802 | -0.15101936 | No |
| row_197 | HSPB1(ENSG00000106211) | | 46296 | -0.149391115 | -0.15176404 | No |
| row_198 | FGF18(ENSG00000156427) | | 46314 | -0.14997077 | -0.15089598 | No |
| row_199 | MAPK7(ENSG00000166484) | | 46320 | -0.150188699 | -0.14982095 | No |
| row_200 | AKT2(ENSG00000105221) | | 46614 | -0.161557183 | -0.15358543 | No |
| row_201 | PPP5C(ENSG00000011485) | | 47202 | -0.180259362 | -0.16223544 | No |
| row_202 | PTPN7(ENSG00000143851) | | 47215 | -0.180626348 | -0.16104496 | No |
| row_203 | CACNA1D(ENSG00000157388) | | 47315 | -0.182556242 | -0.16132805 | No |
| row_204 | MAPK14(ENSG00000112062) | | 47316 | -0.182634369 | -0.15991674 | No |
| row_205 | FGF1(ENSG00000113578) | | 47378 | -0.184685975 | -0.15953323 | No |
| row_206 | PLA2G4B(ENSG00000243708) | | 47430 | -0.186731726 | -0.15896282 | No |
| row_207 | GNA12(ENSG00000146535) | | 47575 | -0.190449968 | -0.1599548 | No |
| NAME | PROBE | RANK IN GENE RANK METRIC SCORE RUNNING ES CORE | | | | |
| row_208 | PLA2G4E(ENSG00000188089) | | 47661 | -0.192351699 | -0.15992267 | No |
| row_209 | MEF2C(ENSG00000081189) | | 48004 | -0.204709515 | -0.16419204 | No |
| row_210 | FLT4(ENSG00000037280) | | 48021 | -0.205246612 | -0.16287974 | No |
| row_211 | MAP4K4(ENSG00000071054) | | 48092 | -0.207990691 | -0.16247012 | No |

| row_212 | RASGRP1(ENSG00000172575) | 48369 | -0.218261093 | -0.16550557 | No |
| --- | --- | --- | --- | --- | --- |
| row_213 | PAK2(ENSG00000180370) | 48494 | -0.222922176 | -0.16590446 | No |
| row_214 | TAB1(ENSG00000100324) | 48542 | -0.224492386 | -0.16497383 | No |
| row_215 | STMN1(ENSG00000117632) | 48802 | -0.234767631 | -0.16759087 | No |
| row_216 | EGFR(ENSG00000146648) | 48850 | -0.236109778 | -0.16657045 | No |
| row_217 | NLK(ENSG00000087095) | 48938 | -0.239961654 | -0.16620463 | No |
| row_218 | JUND(ENSG00000130522) | 50064 | -0.283971876 | -0.1832578 | No |
| row_219 | CACNG1(ENSG00000108878) | 50180 | -0.288399726 | -0.18299672 | No |
| row_220 | HSPA2(ENSG00000126803) | 50631 | -0.30812487 | -0.18831472 | No |
| row_221 | MAP3K12(ENSG00000139625) | 50632 | -0.308152199 | -0.18593347 | No |
| row_222 | MAX(ENSG00000125952) | 50992 | -0.326742917 | -0.18955068 | No |
| row_223 | CACNA1F(ENSG00000102001) | 51008 | -0.32821852 | -0.18727101 | No |
| row_224 | HRAS(ENSG00000174775) | 51009 | -0.328227401 | -0.18473464 | No |
| row_225 | CACNG8(ENSG00000142408) | 51203 | -0.338351279 | -0.18542206 | No |
| row_226 | TRAF6(ENSG00000175104) | 51231 | -0.339957595 | -0.183257 | No |
| row_227 | TGFBR1(ENSG00000106799) | 51413 | -0.348990649 | -0.1836569 | No |
| row_228 | MAPK13(ENSG00000156711) | 51602 | -0.358973354 | -0.18409942 | No |
| row_229 | MAP3K11(ENSG00000173327) | 51618 | -0.359800786 | -0.1815757 | No |
| row_230 | PRKCG(ENSG00000126583) | 51801 | -0.368170857 | -0.18184449 | No |
| row_231 | PDGFC(ENSG00000145431) | 51857 | -0.370819479 | -0.17991999 | No |
| row_232 | CACNA2D4(ENSG00000151062 | 51928 | -0.373876512 | -0.17822848 | No |
| row_233 | CACNB3(ENSG00000167535) | 52163 | -0.387372881 | -0.17923856 | No |
| row_234 | FAS(ENSG00000026103) | 52189 | -0.389169335 | -0.17665899 | No |
| row_235 | FLT3LG(ENSG00000090554) | 52262 | -0.39348948 | -0.17485015 | No |
| row_236 | RAF1(ENSG00000132155) | 52476 | -0.403792024 | -0.17537406 | No |
| row_237 | IGF1R(ENSG00000140443) | 52511 | -0.404940516 | -0.17282659 | No |
| row_238 | IKBKB(ENSG00000104365) | 52855 | -0.42117244 | -0.17544036 | No |
| row_239 | HSPA6(ENSG00000173110) | 53035 | -0.430666476 | -0.17517489 | No |
| row_240 | FGFR3(ENSG00000068078) | 53215 | -0.44135803 | -0.1748268 | No |
| row_241 | NFATC1(ENSG00000131196) | 53216 | -0.441499174 | -0.17141512 | No |
| row_242 | PPP3CA(ENSG00000138814) | 53373 | -0.450058013 | -0.1706063 | No |
| row_243 | IL1B(ENSG00000125538) | 53498 | -0.457649946 | -0.16919133 | No |
| row_244 | NTF4(ENSG00000225950) | 53507 | -0.458129317 | -0.16578802 | No |
| row_245 | CACNB1(ENSG00000067191) | 53570 | -0.461133242 | -0.16328537 | No |
| row_246 | IRAK1(ENSG00000184216) | 53646 | -0.465725929 | -0.16096966 | No |
| row_247 | CACNA2D3(ENSG00000157445 | 53664 | -0.466618568 | -0.15765473 | No |
| row_248 | MRAS(ENSG00000158186) | 54101 | -0.495650351 | -0.16128409 | No |
| row_249 | MAP3K6(ENSG00000142733) | 54108 | -0.496239513 | -0.15755206 | No |
| row_250 | NFKB1(ENSG00000109320) | 54252 | -0.508792341 | -0.15606697 | No |
| row_251 | MAPK12(ENSG00000188130) | 54307 | -0.514080822 | -0.1530183 | No |
| row_252 | MECOM(ENSG00000085276) | 54319 | -0.51520884 | -0.14922523 | No |
| row_253 | PPP5D1(ENSG00000230510) | 54365 | -0.519112468 | -0.1459837 | No |
| row_254 | VEGFB(ENSG00000173511) | 54765 | -0.555206656 | -0.14851981 | No |
| row_255 | EFNA4(ENSG00000243364) | 55172 | -0.595752239 | -0.15086238 | No |
| row_256 | ARRB1(ENSG00000137486) | 55304 | -0.612426281 | -0.14837115 | No |
| row_257 | CACNA1C(ENSG00000151067) | 55756 | -0.665191412 | -0.15094702 | No |
| row_258 | MAP3K5(ENSG00000197442) | 55812 | -0.672025323 | -0.14669494 | No |
| row_259 | MYC(ENSG00000136997) | 55873 | -0.679797113 | -0.14246836 | No |
| row_260 | ERBB2(ENSG00000141736) | 55944 | -0.691457093 | -0.13832277 | No |
| row_261 | ERBB3(ENSG00000065361) | 55950 | -0.692493677 | -0.13305709 | No |
| row_262 | PTPRR(ENSG00000153233) | 56293 | -0.739913285 | -0.13319068 | No |
| row_263 | CACNA1E(ENSG00000198216) | 56404 | -0.757882297 | -0.12921615 | No |
| row_264 | EFNA1(ENSG00000169242) | 56549 | -0.779343605 | -0.12565747 | No |
| row_265 | RASGRF1(ENSG00000058335) | 56579 | -0.783760309 | -0.12009714 | No |
| row_266 | MAPKAPK5(ENSG00000089022 | 56638 | -0.793881893 | -0.11495475 | No |
| row_267 | EFNA2(ENSG00000099617) | 56771 | -0.818982959 | -0.11088446 | No |
| row_268 | NTF3(ENSG00000185652) | 56823 | -0.828593493 | -0.10535407 | No |
| row_269 | DUSP6(ENSG00000139318) | 56893 | -0.841843426 | -0.10002926 | No |
| row_270 | KITLG(ENSG00000049130) | 56909 | -0.845123112 | -0.09375522 | No |
| row_271 | FGF17(ENSG00000158815) | 56923 | -0.847244203 | -0.08743058 | No |
| row_272 | CSF1(ENSG00000184371) | 56988 | -0.859138072 | -0.08188658 | No |
| row_273 | HSPA8(ENSG00000109971) | 57083 | -0.878072441 | -0.07670954 | No |
| row_274 | TP53(ENSG00000141510) | 57092 | -0.88005358 | -0.07004581 | No |
| row_275 | PLA2G4D(ENSG00000159337) | 57187 | -0.895898938 | -0.06473101 | No |
| row_276 | FGF19(ENSG00000162344) | 57250 | -0.911354303 | -0.0587493 | No |
| row_277 | CD14(ENSG00000170458) | 57373 | -0.941619158 | -0.05356025 | No |
| NAME | PROBE | RANK IN GENE RANK METRIC SCORE RUNNING ES CORE | | | |
| row_278 | CACNA1A(ENSG00000141837) | 57416 | -0.954380453 | -0.04690386 | No |
| row_279 | BDNF(ENSG00000176697) | 57626 | -1.027413011 | -0.04254032 | No |
| row_280 | TGFB2(ENSG00000092969) | 57681 | -1.043916225 | -0.03539736 | No |
| row_281 | MYD88(ENSG00000172936) | 57896 | -1.113399386 | -0.03045491 | No |
| row_282 | CACNA1B(ENSG00000148408) | 57925 | -1.127147794 | -0.02222395 | No |

| row_283 | PRKACB(ENSG00000142875) | 58056 | -1.195106626 | -0.01521295 | No |
| --- | --- | --- | --- | --- | --- |
| row_284 | FGF9(ENSG00000102678) | 58141 | -1.250983596 | -0.00698315 | No |
| row_285 | ANGPT2(ENSG00000091879) | 58689 | -2.214369059 | 7.70E-04 | No |
