# Supplementary material for: CEMIP, acting as a scaffold protein for bridging GRAF1 and MIB1, promotes colorectal cancer metastasis via activating CDC42/MAPK pathway
Source: Cell Death Dis. 2023 Feb 27;14(2):167. doi: 10.1038/s41419-023-05644-z (PMC9971195; doi:10.1038/s41419-023-05644-z)
Supplement: Supplementary file 4 — Supplementary methods and materials [file 41419_2023_5644_MOESM4_ESM.docx]

**Supplementary methods and materials**

**Plasmids and reagents**

Flag-GRAF1 (WT, amino acids 1-759), Flag-GRAF1 (△SH3, amino acids 1-693) were cloned into the pcDNA3.1 vector by JTS scientific (Wuhan, China). CEMIP-myc (amino acids 1-303, amino acids 295-591, amino acids 572-819, amino acids 820-1204, amino acids 1205-1361 and amino acids 1-1361) was cloned into the CMV-MCS-polyA-EF1A-zsGreen-sv40-puromycin vector by Genechem Group (Shanghai, China). LV-GV358-CEMIP-puromycin and LV-GV640-GRAF1-neomycin Lentiviral vector was construct by Genechem Group (Shanghai, China). pCMV-RFP-shGRAF1-neomycin plasmid was designed and constructed by GenePharma Technologies (5’-UAAGGAAUCUGCAAACUUCCGCUUC-3’; 5’-CCACACCGUUCAGUCACAGAGAAA-3’, Suzhou, China). LV12-shCEMIP-luciferase plasmid was also designed and constructed by GenePharma Technologies and the detailed information was described in our previous research. MIB1-his plasmid was designed by Weizhen Biosciences (Shandong, China). Si-MIB1 was produced by RIBOBIO (Guangzhou, China).si-MIB1 1: 5’-GGACAAGGATAATACCAAT-3’; si-MIB1 2:5’-GAAGAAAGATGATGGTTAT-3’; si-MIB1 3: 5’-GAACGAAGAGTGCCTTTCA-3’. HA-ubiquitin overexpression plasmid was donated by Dr. Shuangbing Xu. The proteasome inhibitor MG-132 (Cat. No. S2619) and CDC42 inhibitor ZCL278 (No.S7293) were purchased from Selleckchem (Shanghai, China). The Cycloheximide (CHX) was purchased from MCE (Shanghai, China).

Antibodies used in our study: GRAF1 (Cell Signaling Technology, #8802S, working dilution for WB:1:200; Proteintech, #17747-1-AP, work for IP and working dilution for IHC, 1:100), CEMIP (Santa Cruz, #sc-293483, work for IP, IF,1:50; Proteintech, #21129-1-AP, working dilution for WB, 1:1000; IHC, 1:200), MIB1 (Proteintech, #11893-1-AP, work for IP and working dilution for WB, 1:800,IF,1:50; Santa Cruz, #sc-393551,work for IF,1:50), GAPDH (Ig, #AC002, working dilution for WB, 1:3000), CDC42 (Cell Signaling Technology, #2466T, working dilution for WB, 1:500), p-PAK1 (Cell Signaling Technology, #2606S, working dilution for WB, 1:400), PAK1 (Cell Signaling Technology, #2602S, working dilution for WB, 1:1000), p-MEK (Cell Signaling Technology, #9154T, working dilution for WB, 1:1000), MEK (Cell Signaling Technology, #8727T, working dilution for WB, 1:1000), p-ERK (Cell Signaling Technology, #4370T, working dilution for WB, 1:1000), ERK (Cell Signaling Technology, #2695T, working dilution for WB, 1:1000), Myc-tag (Abclonal, #AE070, working dilution for IP and WB, 1:1000; Proteintech, #60003-2-Ig, working dilution for I P and WB:1:2000), Flag (Sigma, #F1804, working dilution for WB, 1:1000; for IP and IF, 1:100), HA (Cell Signaling Technology, #3724S, working dilution for WB, 1:1000), HIS tag (Proteintech, #66005-1-Ig, working dilution for WB, 1:1000),E-cadherin (Proteintech, #20874-1-AP, working dilution for WB, 1:5000), N-cadherin (Proteintech, #66219-1-Ig, working dilution for WB, 1:500), Fibronectin (Proteintech, #15613-1-AP, working dilution for WB, 1:500), ZO1 (Proteintech, #21773-1-AP, working dilution for WB, 1:1000), Vimentin (Proteintech, #10366-1-AP, working dilution for WB, 1:1000), SMURF1 (Proteintech, #55175-1-AP, working dilution for WB, 1:1000), CBL (Proteintech, #25818-1-AP, working dilution for WB, 1:1000), NEDD4 (Proteintech, #21698-1-AP, working dilution for WB, 1:1000), CBLB (Proteintech,#12781-1-AP, working dilution for WB, 1:1000), IgG (Rabbit, Cell Signaling Technology, #3900,for IP) All secondary antibodies were provided by Servicebio (Wuhan, China).

**Immunohistochemistry**

Paraffin-embedded CRC specimens were collected from our hospital (n=87). Our research was approved by the institutional Medical Ethics Committee. Informed consents were obtained from all involved patients. The tissue sections were dewaxed, and endogenous peroxidase was blocked by 1% hydrogen peroxide. After incubated with primary antibody of CEMIP and GRAF1 overnight at 4°C and being washed, tissue sections were treated with biotinylated secondary antibody for 1 h at room temperature. Positive cells were visualized using a 3,30-diaminobenzidine substrate and the sections were counterstained with hematoxylin. The staining index for assessing CEMIP and GRAF1 protein expression was established based on the immunostaining intensity (no staining of cells =0; low = 1; medium = 2; high = 3) and the percentage of the positively stained area (0–25% = 1, 26–50% = 2,51–75% = 3, >75% = 4). The multiplying outcome of the staining intensity and staining percentage was 6 or greater were considered to exhibit high expression, while the outcome was less than 6 were considered low expression.

**RT-PCR analysis**

RNA was extracted by the TRIZOL reagents (R6834-01, Omega, USA). The complementary DNA (cDNA) was synthesized following the protocol of ReverTra Ace qPCR RT Kit (FSQ-101, Toyobo, Japan). The 2^ΔΔCt^ method was used for quantification and GAPDH was used for the housekeeping gene. The primers for RT-PCR were as follows:

GAPDH primer forward, 5ʹ-CCAGAACATCATCCCTGCCT-3ʹ

and reverse, 5ʹ‐CCTGCTTCACCACCTTCTTG‐3ʹ;

CEMIP primer forward, 5’-GCACCTTGGATTTAGACACCC-3’

and reverse, 5ʹ‐CGTCTTGAACCCACTCACTG‐3ʹ;

GRAF1 primer forward, 5ʹ‐AACCAGAGGGATCAACGAGC‐3ʹ;

and reverse, 5ʹ‐TTGGGGTCCATCAGGACACT‐3ʹ;

MIB1 primer forward, 5ʹ‐ATGTGCTGTGGAGGGAAAAG-3’;

and reverse, 5ʹ‐GACACACAGGGCACATTGTC‐3ʹ.

**Statistical analysis**

All experiments were repeated in three times or more. All data are presented as mean value ± standard deviation (SD). Comparisons between groups were calculated by one way or two ways ANOVA using GraphPad Prism7.0 software. Survival of patients or mice was assessed by Kaplan−Meier curve and Log-Rank test. Two-sided *P* value ≤ 0.05 was considered statistically significant. No statistical method was used for the estimation of sample size in animal experiments.
