# Supplementary material for: CEMIP, acting as a scaffold protein for bridging GRAF1 and MIB1, promotes colorectal cancer metastasis via activating CDC42/MAPK pathway
Source: Cell Death Dis. 2023 Feb 27;14(2):167. doi: 10.1038/s41419-023-05644-z (PMC9971195; doi:10.1038/s41419-023-05644-z)
Supplement: Supplementary file 5 — E3 ligase from ubibrowser database [file 41419_2023_5644_MOESM5_ESM.pdf]

| E3 | E3GENE | SUB | SUBGENE | HOMO | PFAM | GO | NET | MOTIF | SCORE |
|---|---|---|---|---|---|---|---|---|---|
| Q9HCE7 | SMURF1 | Q9UNA1 | ARHGAP26 | 1 | 3.17 | 2.88 | 1.29 | 2.12 | 0.802 |
| P22681 | CBL | Q9UNA1 | ARHGAP26 | 1 | 3.32 | 3.98 | 1.87 | 1 | 0.782 |
| Q9ULV8 | CBLC | Q9UNA1 | ARHGAP26 | 1 | 3.32 | 2.88 | 1.84 | 1 | 0.776 |
| P46934 | NEDD4 | Q9UNA1 | ARHGAP26 | 1 | 2.47 | 3.77 | 1.77 | 1 | 0.772 |
| Q13191 | CBLB | Q9UNA1 | ARHGAP26 | 1 | 3.32 | 2.88 | 1.69 | 1 | 0.77 |
| Q00987 | MDM2 | Q9UNA1 | ARHGAP26 | 1 | 2.47 | 1.51 | 1.77 | 2.12 | 0.759 |
| Q96PU5 | NEDD4L | Q9UNA1 | ARHGAP26 | 1 | 3.17 | 1.24 | 1 | 2.8 | 0.739 |
| Q5T0T0 | MARCH8 | Q9UNA1 | ARHGAP26 | 1 | 1 | 1.25 | 1 | 8.67 | 0.738 |
| Q8TCQ1 | MARCH1 | Q9UNA1 | ARHGAP26 | 1 | 1 | 1.25 | 1 | 8.67 | 0.738 |
| Q86TM6 | SYVN1 | Q9UNA1 | ARHGAP26 | 1 | 1 | 1.25 | 1.29 | 6.61 | 0.736 |
| Q86YT6 | MIB1 | Q9UNA1 | ARHGAP26 | 1 | 2.47 | 1.51 | 1 | 2.8 | 0.735 |
| Q9HAU4 | SMURF2 | Q9UNA1 | ARHGAP26 | 1 | 3.17 | 1.78 | 1.44 | 1.06 | 0.718 |
| Q9UNE7 | STUB1 | Q9UNA1 | ARHGAP26 | 1 | 1 | 3.98 | 1.77 | 1 | 0.7 |
| Q96C24 | SYTL4 | Q9UNA1 | ARHGAP26 | 1 | 3.17 | 1.51 | 1.44 | 1 | 0.698 |
| Q86T96 | RNF180 | Q9UNA1 | ARHGAP26 | 1 | 2.47 | 1.25 | 1 | 2.12 | 0.693 |
| Q15386 | UBE3C | Q9UNA1 | ARHGAP26 | 1 | 2.47 | 1.25 | 1 | 2.12 | 0.693 |
| Q7Z6Z7 | HUWE1 | Q9UNA1 | ARHGAP26 | 1 | 3.32 | 1.25 | 1.44 | 1 | 0.685 |
| Q96J02 | ITCH | Q9UNA1 | ARHGAP26 | 1 | 3.17 | 1.13 | 1.44 | 1.06 | 0.677 |
| Q9H0M0 | WWP1 | Q9UNA1 | ARHGAP26 | 1 | 3.17 | 1.25 | 1.29 | 1 | 0.67 |
| O15151 | MDM4 | Q9UNA1 | ARHGAP26 | 1 | 2.47 | 1.13 | 1.77 | 1 | 0.667 |
| Q13490 | BIRC2 | Q9UNA1 | ARHGAP26 | 1 | 2.47 | 1.25 | 1.44 | 1.06 | 0.662 |
| O95071 | UBR5 | Q9UNA1 | ARHGAP26 | 1 | 2.47 | 1.25 | 1.44 | 1 | 0.657 |
| Q05086 | UBE3A | Q9UNA1 | ARHGAP26 | 1 | 2.47 | 1.25 | 1.29 | 1.06 | 0.651 |
| Q9NWF9 | RNF216 | Q9UNA1 | ARHGAP26 | 1 | 1 | 1.13 | 1.29 | 2.8 | 0.648 |
| Q9UH77 | KLHL3 | Q9UNA1 | ARHGAP26 | 1 | 1 | 1.13 | 1.29 | 2.8 | 0.648 |
| Q13489 | BIRC3 | Q9UNA1 | ARHGAP26 | 1 | 2.47 | 1.13 | 1.44 | 1 | 0.647 |
| O00308 | WWP2 | Q9UNA1 | ARHGAP26 | 1 | 2.47 | 1.25 | 1.29 | 1 | 0.646 |
| P98170 | XIAP | Q9UNA1 | ARHGAP26 | 1 | 2.47 | 1.25 | 1.29 | 1 | 0.646 |
| O60260 | PARK2 | Q9UNA1 | ARHGAP26 | 1 | 3.17 | 1.25 | 1 | 1 | 0.645 |
| P43034 | PAFAH1B1 | Q9UNA1 | ARHGAP26 | 1 | 1 | 3.77 | 1 | 1 | 0.64 |
| Q96CA5 | BIRC7 | Q9UNA1 | ARHGAP26 | 1 | 2.47 | 1.13 | 1.29 | 1 | 0.636 |
| Q5XPI4 | RNF123 | Q9UNA1 | ARHGAP26 | 1 | 2.47 | 1.13 | 1.29 | 1 | 0.636 |
| Q96PU4 | UHRF2 | Q9UNA1 | ARHGAP26 | 1 | 3.17 | 1.13 | 1 | 1 | 0.635 |
| Q9P2P5 | HECW2 | Q9UNA1 | ARHGAP26 | 1 | 3.17 | 1.13 | 1 | 1 | 0.635 |
| Q76N89 | HECW1 | Q9UNA1 | ARHGAP26 | 1 | 3.17 | 1.13 | 1 | 1 | 0.635 |
| Q96T88 | UHRF1 | Q9UNA1 | ARHGAP26 | 1 | 3.17 | 1.13 | 1 | 1 | 0.635 |
| P53804 | TTC3 | Q9UNA1 | ARHGAP26 | 1 | 1 | 1.25 | 1 | 2.8 | 0.633 |
| Q8WY64 | MYLIP | Q9UNA1 | ARHGAP26 | 1 | 2.47 | 1.25 | 1 | 1.06 | 0.626 |
| Q9UM11 | FZR1 | Q9UNA1 | ARHGAP26 | 1 | 1 | 1.51 | 1 | 2.12 | 0.624 |
| Q9Y4X5 | ARIH1 | Q9UNA1 | ARHGAP26 | 1 | 1.73 | 1.25 | 1.44 | 1 | 0.621 |
| O76050 | NEURL1 | Q9UNA1 | ARHGAP26 | 1 | 2.47 | 1.25 | 1 | 1 | 0.62 |
| O95714 | HERC2 | Q9UNA1 | ARHGAP26 | 1 | 2.47 | 1.25 | 1 | 1 | 0.62 |
| O15033 | AREL1 | Q9UNA1 | ARHGAP26 | 1 | 2.47 | 1.25 | 1 | 1 | 0.62 |
| Q9UII4 | HERC5 | Q9UNA1 | ARHGAP26 | 1 | 2.47 | 1.25 | 1 | 1 | 0.62 |
| Q5M7Z0 | RNFT1 | Q9UNA1 | ARHGAP26 | 1 | 2.47 | 1.25 | 1 | 1 | 0.62 |
| Q969K3 | RNF34 | Q9UNA1 | ARHGAP26 | 1 | 2.47 | 1.25 | 1 | 1 | 0.62 |
| Q8WZ73 | RFFL | Q9UNA1 | ARHGAP26 | 1 | 2.47 | 1.25 | 1 | 1 | 0.62 |
| Q969V5 | MUL1 | Q9UNA1 | ARHGAP26 | 1 | 2.47 | 1.25 | 1 | 1 | 0.62 |
| Q5GLZ8 | HERC4 | Q9UNA1 | ARHGAP26 | 1 | 2.47 | 1.25 | 1 | 1 | 0.62 |
| Q7L622 | G2E3 | Q9UNA1 | ARHGAP26 | 1 | 2.47 | 1.25 | 1 | 1 | 0.62 |
| Q5T447 | HECTD3 | Q9UNA1 | ARHGAP26 | 1 | 2.47 | 1.25 | 1 | 1 | 0.62 |
| Q8IVU3 | HERC6 | Q9UNA1 | ARHGAP26 | 1 | 2.47 | 1.25 | 1 | 1 | 0.62 |
| Q6UWE0 | LRSAM1 | Q9UNA1 | ARHGAP26 | 1 | 2.47 | 1.25 | 1 | 1 | 0.62 |
| Q15751 | HERC1 | Q9UNA1 | ARHGAP26 | 1 | 2.47 | 1.25 | 1 | 1 | 0.62 |
| Q14669 | TRIP12 | Q9UNA1 | ARHGAP26 | 1 | 2.47 | 1.25 | 1 | 1 | 0.62 |
| P38398 | BRCA1 | Q9UNA1 | ARHGAP26 | 1 | 1 | 1.51 | 1.84 | 1.06 | 0.615 |
| Q86UW7 | CADPS2 | Q9UNA1 | ARHGAP26 | 1 | 1 | 2.88 | 1 | 1 | 0.613 |

| | | | | | | | | | |
|---|---|---|---|---|---|---|---|---|---|
| Q8IZP6 | RNF113B | Q9UNA1 | ARHGAP26 | 1 | 2.47 | 1.13 | 1 | 1 | 0.61 |
| Q5U5Q3 | MEX3C | Q9UNA1 | ARHGAP26 | 1 | 2.47 | 1.13 | 1 | 1 | 0.61 |
| Q5U5R9 | HECTD2 | Q9UNA1 | ARHGAP26 | 1 | 2.47 | 1.13 | 1 | 1 | 0.61 |
| Q9UJV3 | MID2 | Q9UNA1 | ARHGAP26 | 1 | 2.47 | 1.13 | 1 | 1 | 0.61 |
| Q9ULT8 | HECTD1 | Q9UNA1 | ARHGAP26 | 1 | 2.47 | 1.13 | 1 | 1 | 0.61 |
| O15344 | MID1 | Q9UNA1 | ARHGAP26 | 1 | 2.47 | 1.13 | 1 | 1 | 0.61 |
| A8MQ27 | NEURL1B | Q9UNA1 | ARHGAP26 | 1 | 2.47 | 1.13 | 1 | 1 | 0.61 |
| O60291 | MGRN1 | Q9UNA1 | ARHGAP26 | 1 | 2.47 | 1.13 | 1 | 1 | 0.61 |
| Q9H9P5 | UNKL | Q9UNA1 | ARHGAP26 | 1 | 2.47 | 1.13 | 1 | 1 | 0.61 |
| Q7Z3V4 | UBE3B | Q9UNA1 | ARHGAP26 | 1 | 2.47 | 1.13 | 1 | 1 | 0.61 |
| Q9BY78 | RNF26 | Q9UNA1 | ARHGAP26 | 1 | 2.47 | 1.13 | 1 | 1 | 0.61 |
| Q96PX1 | RNF157 | Q9UNA1 | ARHGAP26 | 1 | 2.47 | 1.13 | 1 | 1 | 0.61 |
| Q15034 | HERC3 | Q9UNA1 | ARHGAP26 | 1 | 2.47 | 1.13 | 1 | 1 | 0.61 |
| Q86XN8 | MEX3D | Q9UNA1 | ARHGAP26 | 1 | 2.47 | 1.13 | 1 | 1 | 0.61 |
| Q96AX9 | MIB2 | Q9UNA1 | ARHGAP26 | 1 | 2.47 | 1.13 | 1 | 1 | 0.61 |
| Q8IYU2 | HACE1 | Q9UNA1 | ARHGAP26 | 1 | 2.47 | 1.13 | 1 | 1 | 0.61 |
| Q8NCN4 | RNF169 | Q9UNA1 | ARHGAP26 | 1 | 2.47 | 1.13 | 1 | 1 | 0.61 |
| O14512 | SOCS7 | Q9UNA1 | ARHGAP26 | 1 | 1 | 1.51 | 1.84 | 1 | 0.609 |
| Q9BYM8 | RBCK1 | Q9UNA1 | ARHGAP26 | 1 | 1.73 | 1.13 | 1.29 | 1.06 | 0.605 |
| Q92831 | KAT2B | Q9UNA1 | ARHGAP26 | 1 | 1 | 1.51 | 1.77 | 1 | 0.605 |
