# Supplementary material for: CEMIP, acting as a scaffold protein for bridging GRAF1 and MIB1, promotes colorectal cancer metastasis via activating CDC42/MAPK pathway
Source: Cell Death Dis. 2023 Feb 27;14(2):167. doi: 10.1038/s41419-023-05644-z (PMC9971195; doi:10.1038/s41419-023-05644-z)
Supplement: Supplementary file 6 — STR report [file 41419_2023_5644_MOESM6_ESM.pdf]

细胞遗传质量鉴定检测

Cell Line Authentication Service

STR 基因型检测报告

送检单位：武汉友芝友生物制药股份有限公司

检品名称：细胞系

委托单位：上海翼和应用生物技术有限公司

报告日期：2022-08-22

报告说明

1. 本报告只对送检的来样负责。
2. 检验报告上的检验结果和检验单位名称，未经同意不得用于广告、评优及商业宣传。
3. 对本报告有异议，请于收到报告之日起十五日内以书面方式提出，逾期不予受理。
4. 对纸质检验报告涂改、增删，或未加盖检验单位印章的复印件均无效。

上海翼和应用生物技术有限公司　联系地址：上海市松江区龙腾路 1015 弄中星创意园 2 号 502 室
网址：http://www.biowing.com.cn/邮箱：market@biowing.com.cn　服务热线：021-33559491

样 品 信 息

样品编号：

客户样本编号	公司编号
YZY-001	20220818-01
YZY-002	20220818-02
YZY-003	20220818-03

样品数量：3

样品性状：细胞系

检测项目：STR

送检单位：武汉友芝友生物制药股份有限公司

检测方法：用 Axygen 的基因组抽提试剂盒提取 DNA，采用 21- STR 扩增方案扩增，在 ABI 3730XL 型遗传分析仪上对 STR 位点和性别基因 Amelogenin 进行检测。

检 测 结 果

（一） 检验基本情况

公司编号	多等位基因	匹配细胞系	细胞库	EV 值	匹配说明
20220818-01	无	HCT 116	DSMZ	1.0	完全匹配
20220818-02	无	SW-480	DSMZ	1.0	完全匹配
20220818-03	有	HEK293T	EXPASY	1.0	完全匹配

样本基因型检验结果

- 多等位基因指三等位及以上基因现象。
- 本次检测各细胞分型结果良好。

（二） 各样本描述

- 20220818-01：该株细胞 DNA 分型在细胞系检索中找到**完全匹配**的细胞系，DSMZ 数据库显示细胞名为 **HCT 116**，细胞号对应 **CCL-247**。本次检测在该细胞系中**没有发现多等位基因**。
- 20220818-02：该株细胞 DNA 分型在细胞系检索中找到**完全匹配**的细胞系，DSMZ 数据库显示细胞名为 **SW-480**，细胞号对应 **313**。本次检测在该细胞系中**没有发现多等位基因**。
- 20220818-03：该株细胞 DNA 分型在细胞系检索中找到**完全匹配**的细胞系，EXPASY 数据库显示细胞名为 **HEK293T**，细胞号对应 **CVCL_0063Best**。本次检测在该细胞系中**发现多等位基因**。

备注：待测细胞系与收录于 ATCC, DSMZ, JCRB 和 RIKEN 数据库的细胞系 STR 数据进行比对，未收录于以上细胞库的细胞系将无法匹配。

上海翼和应用生物技术有限公司 联系地址：上海市松江区龙腾路 1015 弄中星创意园 2 号 502 室
网址：http://www.biowing.com.cn/邮箱：market@biowing.com.cn 服务热线：021-33559491

（三）样本分型结果

细胞的 STR 位点和 Amelogenin 位点的基因分型结果						
Loci	送检细胞 STR 信息			细胞库细胞 STR 信息		
	送检细胞名：YZY-001			细胞库细胞名：HCT 116		
	Allele1	Allele2	Allele3	Allele1	Allele2	Allele3
D5S818	10	11		10	11	
D13S317	10	12		10	12	
D7S820	11	12		11	12	
D16S539	11	13		11	13	
VWA	17	22		17	22	
TH01	8	9		8	9	
AMEL	X	Y		X	Y	
TPOX	8	9		8	9	
CSF1PO	7	10		7	10	
D12S391	17	21				
FGA	18	23				
D2S1338	16	16				
D21S11	29	30				
D18S51	16	17				
D8S1179	12	14				
D3S1358	12	19				
D6S1043	13	13				
PENTAE	13	14				
D19S433	12	13				
PENTAD	9	13				
D1S1656	12	14				

细胞的 STR 位点和 Amelogenin 位点的基因分型结果						
Loci	送检细胞 STR 信息			细胞库细胞 STR 信息		
	送检细胞名：YZY-002			细胞库细胞名：SW-480		
	Allele1	Allele2	Allele3	Allele1	Allele2	Allele3
D5S818	13	13		13	13	
D13S317	12	12		12	12	
D7S820	8	8		8	8	
D16S539	13	13		13	13	
VWA	16	16		16	16	
TH01	8	8		8	8	
AMEL	X	X		X	X	
TPOX	11	11		11	11	
CSF1PO	13	14		13	14	
D12S391	17	17				
FGA	24	24				
D2S1338	17	24				
D21S11	30	30.2				
D18S51	13	13				
D8S1179	13	13				
D3S1358	15	15				
D6S1043	11	12				
PENTAE	10	10				
D19S433	13	13				
PENTAD	9	15				
D1S1656	13	14				

上海翼和应用生物技术有限公司 联系地址：上海市松江区龙腾路 1015 弄中星创意园 2 号 502 室
网址：http://www.biowing.com.cn/邮箱：market@biowing.com.cn 服务热线：021-33559491

细胞的 STR 位点和 Amelogenin 位点的基因分型结果						
Loci	送检细胞 STR 信息			细胞库细胞 STR 信息		
	送检细胞名：YZY-003			细胞库细胞名：HEK293T		
	Allele1	Allele2	Allele3	Allele1	Allele2	Allele3
D5S818	8	9		8	9	
D13S317	12	12		12	12	
D7S820	11	11		11	11	
D16S539	9	13		9	13	
VWA	16	19		16	19	
TH01	7	9.3		7	9.3	
AMEL	X	X		X	X	
TPOX	11	11		11	11	
CSF1PO	11	12		11	12	
D12S391	19	21				
FGA	23	23				
D2S1338	19	19				
D21S11	28	30.2				
D18S51	17	18				
D8S1179	11	12	14			
D3S1358	15	16	17			
D6S1043	11	11				
PENTAE	7	15				
D19S433	18	18				
PENTAD	9	10				
D1S1656	15	17.3				

其他说明

(一) 分型方案及位点分布

	方案 1	方案 2	方案 3	方案 4
1	D3S1358	D8S1179	D19S433	AMEL
2	VWA	D21S11	TH01	D1S1656
3	D7S820	D16S539	D13S317	D5S818
4	CSF1PO	D2S1338	TPOX	D12S391
5	PENTAE	PENTAD	D18S51	FGA
6			D6S1043	

实验方案及位点

(二) STR 数据库比对

本公司采用 DSMZ tools 进行细胞系比对，其中包含来自于 ATCC, DSMZ, JCRB 和 RIKEN 数据库的 2455 个细胞系 STR 数据。如果待检测细胞未收录于以上细胞库或这是自行建立的新细胞系将无法进行比对，用户需根据细胞分型结果自行与其他数据库进行比对。

(三) 文献引用参考

1．Authentication testing of HEK 293T and HeLa cell lines have been performed by Shanghai Biowing Applied Biotechnology Co.,Ltd via STR profiling. STR profiles match the standards rec ommended for HEK 293T and HeLa cell lines authentication

2．AGS, NCI-N87, HGC-27 and HEK293 were STR-authenticated on Dec. 8, 2015 by Shanghai Biowing Applied Biotechnology Co. LTD, Shanghai, China

主要实验人员：张佳男

复核人: 张晨茜

负责人：巢凯悦

签发日期：2022-08-22

上海翼和应用生物技术有限公司

Applied Biosystems

GeneMapper ID v3.2

20220818

Sample File	Sample Name	Panel	SQO	OS	SQ
73_A10_Cellidentification-1-0819.fsa	YZY-002	21Plex_STR_Panel_v1.1			

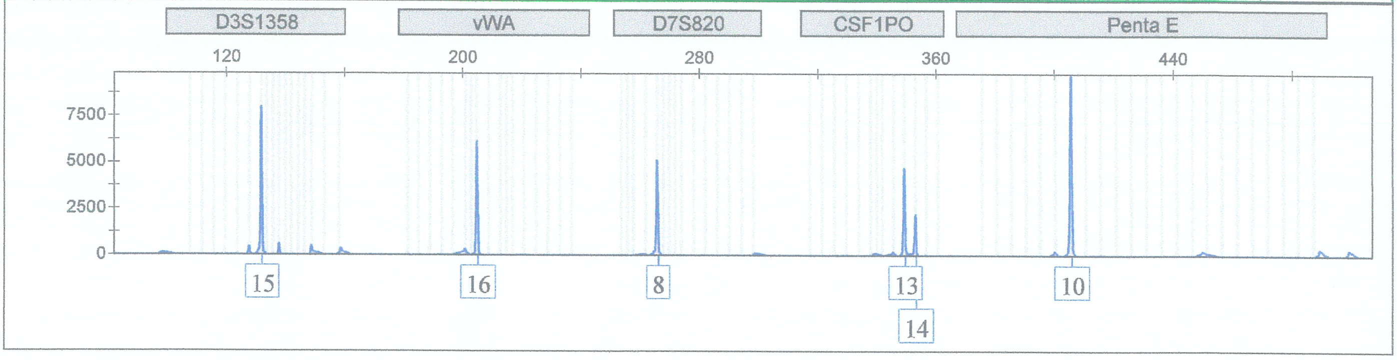

73_A10_Cellidentification-1-0819.fsa	YZY-002	21Plex_STR_Panel_v1.1			

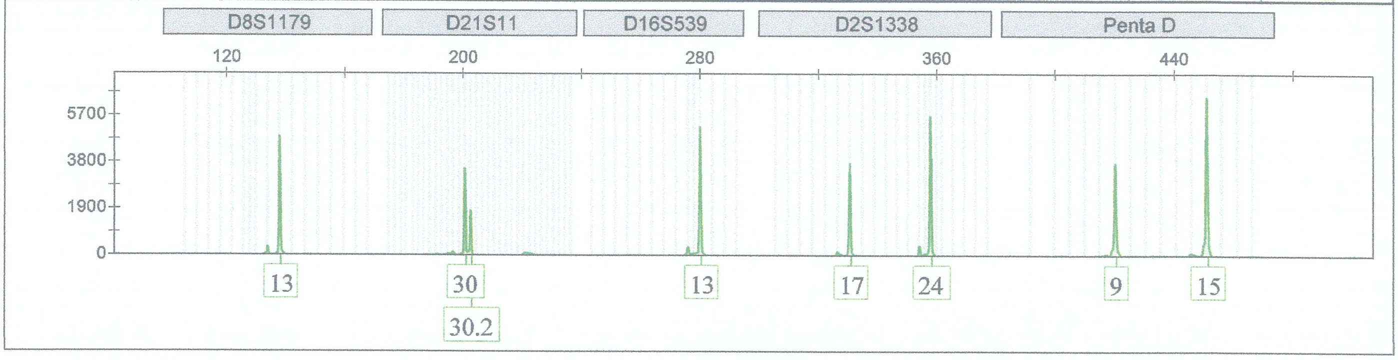

73_A10_Cellidentification-1-0819.fsa	YZY-002	21Plex_STR_Panel_v1.1			

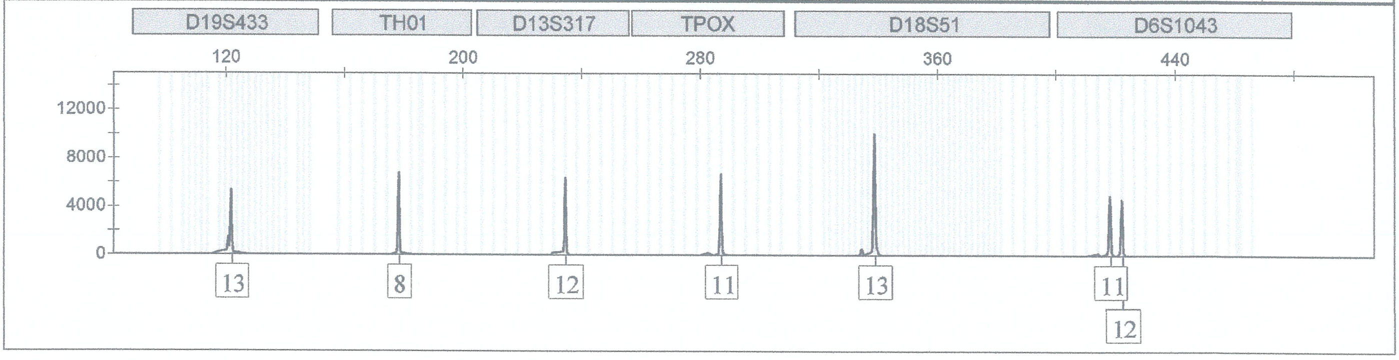

73_A10_Cellidentification-1-0819.fsa	YZY-002	21Plex_STR_Panel_v1.1			

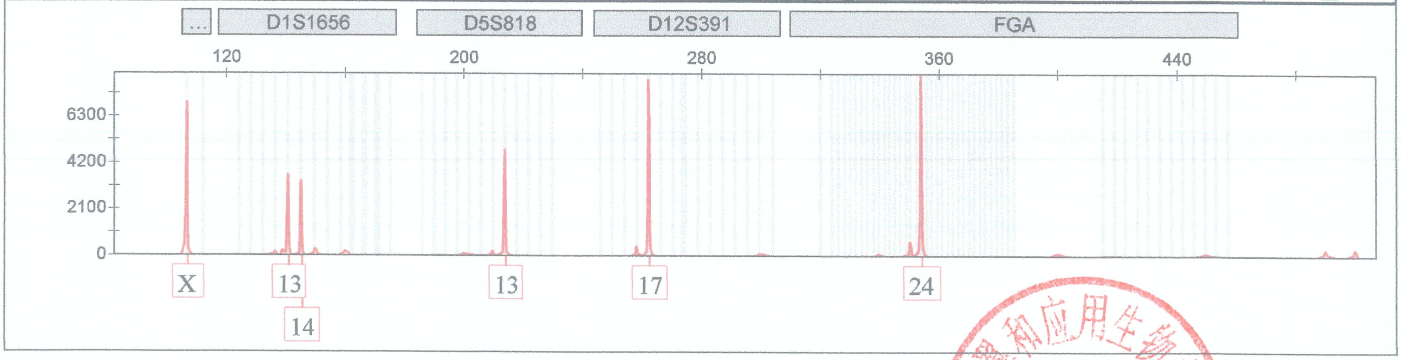

上海翼和应用生物技术有限公司

Applied Biosystems

GeneMapper ID v3.2

20220818

Sample File	Sample Name	Panel	SQO	OS	SQ
72_H09_Cellidentification-1-0819.fsa	YZY-001	21Plex_STR_Panel_v1.1			

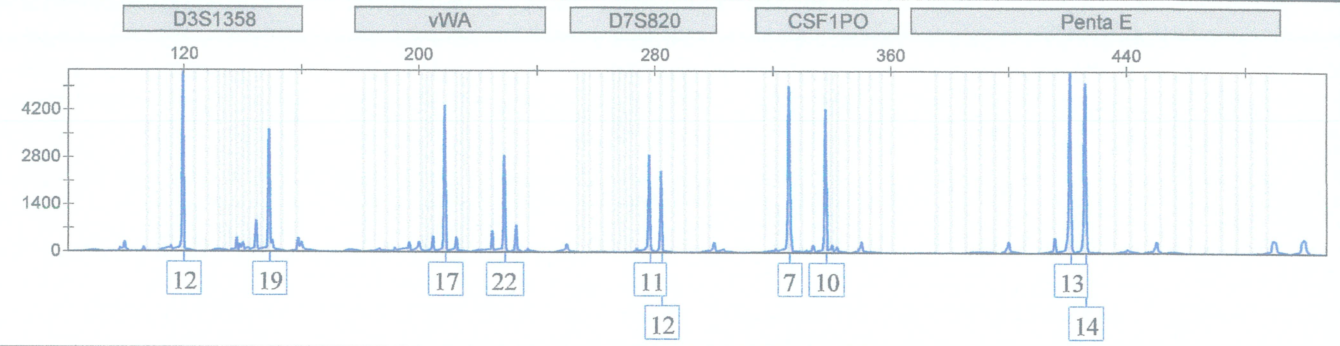

Sample File	Sample Name	Panel	SQO	OS	SQ
72_H09_Cellidentification-1-0819.fsa	YZY-001	21Plex_STR_Panel_v1.1			

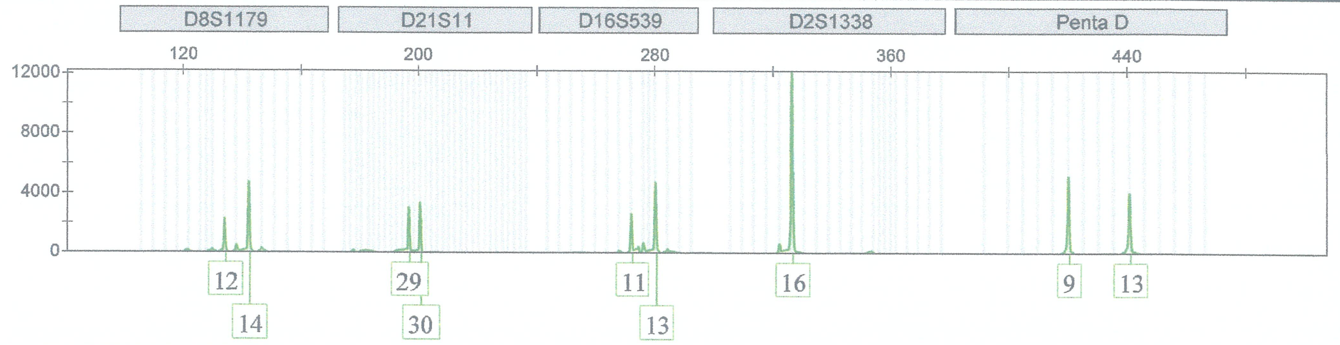

Sample File	Sample Name	Panel	SQO	OS	SQ
72_H09_Cellidentification-1-0819.fsa	YZY-001	21Plex_STR_Panel_v1.1			

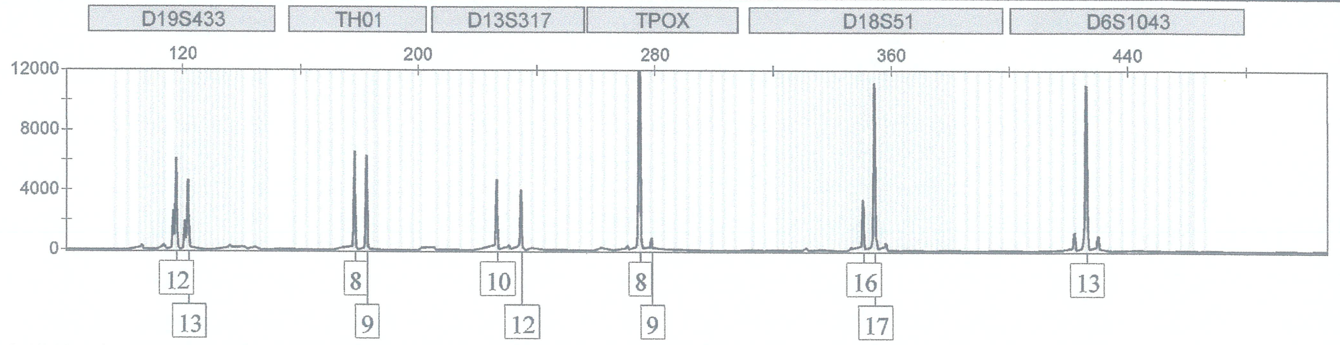

Sample File	Sample Name	Panel	SQO	OS	SQ
72_H09_Cellidentification-1-0819.fsa	YZY-001	21Plex_STR_Panel_v1.1			

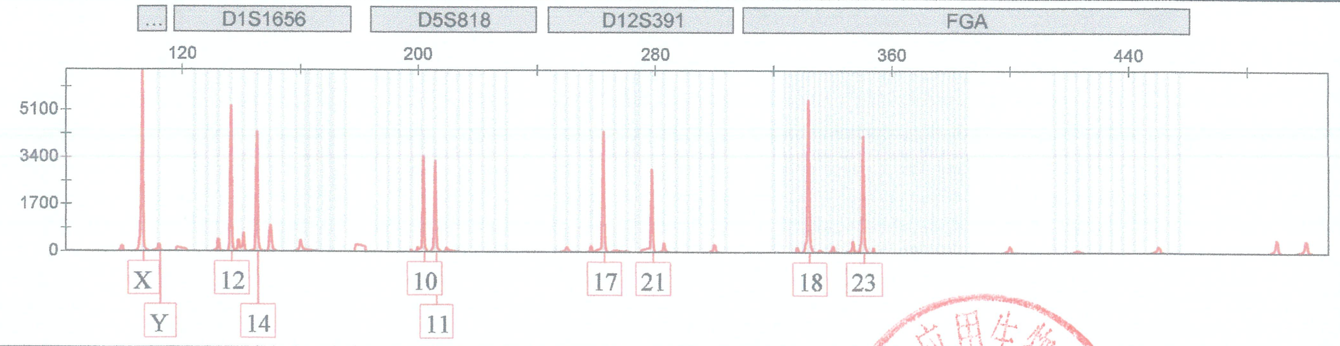

上海翼和应用生物技术有限公司

Applied Biosystems

GeneMapper ID v3.2

20220818

Sample File	Sample Name	Panel	SQO	OS	SQ
74_B10_Cellidentification-1-0819.fsa	YZY-003	21Plex_STR_Panel_v1.1			

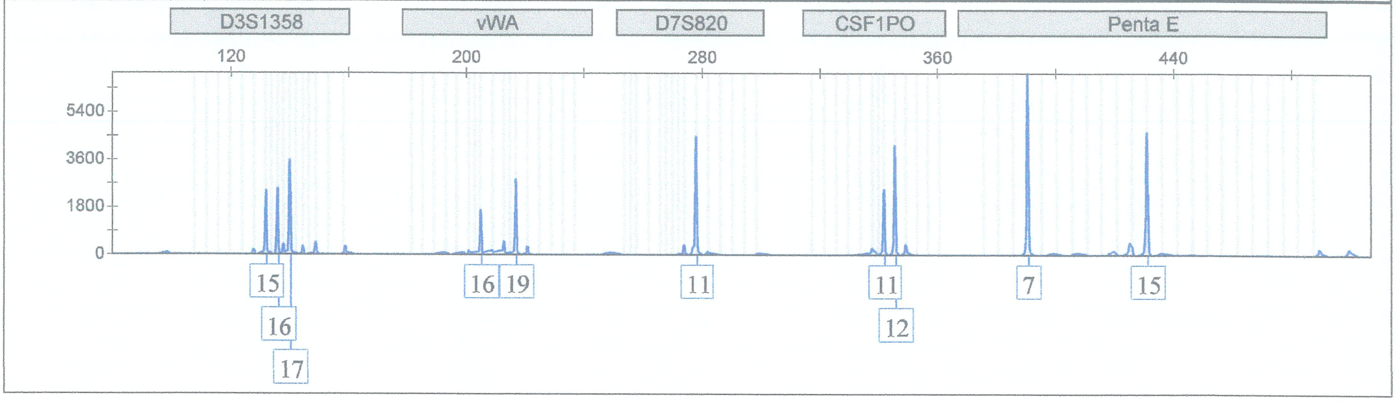

74_B10_Cellidentification-1-0819.fsa	YZY-003	21Plex_STR_Panel_v1.1			

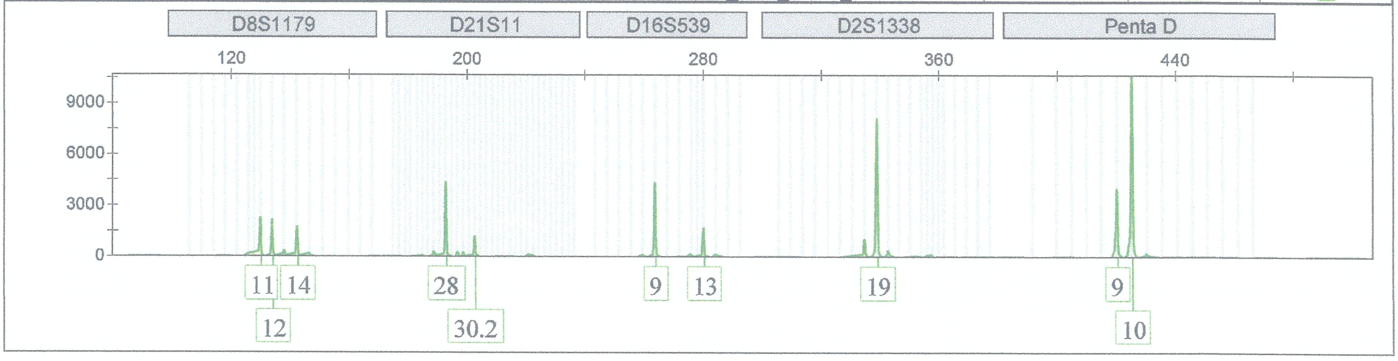

74_B10_Cellidentification-1-0819.fsa	YZY-003	21Plex_STR_Panel_v1.1			

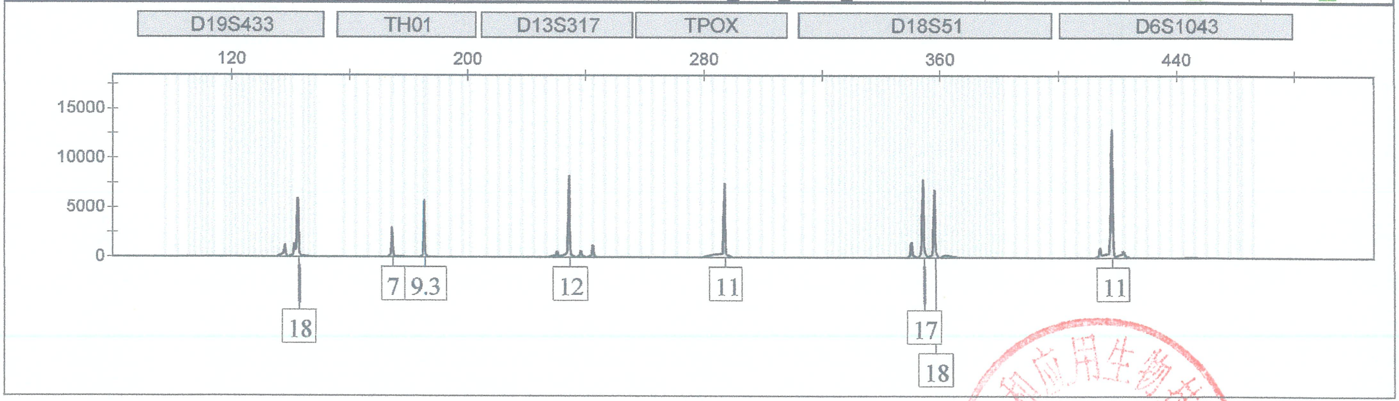

上海襄和应用生物技术有限公司

Sample File	Sample Name	Panel	SQO	OS	SQ
74_B10_Cellidentification-1-0819.fsa	YZY-003	21Plex_STR_Panel_v1.1			

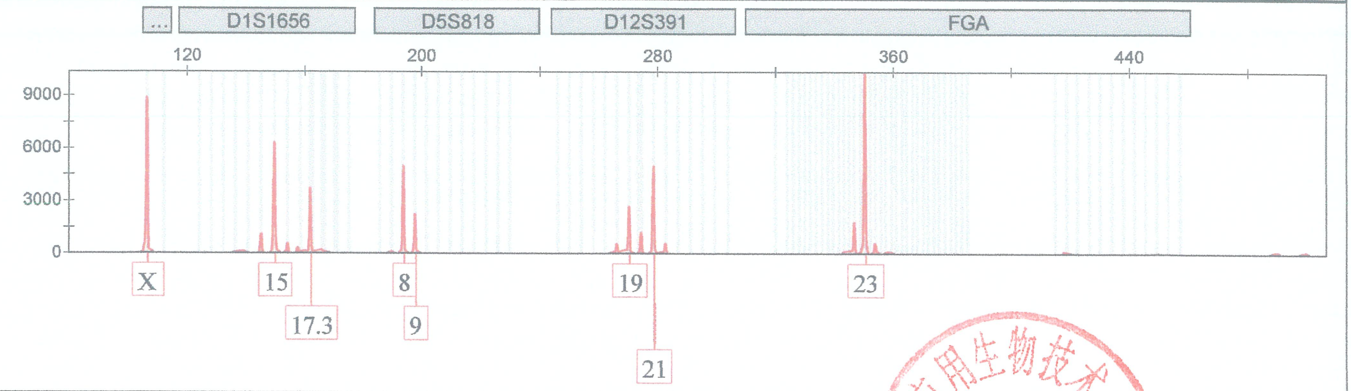

上海翼和应用生物技术有限公司
