# Supplementary figures and images for: CEMIP, acting as a scaffold protein for bridging GRAF1 and MIB1, promotes colorectal cancer metastasis via activating CDC42/MAPK pathway
Source: Cell Death Dis. 2023 Feb 27;14(2):167. doi: 10.1038/s41419-023-05644-z (PMC9971195; doi:10.1038/s41419-023-05644-z)

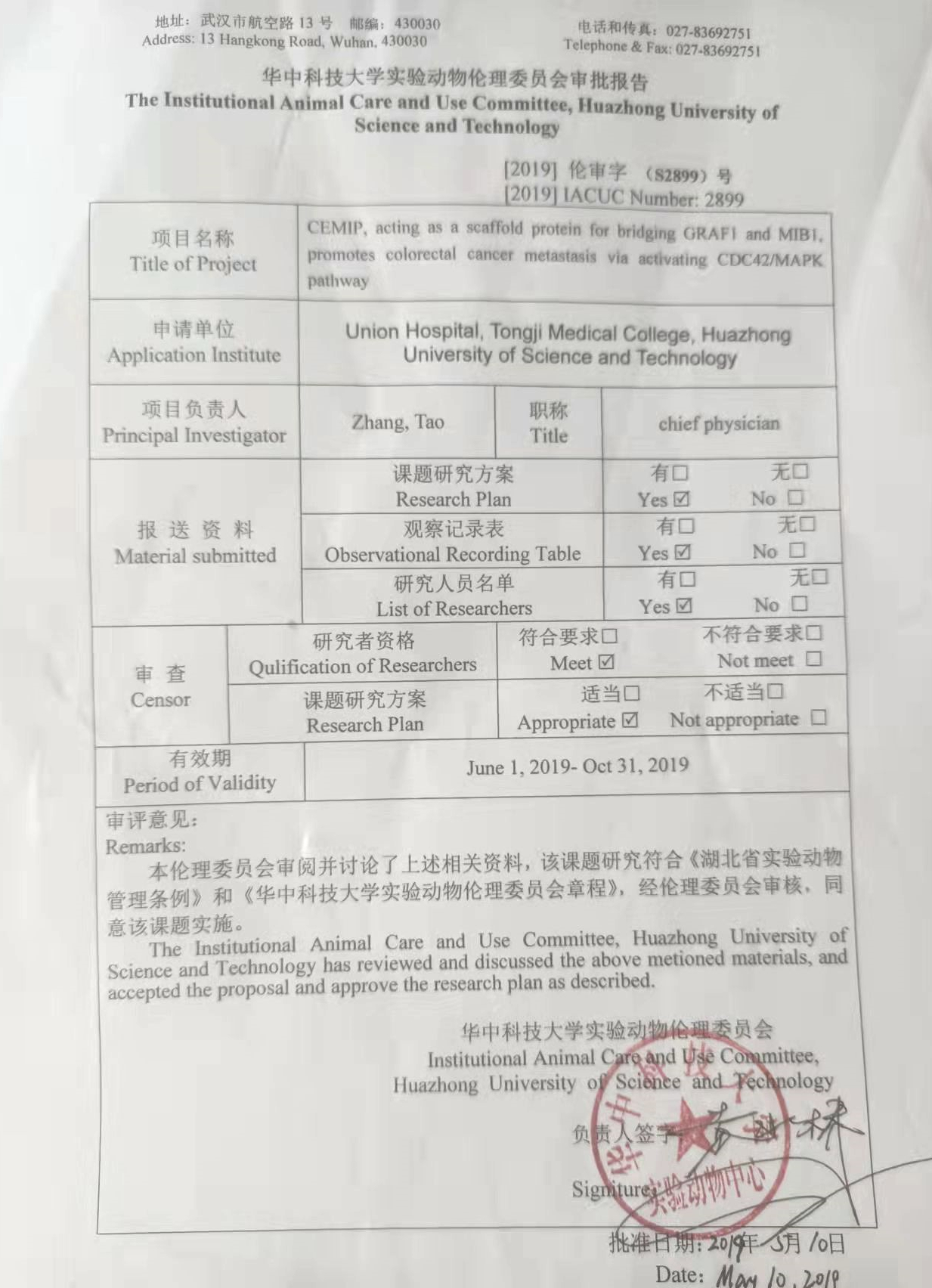

Supplement: Supplementary file 7 — Animal Ethics Provement [file 41419_2023_5644_MOESM7_ESM.jpg]

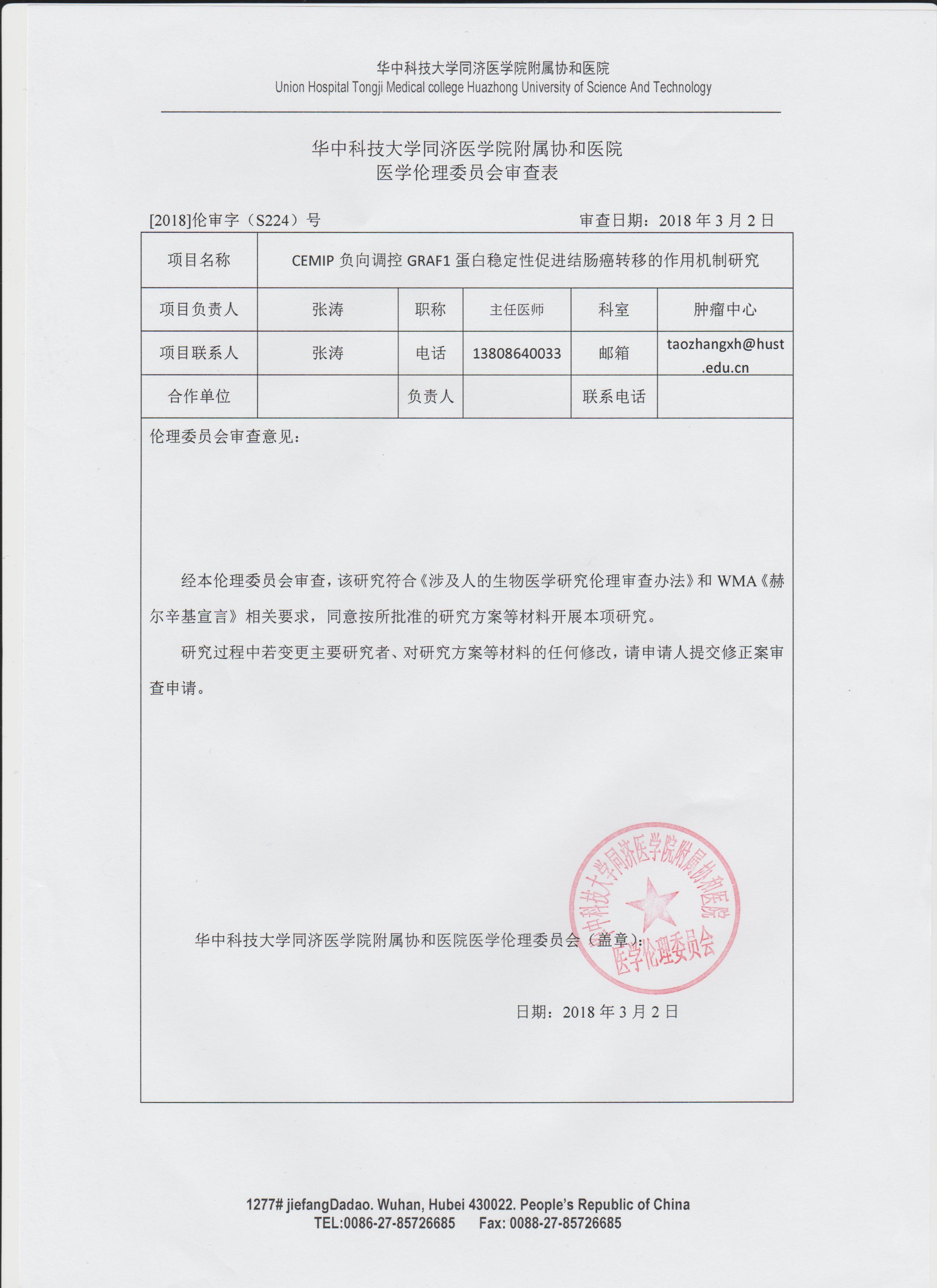

Supplement: Supplementary file 8 — Ethics Provement [file 41419_2023_5644_MOESM8_ESM.jpg]
